# Evolution of chemosensory and detoxification gene families across herbivorous Drosophilidae

**DOI:** 10.1093/g3journal/jkad133

**Published:** 2023-06-15

**Authors:** Julianne N Peláez, Andrew D Gloss, Benjamin Goldman-Huertas, Bernard Kim, Richard T Lapoint, Giovani Pimentel-Solorio, Kirsten I Verster, Jessica M Aguilar, Anna C Nelson Dittrich, Malvika Singhal, Hiromu C Suzuki, Teruyuki Matsunaga, Ellie E Armstrong, Joseph L M Charboneau, Simon C Groen, David H Hembry, Christopher J Ochoa, Timothy K O’Connor, Stefan Prost, Sophie Zaaijer, Paul D Nabity, Jiarui Wang, Esteban Rodas, Irene Liang, Noah K Whiteman

**Affiliations:** Department of Integrative Biology, University of California Berkeley, Berkeley, CA 94720, USA; Department of Biology, Brandeis University, Waltham, MA 02453, USA; Department of Ecology and Evolutionary Biology, University of Arizona, Tucson, AZ 85721, USA; Department of Biology and Center for Genomics and Systems Biology, New York University, New York, NY 10003, USA; Department of Integrative Biology, University of California Berkeley, Berkeley, CA 94720, USA; Department of Ecology and Evolutionary Biology, University of Arizona, Tucson, AZ 85721, USA; Department of Biology, Stanford University, Palo Alto, CA 94305, USA; Department of Ecology and Evolutionary Biology, University of Arizona, Tucson, AZ 85721, USA; National Center for Biotechnology Information, National Library of Medicine, National Institutes of Health, Bethesda, MD 20894, USA; Department of Integrative Biology, University of California Berkeley, Berkeley, CA 94720, USA; Department of Integrative Biology, University of California Berkeley, Berkeley, CA 94720, USA; Department of Biology, Stanford University, Palo Alto, CA 94305, USA; Department of Integrative Biology, University of California Berkeley, Berkeley, CA 94720, USA; Department of Ecology and Evolutionary Biology, University of Arizona, Tucson, AZ 85721, USA; Boyce Thompson Institute, Cornell University, Ithaca, NY 14853, USA; Department of Integrative Biology, University of California Berkeley, Berkeley, CA 94720, USA; Department of Chemistry & Biochemistry, University of Oregon, Eugene, OR 97403, USA; Department of Integrative Biology, University of California Berkeley, Berkeley, CA 94720, USA; Department of Integrative Biology, University of California Berkeley, Berkeley, CA 94720, USA; Department of Biology, Stanford University, Palo Alto, CA 94305, USA; Department of Ecology and Evolutionary Biology, University of Arizona, Tucson, AZ 85721, USA; Department of Integrative Biology, University of California Berkeley, Berkeley, CA 94720, USA; Department of Ecology and Evolutionary Biology, University of Arizona, Tucson, AZ 85721, USA; Department of Biology and Center for Genomics and Systems Biology, New York University, New York, NY 10003, USA; Department of Nematology, University of California Riverside, Riverside, CA 92521, USA; Department of Botany and Plant Sciences, University of California Riverside, Riverside, CA 92521, USA; Center for Plant Cell Biology and Institute for Integrative Genome Biology, University of California Riverside, Riverside, CA 92521, USA; Department of Ecology and Evolutionary Biology, University of Arizona, Tucson, AZ 85721, USA; Department of Biology, University of Texas Permian Basin, Odessa, TX 79762, USA; Department of Integrative Biology, University of California Berkeley, Berkeley, CA 94720, USA; Molecular Biology Institute, University of California Los Angeles, Los Angeles, CA 90095, USA; Department of Integrative Biology, University of California Berkeley, Berkeley, CA 94720, USA; Department of Integrative Biology, University of California Berkeley, Berkeley, CA 94720, USA; Department of Biology, Stanford University, Palo Alto, CA 94305, USA; Department of Ecology and Evolutionary Biology, University of Arizona, Tucson, AZ 85721, USA; Jacobs Institute, Cornell Tech, New York, NY 10044, USA; FIND Genomics, New York, NY 10044, USA; Department of Ecology and Evolutionary Biology, University of Arizona, Tucson, AZ 85721, USA; Department of Botany and Plant Sciences, University of California Riverside, Riverside, CA 92521, USA; Department of Integrative Biology, University of California Berkeley, Berkeley, CA 94720, USA; Department of Biomedical Engineering, Viterbi School of Engineering, University of Southern California, Los Angeles, CA 90007, USA; Department of Integrative Biology, University of California Berkeley, Berkeley, CA 94720, USA; Department of Integrative Biology, University of California Berkeley, Berkeley, CA 94720, USA; Department of Integrative Biology, University of California Berkeley, Berkeley, CA 94720, USA; Department of Molecular and Cell Biology, University of California Berkeley, Berkeley, CA 94720, USA

**Keywords:** *Scaptomyza*, gene family evolution, genomics, trophic shift, *Drosophila*, plant-herbivore interactions, adaptation

## Abstract

Herbivorous insects are exceptionally diverse, accounting for a quarter of all known eukaryotic species, but the genomic basis of adaptations that enabled this dietary transition remains poorly understood. Many studies have suggested that expansions and contractions of chemosensory and detoxification gene families—genes directly mediating interactions with plant chemical defenses—underlie successful plant colonization. However, this hypothesis has been challenging to test because the origins of herbivory in many insect lineages are ancient (>150 million years ago (mya)), obscuring genomic evolutionary patterns. Here, we characterized chemosensory and detoxification gene family evolution across *Scaptomyza,* a genus nested within *Drosophila* that includes a recently derived (<15 mya) herbivore lineage of mustard (Brassicales) specialists and carnation (Caryophyllaceae) specialists, and several nonherbivorous species. Comparative genomic analyses revealed that herbivorous *Scaptomyza* has among the smallest chemosensory and detoxification gene repertoires across 12 drosophilid species surveyed. Rates of gene turnover averaged across the herbivore clade were significantly higher than background rates in over half of the surveyed gene families. However, gene turnover was more limited along the ancestral herbivore branch, with only gustatory receptors and odorant-binding proteins experiencing strong losses. The genes most significantly impacted by gene loss, duplication, or changes in selective constraint were those involved in detecting compounds associated with feeding on living plants (bitter or electrophilic phytotoxins) or their ancestral diet (fermenting plant volatiles). These results provide insight into the molecular and evolutionary mechanisms of plant-feeding adaptations and highlight gene candidates that have also been linked to other dietary transitions in *Drosophila*.

## Introduction

The origin of land plants over 500 million years ago presented a new niche for early insects to colonize (Southwood 1972). The intimate relationship between plants and insects has since generated one of the most ecologically and evolutionarily dominant groups in Earth's history: the herbivorous insects. Herbivorous insects account for over a quarter of all known eukaryotic species and help form the basis of terrestrial food webs (Strong et al. 1984; Bernays 1998; Farrell 1998). It has long been hypothesized that the diversity of herbivorous insects emerged as a result of co-diversification processes with their host plants (Mitter et al. 1988; Farrell 1998; Marvaldi et al. 2002).

As proposed by Ehrlich and Raven (1964), herbivores can diversify by specializing in plants bearing the same chemical defenses through adaptations to chemical defenses (in one or a few plant families), that is, until new plant defenses evolve, allowing plants to escape and diversify under this release from herbivore pressure in this “co-evolutionary arms-race” (Ehrlich and Raven 1964). This theory of coevolution, commonly referred to as “escape and radiate” (Thompson 1988, 1994), has inspired much of the research on insect-plant interactions throughout the last several decades (Heidel-Fischer and Vogel 2015; Simon et al. 2015; Vertacnik and Linnen 2017). As a result, we have learned that genes that mediate interactions with plant secondary compounds, such as chemosensory and detoxification genes, likely underlie adaptive mechanisms for plant colonization (Ehrlich and Raven 1964; Berenbaum 1983; Li, Schuler et al. 2003; Futuyma and Agrawal 2009; Edger et al. 2015). Despite significant progress toward understanding the genetic basis of herbivore-plant interactions, the evolutionary processes shaping these large, complex, and rapidly evolving gene families are still not fully understood.

A principal source of the functional genetic variation that underlies dietary novelty in herbivorous arthropods arises from extensive gene family evolution. For example, the spider mite (*Tetranychus urticae*) and diamondback moth (*Plutella xylostella*) genomes harbor expanded gene families encoding enzymes involved in the detoxification of plant secondary compounds they encounter (Grbić et al. 2011; Dermauw et al. 2013; You et al. 2013). Similarly, genes encoding gustatory receptors (GRs) involved in host finding have experienced extensive lineage-specific duplications in butterflies (Briscoe et al. 2013). Despite the identification of gene family expansions and contractions in herbivorous insects (McBride 2007; Edger et al. 2015; Johnson et al. 2018), isolating herbivory as the cause remains controversial, as these changes may have resulted from subsequent specialization occurring over a hundred million years. For example, the most diverse extant herbivore lineages—butterflies and moths (Lepidoptera), as well as leaf beetles, weevils, and close relatives (Phytophaga)—arose in the late Paleozoic and early Mesozoic, respectively (Wiens et al. 2015; Kawahara et al. 2019). Parsing herbivore-specific effects from those resulting from specialization is particularly challenging given the prominent role of specialization in driving herbivore diversification rates. This is strongly supported by associations between host shifts and speciation events and by the higher species richness found among specialist herbivores compared to generalist herbivores (Futuyma and Agrawal 2009; Forister et al. 2015).

While it is unclear whether specialization on specific plant taxa evolves during or after the evolution of herbivory (Bernays 1998), many phytophagous insects nonetheless exhibit phylogenetic conservatism: associating with the same plant taxa for many millions of years (Futuyma and Agrawal 2009). Comparative genomic studies examining younger herbivore lineages would thus allow for a more refined analysis to identify herbivore-associated changes from those arising in response to specialization or other evolutionary forces (Gardiner et al. 2008; Yassin et al. 2016). Furthermore, most herbivore lineages lack the functional genetic tools necessary to examine the implications of these copy number changes. Here, we addressed these limitations by studying the evolution of herbivory within Drosophilidae using a comparative genomics approach.

**Fig. 1.**
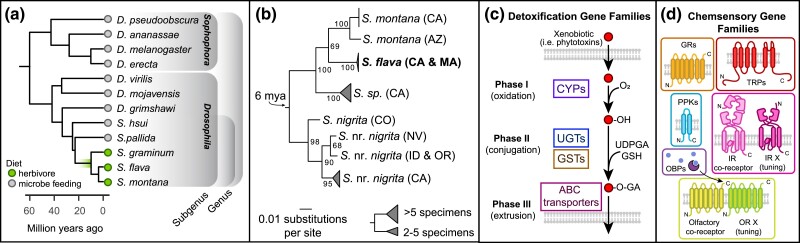
Gene families at the interface of plant-herbivore interactions. a) Phylogenetic placement of herbivorous *Scaptomyza* within the paraphyletic genus *Drosophila* (Matsunaga et al. 2022). b) ML nucleotide phylogeny built using *COI* sequences from North American *Scaptomyza* collected feeding on mustard plants (Brassicales spp.). Individuals with <1% pairwise nucleotide divergence collapsed into clades. Collection localities indicated by 2-letter state abbreviations. For this study, the genome of an *S. flava* line from MA, USA (bolded) was sequenced and assembled. Sequences and complete phylogeny are included in Supplementary Dataset 4 in Supplementary File 1. Divergence time estimated by Whiteman et al. 2012. c) Detoxification of xenobiotics/plant secondary metabolites (phytotoxins) involves 3 phases: oxidation/reduction by CYPs, enzymatic conjugation by UGTs or GSTs, and excretion/transport out of the cells by ATP-binding cassette transporters. d) Insects detect environmental compounds mostly with 6 major chemosensory gene families: GRs, OBPs, ORs, degenerin/epithelial sodium channels (PPKs), IRs, and TRPs.

Although larvae of most Drosophilidae species retain the ancestral habit of feeding on decaying plant tissue and associated microbes, herbivory has evolved several times in the lineage (Okada and Sasakawa 1956; Jones 1998; Whiteman et al. 2011; Maunsell et al. 2017; Durkin et al. 2021). A major clade of herbivorous species in the family are members of the genus *Scaptomyza*, a monophyletic lineage of ∼272 species and 21 subgenera (O’Grady and Desalle 2008). *Scaptomyza* spp. are nested within the paraphyletic subgenus *Drosophila,* which also includes Hawaiian *Drosophila* and the virilis-repleta radiation (Fig. 1a) (O’Grady and Desalle 2008; Lapoint et al. 2013; Katoh et al. 2017; O’Grady and DeSalle 2018; Church and Extavour 2022). Herbivorous *Scaptomyza* is found across the Holarctic, and *S. flava*, in particular, has been introduced into New Zealand, where it is a pest (Martin 2004). DNA barcoding revealed that the clade of herbivores may be cryptic radiation, with the divergence of 8 species in North America within the last ∼10 million years (Fig. 1b) (Katoh et al. 2017; Peláez et al. 2022). This is similar in species richness to the *D. melanogaster* subgroup worldwide (9 species within ∼12 million years) (David et al. 2007).

The herbivorous *Scaptomyza* species have become models for the evolution of herbivory and are an ideal group to test hypotheses about gene family evolution for several reasons. Most specialize in plants in the Brassicales (mustards and their relatives) and can be reared on the genetic model plant, *Arabidopsis thaliana* (Whiteman et al. 2011, 2012). Genetic dissection of adaptive traits in this lineage (Gloss et al. 2014; Goldman-Huertas et al. 2015; Peláez et al. 2022) has been enabled by the rich knowledge of gene function in *D. melanogaster*, a growing number of high-quality genomes across the drosophilid phylogeny (Drosophila 12 Genomes Consortium et al. 2007; Kim et al. 2021), strong phylogenetic frameworks (O’Grady and DeSalle 2018; Finet et al. 2021), and the ability to test hypotheses using genetic tools from both *Drosophila* and *Arabidopsis* (Groen and Whiteman 2016).

A critical advantage of studying *Scaptomyza* is that the genus encompasses species exhibiting varying degrees of specialization on different plant families: *S. graminum,* which specializes in plants of the Caryophyllaceae family (pinks or carnations), and *S. flava* as well as *S. montana,* which are largely specialists on Brassicales. The Brassicales harbor nontoxic glucosinolates that are the precursors to toxic mustard oils. While *S. flava* feeds on numerous genera of Brassicales, *S. montana* is more specialized, showing a strong preference for plants with indole glucosinolates (Gloss et al. 2017). *S. flava* has also been found to attack common pea plants (*Pisum sativum,* Fabaceae) and has further expanded its host range to some Caryophyllaceae in New Zealand (Martin 2004, 2012). Genetic changes shared by these 3 species may be more likely to be associated with the initial transition to herbivory, rather than their respective subsequent specializations. We cannot discount the possibility that these changes may be driven by other processes unrelated to herbivory. However, comparisons with closely related nonherbivores, such as *S. pallida* and *S. hsui*, each from different subgenera (*Parascaptomyza* and *Hemiscaptomyza*, respectively), allow us to differentiate herbivore-specific changes from those that arose earlier along with the *Scaptomyza* lineage. These 2 species are the closest relatives of the herbivorous species (∼20 million years diverged (Lapoint et al. 2013; Katoh et al. 2017; Peláez et al. 2022)) with genomic sequences available (Kim et al. 2021). Finally, nonherbivore specialists outside *Scaptomyza* can be used to further polarize patterns of specialization from those linked to herbivory. For instance, genome assemblies are available for *D. erecta*, a specialist on screwpine fruit (*Pandanus* spp.) and *D. mojavensis* that has populations specializing in rotting prickly pear cactus (*Opuntia littoralis*) (Rio et al. 1983; Pfeiler et al. 2005).

Given the salience of gene expansions and contractions associated with herbivorous insects, we hypothesized that genes involved in sensing or detoxifying plant chemical defenses would experience the greatest copy number changes. Specifically, in accordance with the neural limitation theory, we expected herbivores to lose numerous chemosensory genes, to streamline neural processing in the face of choosing between many host plants (Bernays 2001) and also their ancestral diet. We also expected to find duplicated genes with signatures of rapid protein evolution, which could have enabled early herbivores to gain new chemosensory or detoxification functions through neofunctionalization or subfunctionalization (Ohno 1970; Lynch and Conery 2000).

To address these hypotheses and predictions, we sequenced and assembled a high-quality genome sequence of *S. flava* to analyze alongside publicly available genome sequences of 2 herbivorous species (*S. montana* and *S. graminum*), 2 nonherbivores (*S. pallida* and *S. hsui*), and 7 nonherbivorous *Drosophila*, across a phylogenetic gradient from the herbivores (Fig. 1a). Following the curation of the major chemosensory and detoxification gene families (those in Fig. 1c and d), we then used maximum likelihood (ML) methods to test whether rates of gene gain and loss in herbivore branches differed from background branches. We next generated codon-based, ML models to identify genes with signatures of changing selective regimes along the branch at the base of the herbivore clade. We also evaluated whether our results may be explained by demographic events in recent history. We found that the most dramatic changes occurred in chemosensory genes involved in sensing yeast fermentation products/fruit volatiles and bitter/toxic plant chemical defenses.

## Materials and methods

### *S. flava* genome sequencing, assembly, and annotation

Full details of these methods can be found in the Supplementary Methods in Supplementary File 1.

#### PacBio and dovetail HiC libraries and sequencing

Sequence data for our main *S. flava* assembly (sfla_v2) were generated from a partially inbred laboratory colony. The colony was founded from >150 larvae collected near Dover, NH, USA, and subsequently maintained for several years in the laboratory. Three hundred male flies were flash frozen and stored at −80°C. SMRTbell libraries (∼20 kb) for PacBio Sequel were constructed using SMRTbell Template Prep Kit 1.0 (PacBio, Menlo Park, CA, USA) using the manufacturer's recommended protocol. Sequencing was performed on 2 PacBio Sequel SMRT cells. A Dovetail HiC library was prepared in a similar manner as described previously (Lieberman-Aiden et al. 2009). DNA was sheared to ∼350 bp mean fragment size, and sequencing libraries were generated using NEBNext Ultra enzymes and Illumina-compatible adapters. The libraries were sequenced on an Illumina HiSeqX to produce 380 million 2 × 150 bp paired-end reads, approximately 30× sequence coverage.

#### Illumina library and sequencing

Additional Illumina sequence data was used to polish the PacBio assembly. Sequences were generated from a laboratory population initially collected in Belmont, MA, USA in 2008 that was inbred through 10 generations of single-pair matings. Paired-end 180 and 300 bp insert libraries and 3 kbp and 5 kbp mate-pair libraries from female flies were sequenced with 100 bp read length on an Illumina Hiseq 2000 at the University of Arizona. Reads were quality filtered and Illumina TruSeq3 adapters were removed using Trimmomatic v0.35 (Bolger et al. 2014) with the following parameters: “LEADING:10 TRAILING:10 SLIDINGWINDOW:4:15 MINLEN:99”.

#### Genome assembly and scaffolding

The *S. flava* genome was assembled using the long-read hybrid assembly pipeline described in Kim et al. (2021) (https://github.com/flyseq/drosophila_assembly_pipelines), which has been shown to produce highly complete genome assemblies for drosophilid flies resulting in very few coding sequence indels (Kim et al. 2021). Following these methods, we generated an initial draft assembly with Flye v2.9 with default settings (Kolmogorov et al. 2019), and identified and removed duplicated haplotypes (haplotigs) with purge_haplotigs v1.1.1 (cutoffs: 3,33,195) (Roach et al. 2018). We polished the draft assembly using the PacBio reads with one round of Racon v1.4.3 (options: -m8 -x 6 g 8 w 500) (Vaser et al. 2017), then polished further using Illumina reads with one round of Pilon v1.23 (–fix snps, indels) (Walker et al. 2014), only fixing base-level errors. The fully polished assembly was scanned for contaminant sequences using NCBI BLAST v.2.10.0 (Johnson et al. 2008) and BlobTools (Laetsch and Blaxter 2017). Repetitive sequences in the assembly were identified with RepeatModeler2 (Flynn et al. 2020).

To integrate the long-range assembly information generated with HiC, we scaffolded the assembly with an initial assembly generated by Dovetail that was based on the PacBio and HiC libraries described above (full details included in the Supplementary Methods in Supplementary File 1). The Dovetail assembly exhibited high scaffold contiguity but contained a significant number of frameshift mutations in coding sequences that limited our ability to correctly annotate the assembly. We, therefore, scaffolded our less contiguous but more accurate assembly with the error-prone Dovetail version, reasoning that base-level errors in the previous version were unlikely to impact reference-based scaffolding. Briefly, the Dovetail assembly was performed using the FALCON 1.8.8 pipeline from PacBio with 70× SMRT data used as input. A cutoff length that corresponded to 50× coverage was used during the initial error-correcting stage, and error-corrected reads were aligned into 3,561 primary contigs. The assembly was then polished through PacBio's Arrow algorithm from SMRT Link 5.0.1, using the original raw-reads. This assembly and the HiC library were used as input for HiRise which uses proximity ligation data to scaffold genome assemblies (Putnam et al. 2016). Dovetail HiC library sequences were aligned to the input assembly using bwa (http://github.com/lh3/bwa). To scaffold our assembly with the Dovetail assembly, we soft-masked both genomes using the repeat library described above, using RepeatMasker (Smit et al. 2013). Then, we created a whole-genome alignment with Progressive Cactus (Armstrong et al. 2020) and used the RagOut reference-based scaffolder (Kolmogorov et al. 2014) to scaffold the new genome. The genome assembly of *S. flava* was scaffolded into 1,252 scaffolds covering 315.4 Mbp (N50 = 32.966 Mb) with a maximum length of 85.98 Mbp and 460 gaps.

#### Comparative annotation of *Scaptomyza* genomes

Gene annotations previously created for an Illumina-only *S. flava* assembly (sfla_v1) were transferred to the assemblies of *S. graminum, S. hsui, S. pallida, S. montana*, and the newest *S. flava* assembly (sfla_v2), using whole-genome Progressive Cactus alignments and the Comparative Annotation Toolkit (CAT (Fiddes et al. 2018)). Briefly, using the Illumina-only assembly (sfla_v1), repeat regions were masked using RepeatMasker (Smit et al. 2013) with the *Drosophila* repeat library. Protein-coding genes were annotated using MAKER2 (Holt and Yandell 2011), with the *S. flava* transcriptome (Whiteman et al. 2012) and predicted gene sequences from 12 *Drosophila* species (FlyBase release 2013_06) provided to inform gene models. The other 5 *Scaptomyza* assemblies were repeat-masked using repeat libraries generated with RepeatModeler2 and soft-masked using RepeatMasker. A published phylogeny for these species (Suvorov et al. 2022) was used as a guide tree for the whole-genome alignment. CAT was used to project annotations from sfla_v1 onto the other genomes. Further details of these methods are in the Supplementary Methods in Supplementary File 1.

### Gene model curation and orthology inference

An iterative curation strategy was used to identify the complement of chemosensory and detoxification genes in 4 published *Drosophila* genomes (*D. melanogaster, D. virilis, D. mojavensis,* and *D. grimshawi*) (Drosophila 12 Genomes Consortium et al. 2007), 4 published *Scaptomyza* genomes (*S. pallida, S. hsui, S. graminum,* and *S. montana*) (Kim et al. 2021), and in our *S. flava* assembly (sfla_v2). Genome assembly versions are listed in Supplementary Table 1 in Supplementary File 2, along with which species were included in each analysis. Gene curation included 6 chemosensory gene families (GRs, ionotropic receptors (IRs), olfactory receptors (ORs), odorant binding proteins (OBPs), pickpocket proteins (PPKs), transient receptor potential channels (TRPs)) and 3 detoxification gene families (Cytochrome P450s (CYPs), Glutathione S-transferases (GSTs), UDP-glycosyltransferases (UGTs)). First, all protein sequences in each family from *D. melanogaster* were queried against the assembled genomes and annotated proteomes of *D. virilis*, *D. mojavensis*, and *D. grimshawi*, using BLASTP (e-value cutoff <1e−3) (Altschul et al. 1997). The resulting collection of genes was then queried against the automated annotations for *Scaptomyza* species (e-value cutoff <1e−3). We iteratively ran BLAST searches using the identified genes from each species as queries against their genome assemblies until no new genes were identified. To validate putatively lost genes, we performed an additional TBLASTN search (e-value cutoff <1e−4). Genes were considered truly absent if this yielded no hits. Gene models significantly deviating in length from *D. melanogaster* orthologs were manually inspected, and corrected using aligned homologous sequences to the annotated genes in other species. Additional validation steps we implemented are described in the Supplementary Methods in Supplementary File 1. Nucleotide sequences were aligned in Geneious v.10.2.6 using MUSCLE (Edgar 2004) and manually inspected. All gene coordinates are provided in Supplementary Dataset 1 in Supplementary File 1.

To verify correct orthology assignments, ML gene trees were constructed from sequences from *D. grimshawi* and all *Scaptomyza*, using RAxML with default settings (Stamatakis 2006). Genes were binned into orthologous groups if they formed a clade with >70 bootstrap support. For poorly supported clades (bootstrap support <70), orthology groups were assigned based on previous orthology assignments (Low et al. 2007; Almeida et al. 2014; Good et al. 2014). Gene trees were midpoint rooted and visualized in iTOL v6 (Letunic and Bork 2021).

### Gene family size evolution

To identify chemosensory or detoxification gene families with rapid expansions and/or contractions among the herbivore lineages, we used the program computational analysis of gene family evolution (CAFE) v4.2.1 (Han et al. 2013), which models a birth-death process of gene gain and loss across a species tree. In addition to the 9 species mentioned above, we included 3 species of the subgenus *Sophophora* (*D. pseudoobscura*, *D. ananassae*, and *D. erecta*) to improve background rate estimates. We imported gene models for these species from published orthology annotations (Low et al. 2007; Almeida et al. 2014; Good et al. 2014). As input, we provided a matrix of gene counts for each orthologous gene cluster (Supplementary Dataset 2 in Supplementary File 1). Orthology groups were merged until there was at least 1 homologous gene reconstructed at the base of the phylogeny as the analysis assumes at least 1 ancestral gene per group. We used a published time-calibrated phylogeny of drosophilids (Matsunaga et al. 2022), which included all our species of interest, with the exception of *S. montana*, which was grafted onto this tree using a reported divergence time estimate from *S. flava* (Peláez et al. 2022).

First, we generated models of the average turnover rate (λ) (the ML estimate of the cumulative rate of gains and losses per gene per unit time). For each gene family, we compared a null model with a single λ rate estimated for all branches to a model with 2 rates: 1 rate estimated for a foreground branch, and another for the remaining background branches, where the foreground branch was the internal (ancestral) branch leading to all herbivores, a terminal branch leading to 1 of the 12 species, or the entire clade of herbivores. All models were run in triplicate, and the iteration with the highest ML probability was retained. To test whether a two-rate model was significantly better than the single-rate model, we performed likelihood ratio tests (LRTs). *P*-values were adjusted at a false discovery rate (FDR) of 1% using the *q*-value package in R (Storey 2008). Using the same methods, we also generated models estimating gains and losses (λ, µ) separately, rather than as a single parameter.

To test whether expansion and contraction rates of chemosensory and detoxification gene families deviated from genome-wide rates, we performed the same analyses on 200 randomly chosen orthology groups. We identified these gene sets using the OrthoVenn2 server (Wang et al. 2015; Xu et al. 2019), a web-based orthology assignment tool based on OrthoMCL (Li, Stoeckert et al. 2003). We uploaded genome-wide proteomes for the 5 *Scaptomyza* species and used protein sequences for the remaining 7 species available through OrthoVenn2 (Ensembl database, release January 2019). Default parameters were used with an e-value cutoff of 0.05 and an inflation value of 1.5. Clusters were randomly chosen that summed up to ∼200 genes per species.

### Molecular evolutionary analysis

To identify genes that experienced changes in selective pressure along the ancestral branch at the base of the herbivorous species, we used codon-based models of evolution (*codeml* program) in the phylogenetic analysis by maximum likelihood (PAML) package (Yang 2007). These models estimate the nonsynonymous (dN) to synonymous (dS) substitution rate ratio (ω = dN/dS), wherein protein-coding genes experiencing positive directional selection may accumulate a significant number of amino acid substitutions resulting in dN/dS > 1, those evolving neutrally dN/dS ≈ 1, and those experiencing negative or purifying selection dN/dS < 1.

For each group of orthologous genes, nucleotide sequences from 9 taxa (*S. flava, S. montana, S. graminum, S. pallida, S hsui, D. grimshawi, D. mojavensis, D. virilis*, and *D. melanogaster*) were aligned in Geneious v.10.2.6 using MAFFT v7.450 translation alignment with default settings (Katoh et al. 2002; Katoh and Standley 2013). A species tree was generated for these analyses by summarizing the 100 ML-generated gene trees published by (Kim et al. 2021), using ASTRAL v.5.5.9 (Zhang et al. 2018) with default settings. For genes with multiple paralogs, we estimated gene trees using FastTreeMP v2.1 with a general time reversible model with gamma rate distribution and 100 bootstrap replicates (Price et al. 2010).

We employed branch, branch-site, and clade models within PAML. We used the branch model to assess whether background and foreground dN/dS rates differ across branches and whether this could be attributed to positive selection, reduced purifying selection, or stronger purifying selection. (Yang 1998). We compared a “two-ratio” branch model, where dN/dS is estimated separately for a specified foreground branch and background branches (model = 2, NSites = 0), against a null model (“M0”), which estimates a single dN/dS rate over all branches (model = 0, NSsites = 0). To test whether the foreground branch experienced positive selection (dN/dS > 1), we compared the two-ratio model to a model in which ω is fixed to 1 (model = 2, NSites = 0, fix_omega = 1).

We also used branch-site models, which more realistically model protein evolution, allowing dN/dS to vary across both codons and branches (Yang and Nielsen 2002; Zhang et al. 2005). We compared the “branch-site model A” (model = 2, NSsites = 2, fix_omega = 0) against a null model in which ω_2_ is fixed to 1 (model = 2, NSsites = 2, fix_omega = 1). This comparison also offers a direct test for positive selection.

Finally, we used the “Clade model C” (CmC) to estimate changing selection pressures when genes exhibited numerous paralogs within species (Weadick and Chang 2012). Based on branch-site models, CmC model allows dN/dS to vary in a proportion of sites between 2 gene clades, and can detect more subtle divergent selection, particularly after duplication events (Bielawski and Yang 2004; Weadick and Chang 2012). We tested for divergent selection by comparing the CmC model (model = 3, NSsites = 2, fixed = 0) against the null model “M2a_rel” (model = 0, NSsites = 22, fixed = 0), which has the same number of site classes as the CmC model but does not vary across branches. To test for positive selection on the ancestral herbivore branch, we compared the CmC model against a null model in which the ω of the divergent site class is constrained to 1 (model = 3, NSsites = 2, fixed = 1) (Van Nynatten et al. 2015). For clades with foreground divergent sites significantly deviating from the null expectation, we then performed branch and branch-site tests to test which set of paralogs experienced divergent selection.

For all PAML analyses, model comparisons were made using LRTs with a χ^2^ distribution and 1 degree of freedom. *P*-values were adjusted at a FDR of 5% using the q-value package in R (Storey 2008).

## Results

### *S. flava* genome assembly and annotation

To complement existing genome assemblies of nonherbivorous and herbivorous drosophilids, we sequenced, assembled, and annotated the genome of *Scaptomyza flava*, a leaf-mining specialist on mustards. Our main assembly (sfla_v2) contained 781 scaffolds with an N50 of 31.83 Mb and an assembly size of 331.7 Mb (Supplementary Table 2 in Supplementary File 2). Using gene models from other *Drosophila* species and transcriptome sequences across *S. flava* life stages, we generated annotations for the *S. flava* assembly sfla_v1, which were carried over to our main assembly sfla_v2, amounting to 12,365 predicted genes. We performed the same carry-over procedures on 4 other *Scaptomyza* species (*S. pallida, S. hsui, S. montana,* and *S. graminum*) to facilitate the annotation of their chemosensory and detoxification gene families. The completeness of the assemblies was assessed by comparing the assembly against the Benchmarking Universal Single-Copy Orthologs (BUSCO) dipteran database. The genome assembly of *S. flava* is near complete, as are the assemblies of the 4 other *Scaptomyza* species with values comparable to the published assemblies of the 7 other *Drosophila* species included in subsequent analyses (*D. melanogaster, D. erecta, D. ananassae, D. pseudoobscura, D. mojavensis, D. virilis,* and *D. grimshawi*) (Supplementary Fig. 1a in Supplementary File 3). Approximately 98.8% of complete BUSCO gene models (98% single-copy and 0.8% duplicated) were identified from the sfla_v2 assembly, 0.4% were found fragmented, and 0.8% were not found. BUSCO scores were also high for the genome-wide automated annotations in all *Scaptomyza* species (Supplementary Fig. 1b in Supplementary File 3). The completeness of the genome assemblies and automated annotations provided confidence that we would be able to reliably assess the evolution of our gene families of interest.

### Contractions and expansions of chemosensory and detoxification gene families

We analyzed the evolution of chemosensory and detoxification gene families to determine if rates of gain, loss, and/or turnover (cumulative gains and losses) were significantly different between nonherbivorous species and herbivorous *Scaptomyza* species. We used CAFE, a program that makes ML estimates of gene copy number evolutionary rates, (Han et al. 2013) and included 9 nonherbivorous and 3 herbivorous species (all species in Fig. 1a), all of which use a range of different feeding substrates with varying degrees of specialization. While we tested whether rates were significantly higher along terminal herbivore branches and across the entire herbivore clade, compared to the remainder of the tree, we were particularly interested in significantly higher copy number changes along the ancestral herbivore branch. We manually curated 6 chemosensory gene families (GRs, IRs, ORs, OBPs, pickpocket proteins, PPKs, TRPs) and 3 detoxification gene families (CYPs, GSTs, UGTs) through iterative BLAST searches and additional validation steps (described in the Supplementary Methods in Supplementary File 1) to confirm the loss or duplication of genes (gene coordinates: Supplementary Dataset 1 in Supplementary File 1, RAXML gene trees: Supplementary Figs. 2–10 in Supplementary File 3).

**Fig. 2.**
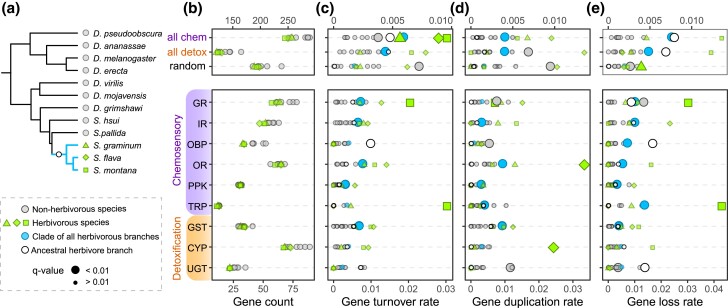
The clade of *Scaptomyza* herbivores (but not their ancestral lineage) exhibit elevated gene turnover within many chemosensory and detoxification gene families. a) Phylogeny of drosophilids included in CAFE analyses: 3 herbivorous and 9 nonherbivorous *Drosophila*. b) Gene counts per gene family. Evolution of gene family size was estimated by ML in CAFE, where the rates of (c) gene turnover, λ (cumulative gains and losses), d) gene duplication, λ, and (e) gene loss, µ, were estimated for each species. Points represent the rate of a foreground branch or clade from models in which it was allowed to evolve at a rate separate from background branches. Each model was compared to a null model (single-rate estimated for the entire phylogeny) using a likelihood ratio test at an analysis-wide FDR of 1% (*q* < 0.01). Foreground branches with significantly higher rates than the rest of the phylogeny are indicated by larger shapes. Full details can be found in Supplementary Tables 3–5. All chem = all chemosensory genes. All detox = all detoxification genes. Random = random set of 200 orthology groups.

Overall, herbivorous species had smaller repertoires of detoxification and chemosensory genes than nonherbivorous species. (Fig. 2b, Supplementary Table 3 in Supplementary File 2). The rate of gene turnover was significantly higher along the ancestral herbivore branch, than the background branches when all chemosensory genes were considered (λ_anc_herb_ = 0.005; λ_bkgrd_ = 0.002; q-value = 0.005) but not for all detoxification genes (λ_anc_herb_ = 0.005; λ_bkgrd_ = 0.002; q-value > 0.05) (Fig. 2c, Supplementary Table 4 in Supplementary File 2). When individual gene families were analyzed, the ancestral herbivore branch only experienced significantly higher rates of turnover among OBPs. We also estimated rates of gene duplication and loss separately, finding that along the ancestral herbivore branch the gene duplication rate was not significantly higher for any gene family compared to background rates (Fig. 2d, Supplementary Table 5 in Supplementary File 2).

The gene loss rate, however, was significantly higher for all chemosensory genes (λ_anc_herb_ = 0.008; λ_bkgrd_ = 0.002; q-value < 0.001) and detoxification genes (λ_anc_herb_ = 0.007; λ_bkgrd_ = 0.002; q-value = 0.003) (Fig. 2e, Supplementary Table 5 in Supplementary File 2). Specifically, higher rates of gene loss along the ancestral herbivore branch were found among GRs, OBPs, and UGTs. The CAFE analysis also allowed us to identify specific genes that experienced expansions and contractions (Supplementary Table 6 in Supplementary File 2; Supplementary Fig. 11 in Supplementary File 3). Significant contractions along the ancestral herbivore branch included OBPs (*Obp18a, Obp58b,* and *Obp58c*) and *Or22a*, an ester-sensitive, yeast-volatile receptor. The only expansion was of *Ir67a*, which was duplicated in the ancestral herbivorous lineage.

While we found limited copy number changes along the ancestral herbivore branch, rates of gene turnover, duplication, and loss estimated across the entire herbivore clade were significantly higher than background rates for numerous gene families (gene turnover: GRs, IRs, ORs, PPKs, and GSTs (Fig. 2c); duplication: IRs, ORs, PPKs, TRPs, and GSTs (Fig. 2d); loss: GRs, IRs, OBPs, ORs, PPKs, TRPs, and GSTs (Fig. 2e)). We confirmed that it is unlikely that these elevated rates among the herbivorous species could be attributed to the longer branch length of the ancestral branch preceding the herbivore clade. We performed another set of CAFE analyses to compare rates between 2 clades of similar age: the clade of *D. melanogaster* and *D. erecta* (28 million years old) vs the clade of *S. flava* and *S. graminum* (23 million years old). This confirmed that the former pair showed no gene families evolving at higher rates, whereas the clade of *S. flava* and *S. graminum* still showed elevated rates of turnover, loss, and gain (Supplementary Fig. 12 in Supplementary File 3).

**Fig. 3.**
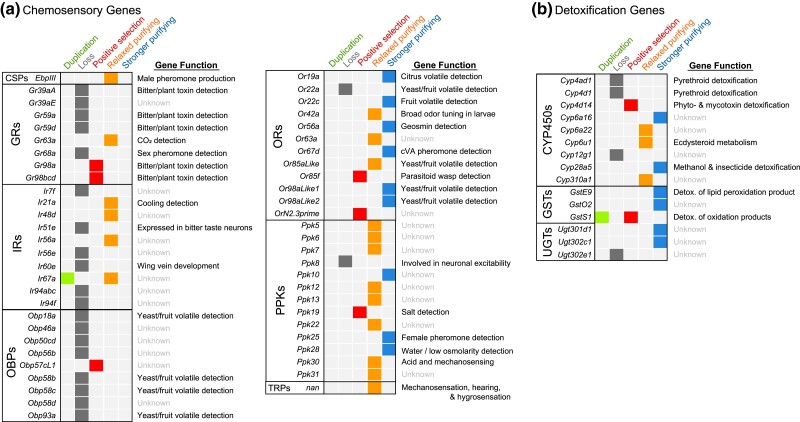
Genes involved in the detection of bitter compounds and yeast/fruit odorants disproportionately experienced gene loss or divergent selection in the ancestral *Scaptomyza* herbivore lineage. Summary of (a) chemosensory and (b) detoxification genes that were duplicated, lost, or experienced divergent selection (positive selection or relaxed or stronger purifying selection). Gene duplications and losses are based on gene trees generated through RAXML, and genes that experienced changing selection pressures were identified with PAML. Gene counts from each species are shown in Supplementary Table 7 in Supplementary File 2. Gene functions based on published data for *D. melanogaster* orthologs (sources can be found in Supplementary Table 10 in Supplementary File 2).

Based on the RAxML trees for each gene family (Supplementary Figs. 2–10 in Supplementary File 3), there were many herbivore-specific losses, in addition to a few gains (summarized in Fig. 3; Supplementary Table 7 in Supplementary File 2 for gene counts). Most copy number changes shared by all herbivores were concentrated among chemosensory gene families (22 out of 27). Herbivores lost several GRs that are required for bitter reception or are expressed in bitter gustatory neurons in *D. melanogaster* (multiple paralogs of isoform A of *Gr39a* [*Gr39aA*], *Gr59a, Gr59d*) (Kwon et al. 2011; Dweck and Carlson 2020). Herbivorous lineages also lost *Gr68a*, which is involved in the detection of an anti-aphrodisiac (Bray and Amrein 2003), and *Gr39aE* which has no known ortholog in *D. melanogaster*. The only OR lost was the aforementioned *Or22a*, which has been previously reported (Goldman-Huertas et al. 2015). Among the OBPs, almost all of the herbivore-specific losses (*Obp46a, Obp50cd, Obp58b, Obp58c, Obp58d, Obp93a*) were among the “plus-C OBPs,” which are characterized as having more than 6 cysteine residues, and only 2 losses (*Obp18a* and *Obp56b*) were among the “classic OBPs’ with 6 cysteines (Supplementary Fig. 13 in Supplementary File 3). Strikingly, nonherbivorous *Scaptomyza* has 11 Plus-C OBPs, while the herbivores possess only five. Almost all IRs that were lost in the herbivores belonged to the “divergent IR” class (*Ir7f, Ir51e, Ir56e, Ir94abc, Ir94f*), which are expressed in gustatory neurons. The only “antennal IR” lost in all herbivores was *Ir60a,* and the only duplication was the divergent IR *Ir67a*. A single loss was found among PPKs (*ppk8*), whose function is unknown. Expression localization of *D. melanogaster* chemosensory gene orthologs that were lost or duplicated in all herbivores is presented in Supplementary Fig. 14 in Supplementary File 3.

Only 5 herbivore-specific changes in copy number were found among the detoxification gene families: the duplication of *GstS1*, which has a role in oxidative stress responses and in flight muscle structure (Singh et al. 2001); the loss of *Ugt302e1*, whose function is currently unknown, and 3 CYPs (*Cyp4ad1, Cyp4d1,* and *Cyp12g1*). While there is no ortholog of *Cyp12g1* in *D. melanogaster*, both *Cyp4ad1* and *Cyp4d1* are upregulated in response to the pyrethroid deltamethrin (Liu et al. 2020).

### Genes under divergent selection in the herbivore lineage

We next used an ML approach through PAML to identify chemosensory and detoxification genes with signatures of changes in the selection regime in the branch leading to the herbivorous species. We focused on this ancestral herbivore branch, rather than those leading to individual herbivore species, to identify changes that occurred as a result of or coincident with herbivory, rather than subsequent specialization on mustard or carnation plants. We used codon-based tests for positive selection, estimating dN/dS values under branch, branch-site, and clade models. Clade models offer more sensitivity in detecting divergent selection among a clade of genes, particularly those with multiple recent paralogs undergoing complex evolution (Weadick and Chang 2012). For genes that showed evidence of divergent selection through the use of clade models (*P* < 0.05; FDR < 5%), we additionally tested branch and branch-site models on branches leading to different paralogs to identify which paralog experienced significant changing selection pressure. We report significant results from the initial branch and branch-site tests and significant results from clade model follow-up tests. In total, 44 genes were found to have experienced a significant change in selection pressure along the basal herbivore branch (Tables 1 and Supplementary Table 8). Results on all genes can be found in Supporting Supplementary Dataset 3 in Supplementary File 1.

**Table 1.** Summary of selection analyses on chemosensory and detoxification genes.

Gene	Model	dN/dS		LRT 2(ΔlnL)	*q-*value *(FDR 5%)*
***Chemosensory Genes***					
*Csp2*	Branch	ω0 = 0.06	ω1 = 0.24	7.14	0.05
*Gr63a*	Branch-site	ω0 = 0.03 (81%)	ω1 = 1 (7%)	16.52	<0.01
		ω2a = 1 (11%)	ω2b = 1 (1%)		
*(SmonGr98a1, SflaGr98a1, SgraGr98a2)*	Branch	ω0 = 0.29	ω1 = 0.87	7.74	0.04
	Branch-site	ω0 = 0.22 (71%)	ω1 = 1 (26%)	11.4	0.03
		ω2a = 32.44 (2%)	ω2b = 32.44 (1%)		
*Gr98bcd*	Branch-site	ω0 = 0.16 (73%)	ω1 = 1 (27%)	10.2	0.05
		ω2a = 179.55 (0.004%)	ω2b = 179.55 (0.001%)		
*Ir21a*	Branch	ω0 = 0.11	ω1 = 0.26	9.2	0.02
*Ir48d*	Branch	ω0 = 0.18	ω1 = 0.38	7.96	0.04
*(SflaIr56a, SmonIr56a)*	Branch	ω0 = 0.33	ω1 = 0.98	18.8	<0.001
*(SgraIr56a1, SgraIr56a2, SgraIr56a4, SgraIr56a5)*	Branch	ω0 = 0.33	ω1 = 3.66	7.98	0.03
	Branch (positive)	ω0 = 0.34	ω1 = 1	1.56	0.31
*Ir67a*	Branch	ω0 = 0.3	ω1 = 0.6	10.6	0.01
*Obp57cL1*	Branch-site	ω0 = 0.21 (59%)	ω1 = 1 (36%)	15.68	<0.01
		ω2a = 148.51 (3%)	ω2b = 148.51 (2%)		
*Or19a*	Branch	ω0 = 0.3	ω1 = 0.09	13.7	<0.01
*Or22c*	Branch	ω0 = 0.18	ω1 = 0.05	7.24	0.05
*(SflaOr42a1, SmonOr42a1, SgraOr42a1)*	Branch	ω0 = 0.13	ω1 = 0.45	11.46	<0.01
*Or56a*	Branch	ω0 = 0.11	ω1 = 0.03	8.98	0.02
*Or63a*	Branch	ω0 = 0.2	ω1 = 0.76	29.5	<0.0001
*Or67d*	Branch	ω0 = 0.18	ω1 = 0.03	15.18	<0.01
*Or85aLike*	Branch	ω0 = 0.13	ω1 = 0.29	6.98	0.05
*Or85f*	Branch-site	ω0 = 0.13 (88%)	ω1 = 1 (11%)	12.54	0.03
		ω2a = 46.61 (1%)	ω2b = 46.61 (0.001%)		
*Or98aLike1*	Branch	ω0 = 0.19	ω1 = 0.05	7.58	0.04
*Or98aLike2*	Branch	ω0 = 0.2	ω1 = 0	35.92	<0.0001
*OrN2.3prime*	Branch-site	ω0 = 0.16 (73%)	ω1 = 1 (20%)	15.6	<0.01
		ω2a = 15.3 (5%)	ω2b = 15.3 (1%)		
*Ppk5*	Branch	ω0 = 0.11	ω1 = 0.36	15.16	<0.01
*Ppk6*	Branch	ω0 = 0.09	ω1 = 0.25	10.35	0.01
*Ppk7*	Branch	ω0 = 0.15	ω1 = 0.27	7.75	0.04
*Ppk10*	Branch	ω0 = 0.1	ω1 = 0.01	12.12	<0.01
*Ppk12*	Branch	ω0 = 0.18	ω1 = 0.38	10.07	0.02
*Ppk13*	Branch	ω0 = 0.05	ω1 = 0.19	16.12	<0.01
*Ppk19*	Branch-site	ω0 = 0.11 (75%)	ω1 = 1 (23%)	13.98	0.01
		ω2a = 15.75 (2%)	ω2b = 15.75 (1%)		
*Ppk22*	Branch	ω0 = 0.16	ω1 = 0.37	7.7	0.04
*Ppk25*	Branch	ω0 = 0.21	ω1 = 0.04	16.82	<0.01
*Ppk28*	Branch	ω0 = 0.1	ω1 = 0.03	11.72	<0.01
*Ppk30*	Branch	ω0 = 0.2	ω1 = 0.44	12.2	<0.01
*Ppk31*	Branch	ω0 = 0.12	ω1 = 0.39	17.23	<0.01
*nan*	Branch	ω0 = 0.03	ω1 = 0.09	10.66	0.01
***Detoxification Genes***					
*Cyp28a5*	Branch	ω0 = 0.13	ω1 = 0.05	9.95	0.02
*Cyp310a1*	Branch	ω0 = 0.19	ω1 = 0.57	18.43	<0.001
*Cyp4d14*	Branch-site	ω0 = 0.06 (84%)	ω1 = 1 (15%)	11.44	0.03
		ω2a = 809.22 (1%)	ω2b = 809.22 (0.001%)		
*Cyp6a16*	Branch	ω0 = 0.21	ω1 = 0.1	7.57	0.04
*Cyp6a22*	Branch	ω0 = 0.07	ω1 = 0.26	21.17	<0.001
*Cyp6u1*	Branch	ω0 = 0.14	ω1 = 0.61	20.06	<0.001
*GstE9*	Branch	ω0 = 0.15	ω1 = 0.01	9.35	0.02
*GstO2*	Branch	ω0 = 0.12	ω1 = 0	10.3	0.01
*(SflaGstS1b, SmonGstS1b, SgraGstS1b)*	Branch	ω0 = 0.09	ω1 = 3.27	50.43	<0.0001
	Branch-site	ω0 = 0.08 (84%)	ω1 = 1 (2%)	14.04	0.01
		ω2a = 38.47 (13%)	ω2b = 38.47 (0.004%)		
*Ugt301D1*	Branch	ω0 = 0.09	ω1 = 0.03	7.22	0.05
*Ugt302C1*	Branch	ω0 = 0.11	ω1 = 0.04	7.42	0.05

PAML analyses under branch and branch-site models. Branch model: M0 vs 2-ratios; branch model (positive selection): 2-ratios vs 2-ratios (ω1 = 1); branch-site model: Model A vs Model A (ω2 = 1). Omega values are reported only for the alternative model. Detailed results can be found in Supplementary Table 8 in Supplementary File 2.

Eight genes were identified as having experienced positive selection (dN/dS >1) in the basal herbivore lineage: *Gr98a, Gr98bcd, GstS1b, Obp57cL1, Or85f, OrN2.3prime, Cyp4d14*, and *Ppk19* (Table 1). The proportion of positively selected sites among these genes was generally low (mean ± SD, 4% ± 4.17), with a few exceptions. One such exception was *GstS1b*, which exhibited 13% positively selected sites (dN/dS = 38.47), almost all of which had exceptionally strong support (i.e. high posterior probabilities, *P* > 0.95). As noted earlier, *GstS1* was duplicated in the ancestor of herbivorous *Scaptomyza*. The single ortholog in *D. melanogaster* is involved in oxidative stress response, conjugation of the lipid peroxidation product 4-hydroxynonenal, and modulating methylmercury toxicity (Singh et al. 2001; Whitworth et al. 2005; Vorojeikina et al. 2017). Despite the expression of both *GstS1a* and *GstS1b* in the *S. flava* larval gut (Gloss et al. 2019), positive selection on only *GstS1b* indicates potential neofunctionalization of this copy. Notably, mustard-derived toxins (isothiocyanates) induce the formation of peroxidized lipids (Gago-Dominguez et al. 2007).

Two genes (*Gr98a* and *Gr98bcd*) that experienced positive selection along the ancestral herbivore lineage are involved in the detection of noxious, “bitter” compounds. The *D. melanogaster* ortholog of *Gr98a* is involved in detecting histamine, which is high in fermented foods (Aryal and Lee 2022) and *Gr98b* detects L-canavanine, an extremely bitter plant-derived toxic amino acid (Shim et al. 2015).

Among genes that did not show a strong indication of positive selection but were found to be evolving at significantly different rates between herbivores and background branches, many (20/35) had higher foreground dN/dS values (Table 1), indicating relaxation from selective constraints among the herbivores, whereas the remaining experienced stronger purifying selection. Many ORs in *D. melanogaster* have been de-orphanized, providing a wealth of information about the breadth and sensitivity of individual ORs (e.g. Hallem et al. 2004). Though the properties of *D. melanogaster*'s ORs cannot be used to precisely determine those of other species, it offers a starting point to generate hypotheses about how ORs are evolving in closely related species. We found that almost all ORs experiencing relaxed purifying selection (*Or42a1, Or85aLike*) or stronger purifying selection (Or22c, *Or98aLike1*, *Or98aLike2*) detect esters and alcohols, which are produced in high abundance by yeasts and fruit, and are attractive to microbe-feeders (Becher et al. 2012), but not herbivores (Goldman-Huertas et al. 2015; Matsunaga et al. 2022). Additionally, there was a stronger selection of *Or56a*, and *Or19a*, which detect geosmin and terpenes, respectively (Stensmyr et al. 2012; Dweck et al. 2013).

Detoxification genes that experienced relaxed purifying selection in the ancestral herbivore lineage were *Cyp310a, Cyp6a22,* and *Cyp6u1*, and those that experienced stronger purifying selection were *GstE9, GstO2, Cyp28a5, Cyp6a16, Ugt301d1,* and *Ugt302c1* (Table 1). The majority of these have been found to be upregulated in response to toxin consumption, although some have putative functions in development, hormone metabolism, cold tolerance, and olfaction (Fig. 3).

### Effects of demographic history

Demographic processes, such as population bottlenecks, can weaken the efficacy of natural selection, leading to an accelerated fixation of slightly deleterious gene gains and losses (Gardiner et al. 2008). To investigate the possibility that our results on gene family evolution were confounded by demographic events, we examined 3 lines of evidence from our analyses.

First, the randomly selected set of orthologous clusters (∼200 genes) exhibited gain, loss, and turnover rates that were not significantly higher among herbivores than in other *Drosophila* (λ_herb_ = 0.002; λ_nonherb_ = 0.003; q-value <0.02, Fig. 2). These results suggest demographic processes were not the underlying cause of elevated rates of turnover among chemosensory and detoxification genes because these demographic processes would have generated similar patterns genome-wide.

Second, we inferred the level of nucleotide diversity (π) in an *S. flava* population (collected in 2013 from Belmont, MA, USA), using pooled whole-genome sequencing (methods described in the Supplementary Methods in Supplementary File 1). Autosomal nucleotide diversity, which is proportional to the coalescent effective population size, was similar between a single population of *S. flava* (π = 0.0056) and North American populations of *D. melanogaster* (the *D. melanogaster* Genetic Reference Panel; π = 0.0060 (Mackay et al. 2012)). The relationship between physical distance and linkage disequilibrium was also similar between the 2 species and decayed quickly (Peláez et al. 2022), consistent with sharing similarly large coalescent effective population sizes (Sved 1971).

Third, we estimated the proportion of the genome composed of repetitive elements, which is sensitive to demographic shifts (Bourgeois and Boissinot 2019), and found that repeat content in *S. flava* is within the range observed across *Drosophila* species (Supplementary Table 9 in Supplementary File 2). These analyses do not preclude the possibility that demography has contributed to the gene family patterns observed, but they all suggest that herbivorous *Scaptomyza* do not have dramatically atypical demographic histories compared to other *Drosophila*.

## Discussion

In this study, we sought to determine the extent to which the evolution of herbivory was correlated with patterns of molecular evolution across chemosensory and detoxification gene families in the Drosophilidae. We focused on a clade within the genus *Scaptomyza*—nested within the paraphyletic genus *Drosophila—*that evolved to feed as both leafminers (larvae) and leaf puncture-feeding adults (females) on living plants <15 mya (Fig. 1a). A great deal is known regarding how these gene families, especially chemosensory ones, evolve across *Drosophila* species in the context of diet shifts and diet specialization (McBride 2007; Gardiner et al. 2008; Rane et al. 2019; Reisenman et al. 2023). However, we have far less insight into how these gene families evolve in response to a truly herbivorous niche shift (i.e. larval development is completed by feeding on living plants).

Here, we identified, through iterative curation and manual inspection, the full complement of several chemosensory and detoxification gene families for 5 *Scaptomyza* species—3 herbivorous species within the subgenus *Scaptomyza* (*S. flava*, *S. montana*, and *S. graminum*) and 2 nonherbivorous species from 2 other subgenera (*S. pallida* in *Parascaptomyza* and *S. hsui* in *Hemiscaptomyza*)—and more distantly related *Drosophila* species. By including species across a range of diets and phylogenetic distances, we were able to disentangle changes specific to the herbivorous lineages from those shared across the genus *Scaptomyza* and those associated with more recent host plant-specific specialization (*S. flava* and *S. montana* on Brassicales and *S. graminum* on Caryophyllaceae). Some limitations of this study are that only a single herbivore lineage was included and that some genetic changes may be driven by other evolutionary processes unrelated to the evolution of herbivory. We, therefore, focus our discussion below on candidate genetic changes identified in studies on other drosophilid herbivore and specialist lineages, which provide compelling evidence for their role in these dietary shifts.

Despite consistent reductions in gene family sizes across all 3 herbivorous species (Fig. 2b, Supplementary Table 3 in Supplementary File 2), we did not find significant gene turnover, duplications, or losses for most chemosensory and detoxification gene families along the ancestral herbivore branch (Fig. 2c–e), contrary to the long-held view that the transition to herbivory involves extensive expansions and contractions of these gene families (McBride 2007; Edger et al. 2015; Johnson et al. 2018). The only exceptions were GRs and OBPs which were lost at a significantly higher rate (Fig. 2e). The lack of excessive copy number changes among the remaining gene families suggests that the initial transition to herbivory requires a more limited set of changes. This implies that novel key innovations related to sensing or detoxifying plant toxins may not be necessary for the evolution of herbivory, contrary to findings in other herbivore lineages (Berenbaum et al. 1996; Wheat et al. 2007) (although other innovations, like the plant-penetrating ovipositor, may be important (Peláez et al. 2022)). Instead in some herbivore lineages, like within *Scaptomyza*, the evolution of herbivorous feeding may have depended on pre-adaptation/exaptation. Nonherbivores, including microbe-feeding drosophilids, may have already evolved adaptations to find plants and deal with plant toxins, possibly from using dead or dying plant substrates ancestrally. This contrasts with individual herbivore lineages leading to extant *Scaptomyza* species that experienced high gene turnover rates within most of the gene families surveyed. Specialization or host plant switching—processes that play a critical role in driving herbivore diversification rates (Janz et al. 2006; Hardy and Otto 2014)—instead may have been responsible for these dramatic genomic changes.

The heavy loss of GRs and OBPs is consistent with the neural limitation hypothesis (Bernays 2001). This hypothesis posits that the loss of chemosensory genes, by limiting sensory inputs, facilitates rapid and accurate decision-making in herbivores that are faced with many host plant options. While our phylogenetic analyses could not elucidate precisely whether the identified genetic changes along the ancestral branch occurred before, during, or after herbivory evolved, several of the candidate genes have been implicated in other dietary transitions (discussed below), providing strong support for their involvement in the evolution of herbivory as well.

### Evolutionary patterns across GRs involved in detecting bitter compounds

Numerous studies have now shown that expansions of bitter GRs are typical of generalist arthropod species, to enable the detection of a wide variety of plant-derived bitter compounds, while oligophagous and monophagous herbivores derived from generalist ancestors tend to lose some of these bitter GRs. This pattern has been found in butterflies, moths, aphids, flies, beetles, sawflies, and mites (Smadja et al. 2009; Xu et al. 2016; Suzuki et al. 2018; Crava et al. 2020; Vertacnik et al. 2021). Our results suggest that the ancestral herbivore lineage experienced a loss in bitter detection because of losing many bitter GRs (paralogs of *Gr39aA*, *Gr59a, Gr59d*) and experiencing rapid evolution of other bitter GRs (*Gr98a* and *Gr98bcd*). These evolutionary genetic changes likely reduced the ability of these flies to detect bitter compounds, which would otherwise limit the intake of their host plants. A reduction in bitter sensitivity was found in *D. suzukii* (Dweck et al. 2021), which evolved herbivory (feeding on living, ripe fruit) on a similar timescale as herbivorous *Scaptomyza* species (Suvorov et al. 2022). However, although *D. suzukii* is herbivorous, it is polyphagous in many plant families (Poyet et al. 2015), suggesting that reduction in bitter reception is a trait involved in the transition to herbivory, possibly independent of specialization. It is still not known to what extent bitter sensitivity has been reduced in herbivorous Scaptomyza specifically by these copy number and protein-coding changes, or whether transcriptional down-regulation of bitter GRs also plays a significant role, as it does in *D. suzukii* (Dweck et al. 2021).

With the exception of *Gr39aA*, these candidate bitter GRs do not encode “commonly expressed receptors”—a set of bitter GRs expressed in all bitter gustatory neurons (Weiss et al. 2011; Dweck and Carlson 2020). This suggests that, despite a diet containing bitter, toxic compounds, *Scaptomyza* species are still able to detect bitter compounds generally, which is also true of specialist feeders on toxic hosts: *D. sechellia*, *D. erecta,* and *D. suzukii* (Dweck and Carlson 2020; Dweck et al. 2021). Bitter detection thus likely continues to be important for herbivores to differentiate toxin levels between individual leaves or host plants and distinguish old from young or healthy from damaged plants. Certainly, *S. flava* develops more slowly feeding on plants bearing aliphatic and indolic glucosinolates (Gloss et al. 2019), so differentiating between different bitter host-derived compounds could impact their fitness.

The lost copies of *Gr39a* (tandem duplicates of the isoform A-specific exon)*, Gr59a*, and *Gr59d* are particularly interesting candidates to focus on for future study in relation to the evolution of toxin specialization, considering all 3 GRs have been implicated in dietary shifts in other species. *Gr59a* and *Gr59d* both underwent expansions in *D. suzukii* (Hickner et al. 2016), while both of these genes were lost in *D. sechellia* and *D. erecta* (McBride et al. 2007). The pattern of expansion of *Gr59a* and *Gr59d* in a generalist (*D. suzukii*) and contraction in specialists (*D. sechellia*, and *D. erecta*) suggests their involvement in mediating host breadth. Signals of positive selection have also been detected in *Gr59a* in *D. yakuba mayottensis,* which convergently evolved specialization on noni fruit along with *D. sechellia* (Ferreira et al. 2020). Similarly, losses of *Gr39aA* have occurred across both *D. sechellia* and *D. erecta* (McBride et al. 2007). *Gr39a* encodes several different isoforms, but only *Gr39aA* is expressed in all bitter gustatory neurons and is involved in the detection of many bitter compounds in *D. melanogaster* (Dweck and Carlson 2020). Because *D. melanogaster* only bears a single-copy of the A exon, it remains unknown whether these additional *Gr39aA* copies are expressed in species bearing multiple copies.

Notably, all 3 of these GRs (*Gr39aA, Gr59a*, and *Gr59d*) exhibit numerous tandem duplications. The remarkably high rate of turnover for *Gr59d* and *Gr39aA* across the sampled species (Supplementary Table 6 in Supplementary File 2) suggests that there may be transposable elements in the vicinity driving these duplications, which could be selected upon during dietary shifts to generate a dosage effect, where more copies may enable stronger bitter detection.

### Concerted losses of and positive selection on genes involved in olfaction

Although we did not find excessively high levels of OR gene turnover at the base of the *Scaptomyza* herbivore clade, the ORs that were lost shared related and ecologically important functions, specifically in detecting yeast and fruit volatiles. Goldman-Huertas et al. (2015) showed that the evolution of herbivory in *S. flava* was associated with OR gene losses that reduced attraction toward their ancestral diet of yeast feeding. This was mediated through a behavioral loss of attraction to yeast volatiles, putatively ancestral feeding attractants, and with the stepwise loss of olfactory receptors (*Or22a*, *Or42b*, and *Or85d*) tuned to detect them (Goldman-Huertas et al. 2015). With full annotations from 4 other *Scaptomyza* species, we obtained results consistent with this finding, with the addition of 2 other yeast volatile-detecting ORs (*Or42a* and *Or85aLike*) that experienced relaxed purifying selection (Table 1, Supplementary Fig. 5 in Supplementary File 3). The loss of *Or22a*—which was the only OR loss shared exclusively by all herbivores in this study—strongly indicates an outsized role for *Or22a* in association with dietary shifts in conjunction with past studies on *Or22a* in various species. *Or22a* and *Or22b* (sometimes found as a single-copy in some lineages of *Drosophila*) have been subjected to repeated, independent bouts of natural selection, resulting in increased sensitivity in *D. sechellia* to esters specific to noni fruit (*Morinda citrifolia*) (Dekker et al. 2006); increased sensitivity to *Pandanus* fruit volatiles in *D. erecta (*Linz et al. 2013); attraction to marula fruit (*Sclerocarya birrea*) in some populations of *D. melanogaster* (Aguadé 2009; Mansourian et al. 2018); and finally, loss of sensitivity toward fermentation odors and increased sensitivity to certain leaf volatiles in the ripe fruit specialist *D. suzukii* (Keesey et al. 2015). Collectively, this indicates the role of *Or22a* in driving attraction to fermenting plant tissues in drosophilids, which is no longer a niche used by ovipositing females of herbivorous species.

Interestingly, we also found a stronger selection on *Or56a* and *Or19a*. *Or56a* is a narrowly tuned OR in *Drosophila* to detect geosmin, an oviposition repellent produced by harmful microbes in rotting fruit (Stensmyr et al. 2012). Although we do not know whether *Or56a* still detects geosmin in *Scaptomyza*, it is possible that because herbivores require living, fresh plants for their larvae, geosmin-avoidance for oviposition may be even stronger. As for *Or19a*, which has been shown to be involved in the detection of citrus volatiles, specifically terpenes (Dweck et al. 2013), we suspect that this receptor in herbivores is under strong purifying selection because of its role in detecting other plant terpenes. Terpenes are one of the largest and most structurally diverse groups of herbivore-induced plant volatiles, and it is possible that the need to detect these compounds has resulted in a stronger purifying selection on *Or19a* in herbivorous *Scaptomyza*.

Previous divergence-based genomic analyses that included only *S. flava* and the 12 original *Drosophila* species with genome annotations found evidence of positive selection on *Or63a, Or67b* paralogs, *Or88a*, and *Or98a* (Goldman-Huertas et al. 2015), whereas here, we only found evidence of positive selection on *Or85f* and *OrN2.3prime* along the ancestral herbivore branch. This highlights the advantage of including additional closely related herbivorous species and nonherbivorous *Scaptomyza* species (i.e. *S. pallida* is ∼22 million years diverged from the herbivores vs *D. grimshawi* at *∼*36 million years (Peláez et al. 2022)). In particular, there is now strong evidence that the triplication and strong positive selection on *Or67b* paralogs are specifically related to *S. flava*'s specialization on plants of the order Brassicales, as these ORs are specifically tuned to the volatile isothiocyanates (Matsunaga et al. 2022).

The most striking herbivore-specific loss of genes occurred within the OBPs, which have been classified into 3 groups: classic OBPs that have 6 cysteine residues, minus-C with less than 6, and plus-C with more than 6 (Zhou et al. 2006). The majority of OBPs genes lost in herbivores (6/8) were among the plus-C class (Supplementary Fig. 13 in Supplementary File 3). The role of these lost plus-C OBPs is unclear, but some (*Obp58b, Obp58c,* and *Or85a*) are expressed only in the antennae and/or head of *D. melanogaster*, while others have been found additionally in the legs or body of adults (*Obp46a, Obp50c*, and *Obp93a*) (Zhou et al. 2004; Larter et al. 2016). We speculate that the reason for the excess loss of plus-C OBPs may be because they are more vulnerable to damage by plant toxins, like the Brassicales-specific isothiocyanates, which are highly electrophilic and attack nucleophilic sulfhydryl moieties of the cysteine residues (Kawakishi and Kaneko 1987).

### Diverse functions of sensory genes associated with herbivory

In this study, we hypothesized that many genetic changes associated with the evolution of herbivory would involve genes that interact with host plant compounds. It was thus surprising to find significant changes in genes involved in detecting various other stimuli: carbon dioxide (relaxed purifying selection on *Gr63a*), salinity (positive selection on *Ppk19*), water/low osmolarity (stronger purifying selection on *Ppk28*), cooling (relaxed purifying selection on *Ir21a*), and pheromones (loss of *Gr68a,* strong purifying selection on *Ppk25*) (Bray and Amrein 2003; Liu et al. 2003; Kwon et al. 2007; Cameron et al. 2010; Ni et al. 2016). While dietary toxins play a significant role in driving the evolution of these gene families, it remains to be explored whether the leaf-mining lifestyle imposes other sensory changes. The leaf-mining larvae navigate through the leaf mesophyll, a fluid-filled cavity, where salt levels, temperature gradients, and risk of carbon dioxide poisoning may be significantly different than within decaying organic material. Additional studies will be needed to determine whether herbivorous *Scaptomyza* show altered responses to these stimuli and whether it is related to the leaf-mining/herbivorous lifestyle.

### The role of gene duplications in evolving plant detoxification mechanisms

Numerous studies have indicated that gene duplications, followed by functional divergence, can spur biological novelties—new traits or adaptations to new niches (reviewed in Carscadden et al. (2023)). Examples abound across the diversity of life and across complex traits, from trichromatic vision in old-world monkeys to snake venom phospholipase genes (Dulai et al. 1999; Lynch 2007). Contrary to our initial hypotheses and predictions that we might find numerous instances of gene duplications across chemosensory and detoxification gene families, our phylogenetic gene trees of each family revealed that duplications were more prevalent within individual herbivore species or among just the mustard specialists, with only 2 gene duplications (*GstS1* and *Ir67a*) shared by the 3 surveyed herbivores (the function of the latter is unknown in any species). This suggests that gene duplications may spur further specialization but may not play a prominent role in initial transitions to herbivory. There are many highly specialized herbivores that have evolved adaptations to plant secondary metabolites through duplicated detoxification genes (Wen et al. 2006; Fischer et al. 2008; Sehlmeyer et al. 2010). But phenotypic plasticity and regulatory evolution driving changes in the expression of existing detoxification genes have also been found in other herbivores. For instance, the lepidopteran herbivore *Trichoplusia ni* (cabbage looper) responds to defenses from different plant families through the induction of different detoxification genes in their midgut (Herde and Howe 2014), while the two-spotted spider mite *Tetranychus urticae* has evolved trans-acting upregulation of some CYPs allowing it to feed on 250 plant families (Kurlovs et al. 2022).

Further investigation into the role of the duplication and rapid evolution of *GstS1* (Table 1) may clarify its possible involvement in enabling herbivory within the *Scaptomyza* lineage. Its *D. melanogaster* ortholog encodes an enzyme involved in detoxifying oxidation products, lipid peroxidation products, and organometallic compounds, with expression in the adult central nervous system, as well as in-flight muscle (Clayton et al. 1998; Singh et al. 2001; Whitworth et al. 2005; Saisawang et al. 2012; Vorojeikina et al. 2017). *S. flava* larvae express both *GstS1* copies, but isothiocyanates, the defense compounds in mustard plants, do not induce strong up-regulation of either copy, reinforcing that this duplication is not specific to mustard feeding. Functional genetic testing in herbivorous drosophilids should reveal the fate and possible functional divergence of these 2 paralogs, specifically the specificity of each paralog toward diet-derived xenobiotics.

## Conclusions

Genomic comparisons of older herbivorous lineages to distantly related nonherbivores, or across herbivorous lineages, have uncovered striking expansions and losses of genes involved in chemosensation and detoxification in arthropods (e.g. Goldman-Huertas et al. 2015; Rane et al. 2016; Calla et al. 2017; Johnson et al. 2018; Schoville et al. 2018). Yet, the lack of dense sampling among closely related taxa has, in many cases, precluded pinpointing the timing of these changes relative to the evolution of herbivory vs the evolution of host plant specificity. Here, using a comparative genomic approach across a growing number of drosophilid genome assemblies, particularly herbivorous and nonherbivorous *Scaptomyza*, we found accelerated protein evolution and gene losses among some chemosensory genes that may be linked to the evolution of herbivory. If confirmed through further testing, the patterns of evolution we identified would lend support to the hypothesis that the chemical composition of plant tissues drives herbivore genome evolution, an idea at the core of early theories on species interactions that motivated the development of co-evolutionary theory (Fraenkel 1959; Ehrlich and Raven 1964; Swain 1977; Berenbaum and Zangerl 2008). Similar comparative approaches in other young herbivore lineages may reveal the extent to which the genomic changes tied to herbivory in *Scaptomyza* species reflect general strategies underpinning the evolution of herbivory.

## Supplementary Material

jkad133_Supplementary_Data

## Data Availability

The *S. flava* Illumina-only genome assembly (sfla_v1) is available as GenBank assembly accession GCA_003952975.1, and the PacBio/HiC/Illumina assembly (sfla_v2) has been deposited at GenBank under the accession JARNME000000000. Source data, scripts, and analysis output files are accessible as Supplementary Dataset 1–4 in Supplementary File 1 in the Dryad repository (doi:https://doi.org/10.6078/D14D8P); for further details, see “List of Supporting Datasets” in the Supplementary Materials in Supplementary File 1. Supplemental material available at G3 online.
