# Correction to: Evolution of chemosensory and detoxification gene families across herbivorous Drosophilidae

**DOI:** 10.1093/g3journal/jkad261

**Published:** 2023-11-20

This is a correction to: Julianne N Peláez, Andrew D Gloss, Benjamin Goldman-Huertas, Bernard Kim, Richard T Lapoint, Giovani Pimentel-Solorio, Kirsten I Verster, Jessica M Aguilar, Anna C Nelson Dittrich, Malvika Singhal, Hiromu C Suzuki, Teruyuki Matsunaga, Ellie E Armstrong, Joseph L M Charboneau, Simon C Groen, David H Hembry, Christopher J Ochoa, Timothy K O’Connor, Stefan Prost, Sophie Zaaijer, Paul D Nabity, Jiarui Wang, Esteban Rodas, Irene Liang, Noah K Whiteman, Evolution of chemosensory and detoxification gene families across herbivorous Drosophilidae, *G3 Genes|Genomes|Genetics*, Volume 13, Issue 8, August 2023, jkad133, https://doi.org/10.1093/g3journal/jkad133

In the originally published version of this manuscript, there was an erroneous link published in the body of the article, within section **Data availability**: this should read: “https://doi.org/10.6078/D14D8P” instead of: “(doi:10.6078/D1841H)”. Also within supplementary data, **File_S1_-_Supplementary_Methods.docx**, under **List of supporting Datasets**: this should read: “Dryad repository (https://doi.org/10.6078/D14D8P).” instead of: “Dryad repository (doi:10.6078/D1841H).”

These emendations have been made in the article.

