# Supplementary material for: Evolution of chemosensory and detoxification gene families across herbivorous Drosophilidae
Source: G3 (Bethesda). 2023 Jun 15;13(8):jkad133. doi: 10.1093/g3journal/jkad133 (PMC10411586; doi:10.1093/g3journal/jkad133)

# **SUPPLEMENTARY FIGURES**

## **Figure S1. Benchmarking with core dipteran genes.** High levels of completeness are found in (a) the genome assemblies and (b) gene annotations used to curate the chemosensory and detoxification gene families, estimated using BUSCO v.5.4.2 (diptera_odb10, n=3285 genes).


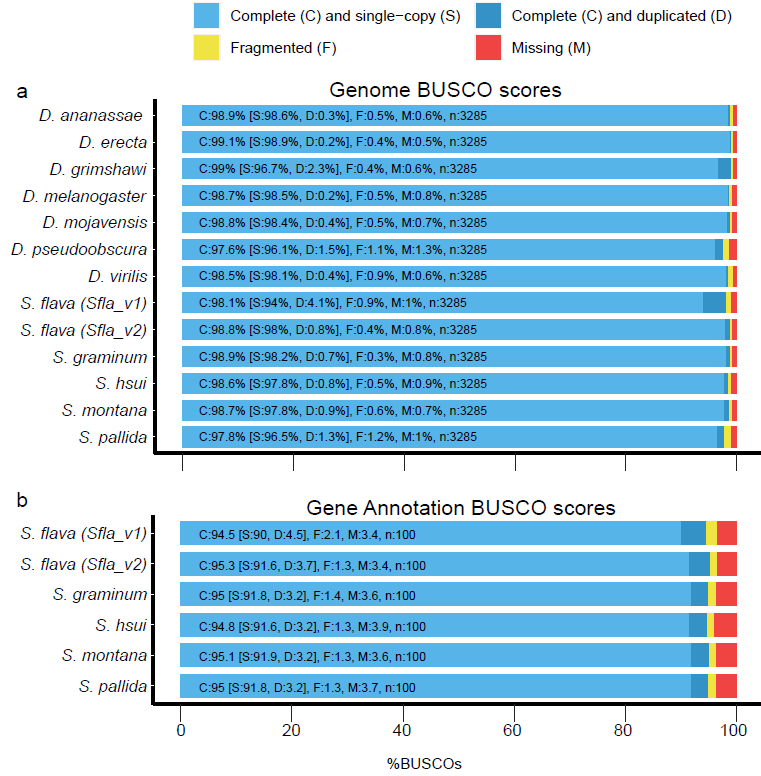


## **Figure S2. Phylogeny of gustatory receptors.** A midpoint rooted ML gene tree inferred and visualized using RAxML [(Stamatakis 2006)](https://paperpile.com/c/rx9jNZ/srvs) and iTOL [(Letunic and Bork 2021)](https://paperpile.com/c/rx9jNZ/CyJ9). Bootstrap support is given the size of squares at the midpoint of each branch, with only those >70 shown. Species are coded by font color: *D. grimshawi* (red), *S. pallida* (blue), *S. hsui* (magenta), *S. montana* (yellow), *S. flava* (green), *S. graminum* (purple). Gene orthology groups are indicated by alternating branch shading. Outer colored clade labels group genes by known functional classes: sweet/sugar receptors (red), CO_2_ receptors (turquoise), bitter receptors (green); thermosensitive receptor (purple), and pheromone receptor (orange).

**
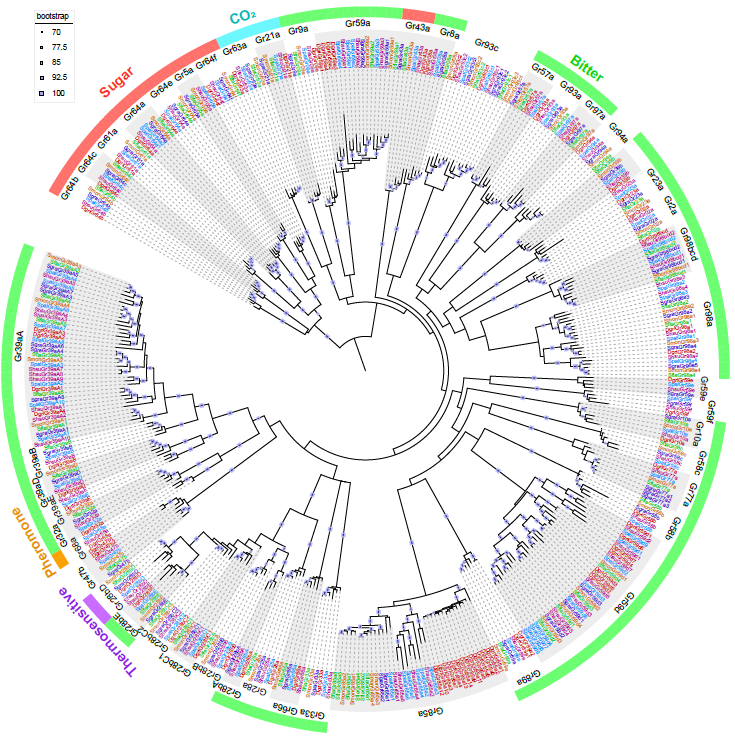
**

## **Figure S3. Phylogeny of ionotropic receptors.** A midpoint rooted ML gene tree inferred and visualized using RAxML [(Stamatakis 2006)](https://paperpile.com/c/rx9jNZ/srvs) and iTOL [(Letunic and Bork 2021)](https://paperpile.com/c/rx9jNZ/CyJ9). Bootstrap support is given the size of squares at the midpoint of each branch, with only those >70 shown. Species are coded by font and tip color: *D. grimshawi* (red), *S. pallida* (blue), *S. hsui* (magenta), *S. montana* (yellow), *S. flava* (green), *S. graminum* (purple). Gene orthology groups are indicated by alternating branch shading. Outer colored clade labels group genes by known IR classes: divergent IRs (blue), antennal IRs (yellow), IR co-receptors (orange).


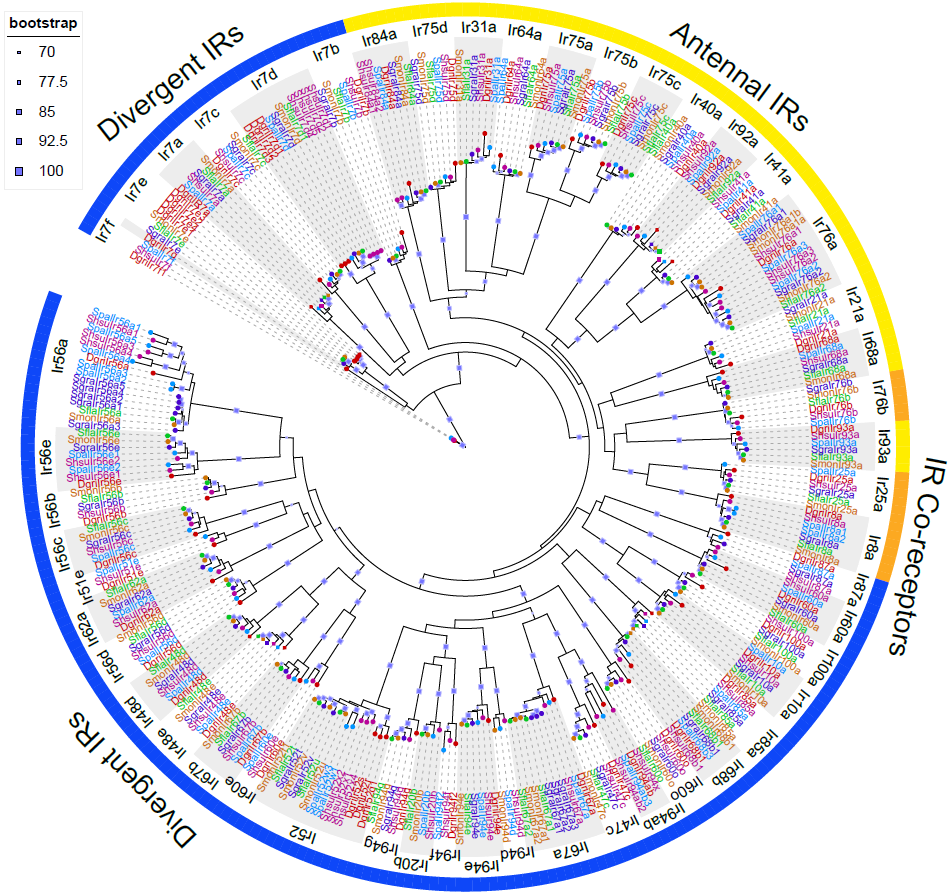


## **Figure S4. Phylogeny of odorant binding receptors.** A midpoint rooted ML gene tree inferred and visualized using RAxML [(Stamatakis 2006)](https://paperpile.com/c/rx9jNZ/srvs) and iTOL [(Letunic and Bork 2021)](https://paperpile.com/c/rx9jNZ/CyJ9). Bootstrap support is given the size of squares at the midpoint of each branch, with only those >70 shown. Species are coded by font and tip color: *D. grimshawi* (red), *S. pallida* (blue), *S. hsui* (magenta), *S. montana* (yellow), *S. flava* (green), *S. graminum* (purple). Gene orthology groups are indicated by alternating branch shading. Outer colored clade labels group genes by known OBP classes based on the number of conserved cysteine residues: classic (6 cysteines, green), plus-C (>6 cysteines, blue), minus-C (<6 cysteines, purple).


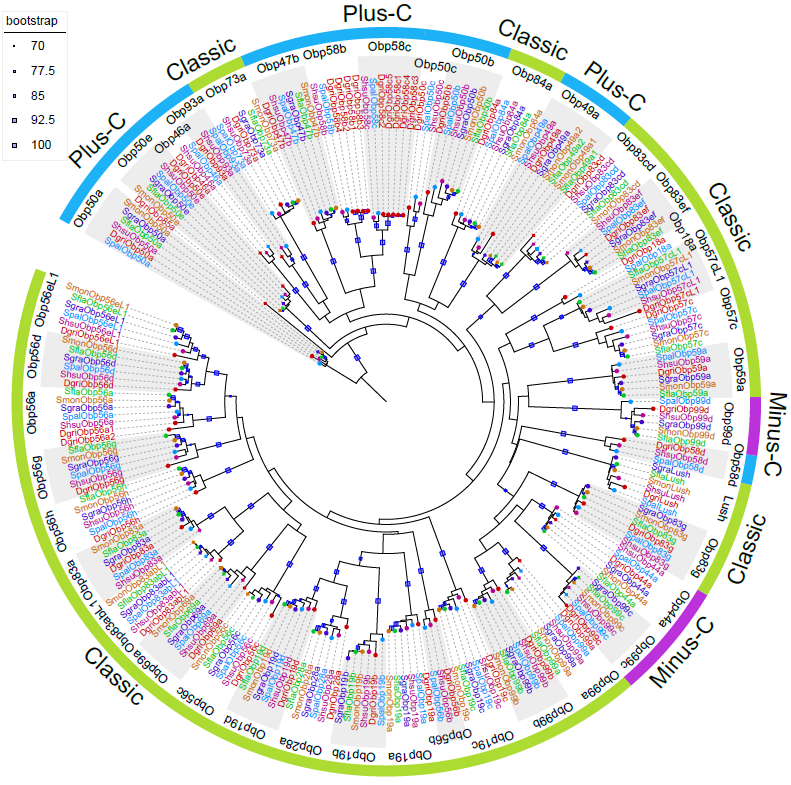


## **Figure S5. Phylogeny of olfactory receptors.** A midpoint rooted ML gene tree inferred and visualized using RAxML [(Stamatakis 2006)](https://paperpile.com/c/rx9jNZ/srvs) and iTOL [(Letunic and Bork 2021)](https://paperpile.com/c/rx9jNZ/CyJ9). Bootstrap support is given the size of squares at the midpoint of each branch, with only those >70 shown. Species are coded by font and tip color: *D. grimshawi* (red), *S. pallida* (blue), *S. hsui* (magenta), *S. montana* (yellow), *S. flava* (green), *S. graminum* (purple). Gene orthology groups are indicated by alternating branch shading.
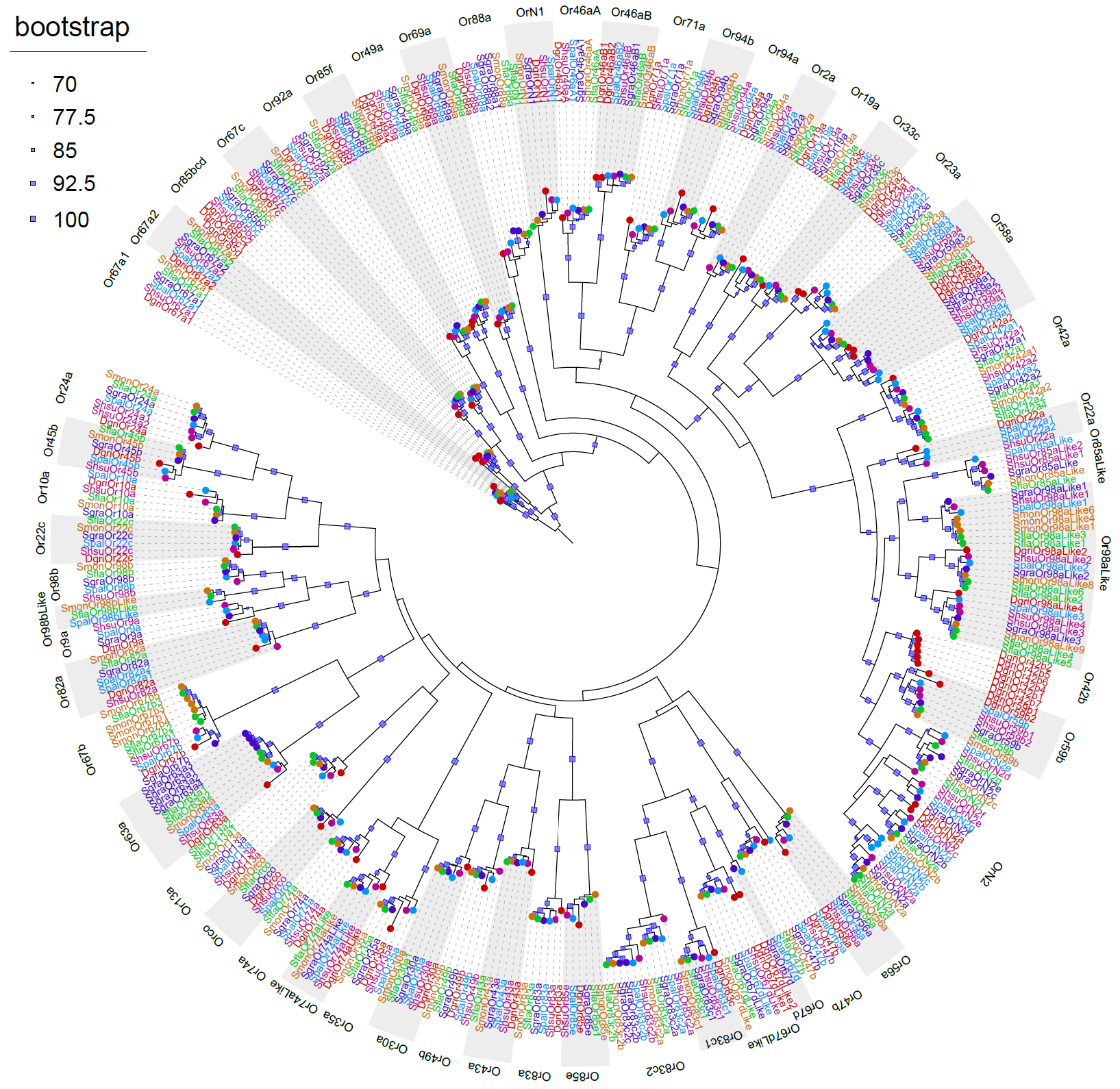


## **Figure S6. Phylogeny of degenerin/epithelial sodium channels.** A midpoint rooted ML gene tree inferred and visualized using RAxML [(Stamatakis 2006)](https://paperpile.com/c/rx9jNZ/srvs) and iTOL [(Letunic and Bork 2021)](https://paperpile.com/c/rx9jNZ/CyJ9). Bootstrap support is given the size of squares at the midpoint of each branch, with only those >70 shown. Species are coded by font and tip color: *D. grimshawi* (red), *S. pallida* (blue), *S. hsui* (magenta), *S. montana* (yellow), *S. flava* (green), *S. graminum* (purple). Gene orthology groups are indicated by alternating branch shading.


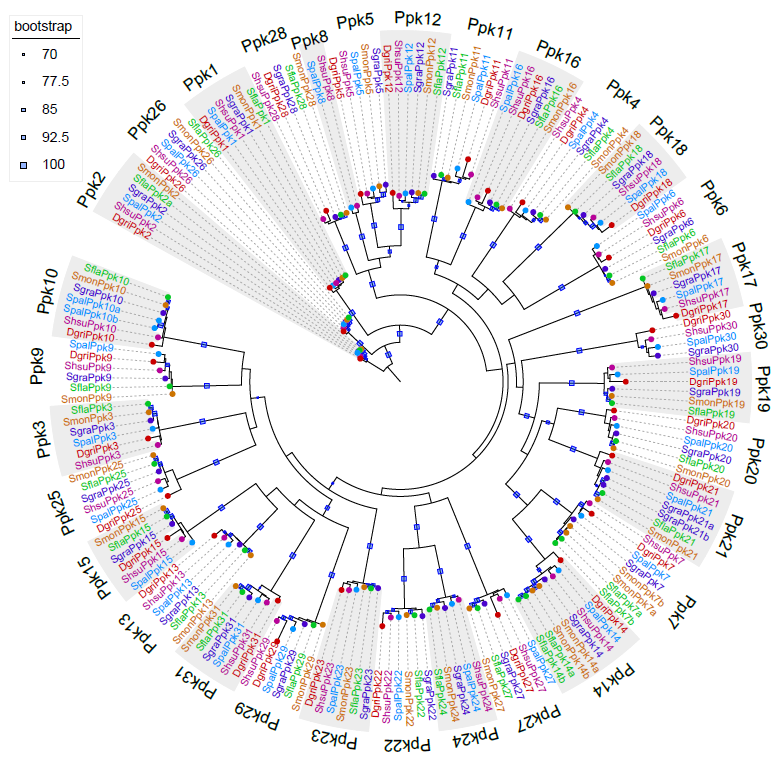


## **Figure S7. Phylogeny of transient receptor potential channels.** A midpoint rooted ML gene tree inferred and visualized using RAxML [(Stamatakis 2006)](https://paperpile.com/c/rx9jNZ/srvs) and iTOL [(Letunic and Bork 2021)](https://paperpile.com/c/rx9jNZ/CyJ9). Bootstrap support is given the size of squares at the midpoint of each branch, with only those >70 shown. Species are coded by font and tip color: *D. grimshawi* (red), *S. pallida* (blue), *S. hsui* (magenta), *S. montana* (yellow), *S. flava* (green), *S. graminum* (purple). Gene orthology groups are indicated by alternating branch shading.

**
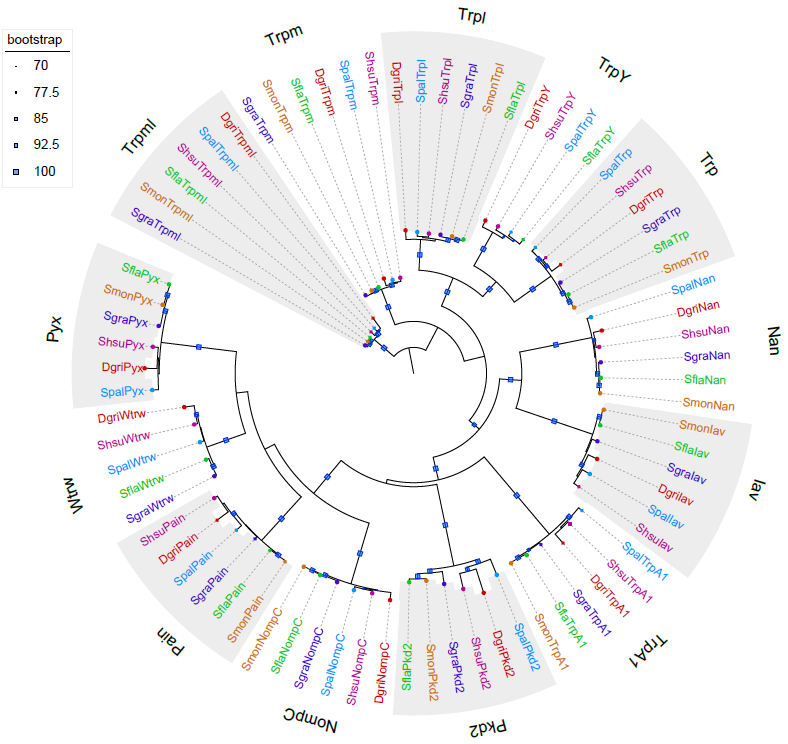
**

## **Figure S8. Phylogeny of glutathione S-transferases.** A midpoint rooted ML gene tree inferred and visualized using RAxML [(Stamatakis 2006)](https://paperpile.com/c/rx9jNZ/srvs) and iTOL [(Letunic and Bork 2021)](https://paperpile.com/c/rx9jNZ/CyJ9). Bootstrap support is given the size of squares at the midpoint of each branch, with only those >70 shown. Species are coded by font and tip color: *D. grimshawi* (red), *S. pallida* (blue), *S. hsui* (magenta), *S. montana* (yellow), *S. flava* (green), *S. graminum* (purple). Gene orthology groups are indicated by alternating branch shading. Outer colored clade labels group genes by known GST classes: microsomal (yellow), zeta (purple), omega (green), epsilon (orange), delta (red), sigma (magenta), and theta (blue).

**
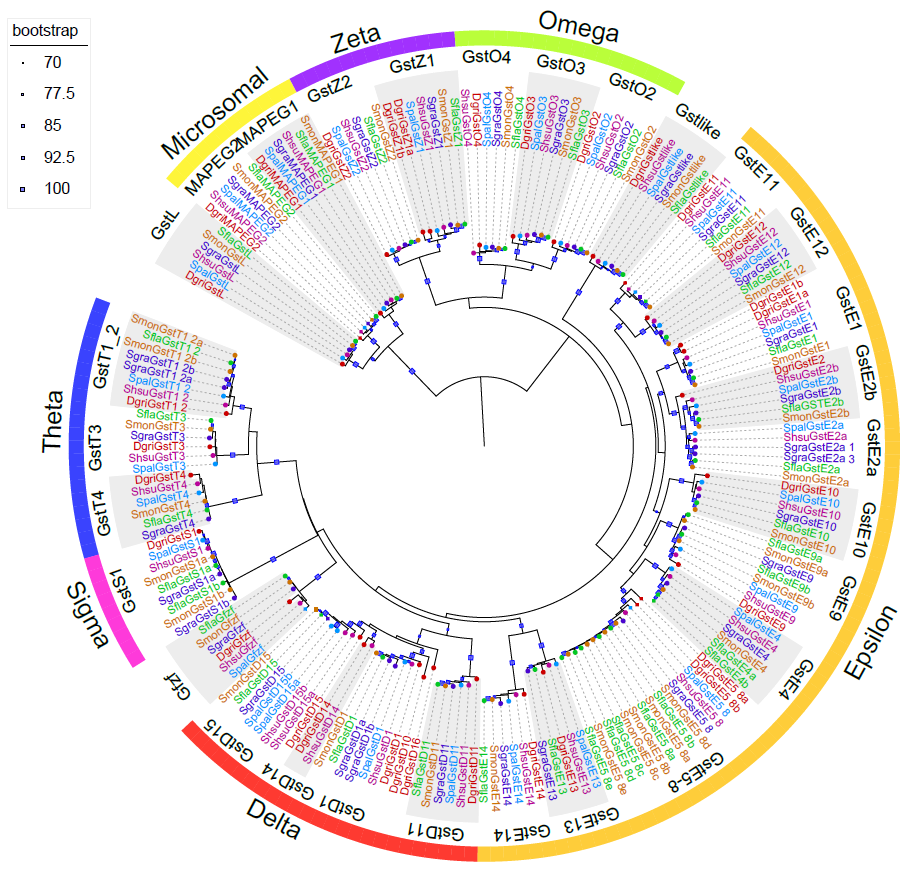
**

## **Figure S9. Phylogeny of cytochrome CYP450s.** A midpoint rooted ML gene tree inferred and visualized using RAxML [(Stamatakis 2006)](https://paperpile.com/c/rx9jNZ/srvs) and iTOL [(Letunic and Bork 2021)](https://paperpile.com/c/rx9jNZ/CyJ9). Bootstrap support is given the size of squares at the midpoint of each branch, with only those >70 shown. Species are coded by font and tip color: *D. grimshawi* (red), *S. pallida* (blue), *S. hsui* (magenta), *S. montana* (yellow), *S. flava* (green), *S. graminum* (purple). Gene orthology groups are indicated by alternating branch shading.


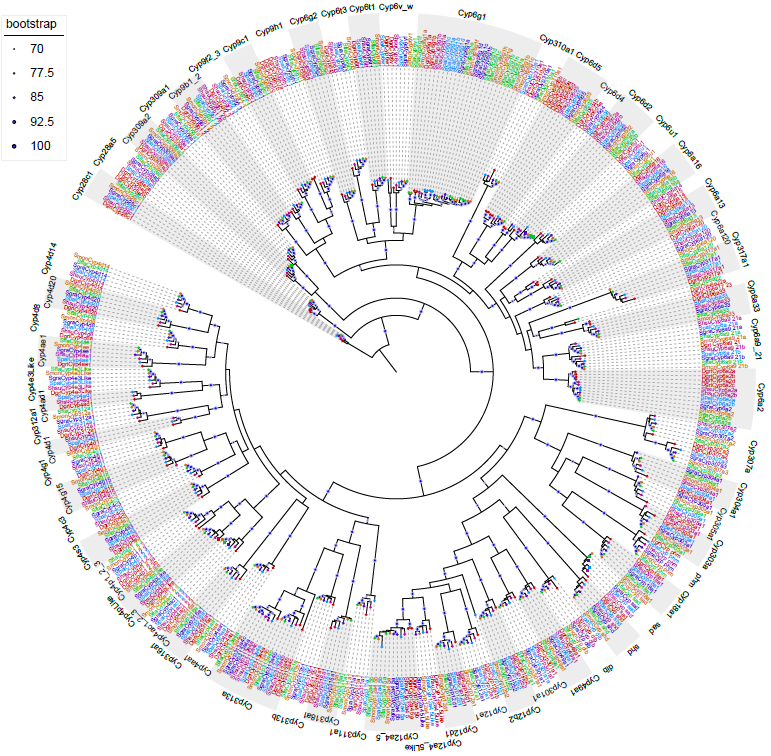


## **Figure S10. Phylogeny of UDP glucuronosyltransferases UGTs.** A midpoint rooted ML gene tree inferred and visualized using RAxML [(Stamatakis 2006)](https://paperpile.com/c/rx9jNZ/srvs) and iTOL [(Letunic and Bork 2021)](https://paperpile.com/c/rx9jNZ/CyJ9). Bootstrap support is given the size of squares at the midpoint of each branch, with only those >70 shown. Species are coded by font and tip color: *D. grimshawi* (red), *S. pallida* (blue), *S. hsui* (magenta), *S. montana* (yellow), *S. flava* (green), *S. graminum* (purple). Gene orthology groups are indicated by alternating branch shading.


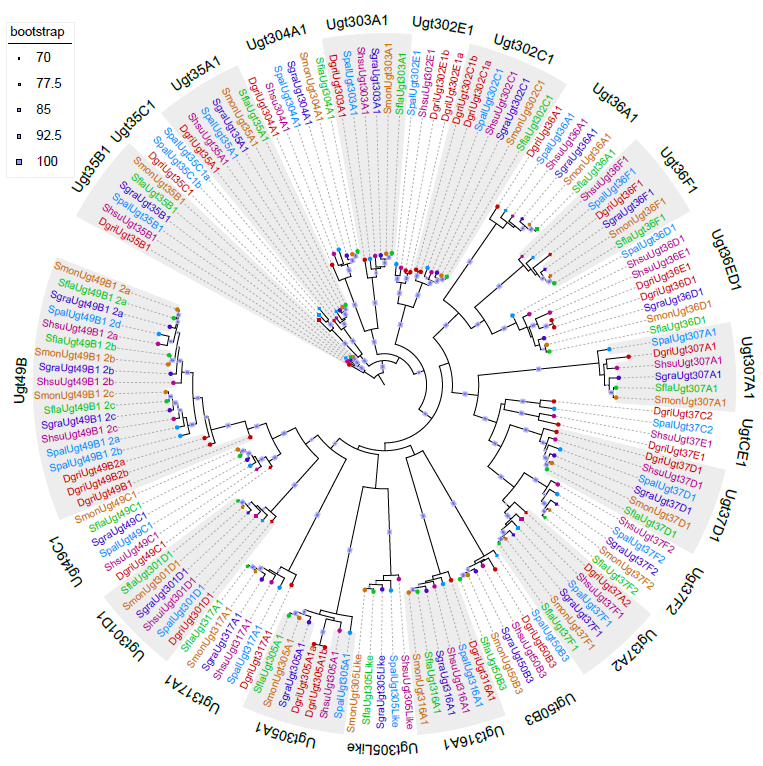


**Figure S11. Output branch labels for CAFE analysis.** Branch IDs correspond to those listed in Table S6. Herbivorous taxa in green font.


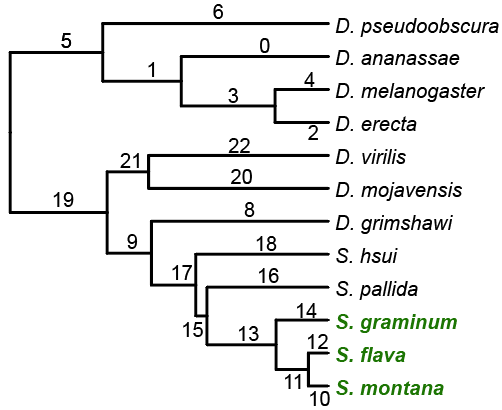


## **Figure S12. Higher rates of gene turnover among herbivorous *Scaptomyza* are not due to differences in branch lengths.** (a) Phylogeny [(Matsunaga *et al.* 2022)](https://paperpile.com/c/rx9jNZ/Yf34) highlighting branch lengths of *S. flava* and *S. graminum* versus *D. melanogaster* and *D. erecta*, the latter pair being comparable in branch lengths to the former pair. (b) CAFE results when the foreground was either the clade of *D. melanogaster* and *D. erecta* or the clade of *S. flava* and *S. graminum*. All chem = all chemosensory genes. all detox = all detoxification genes. Random = random set of 200 orthology groups.


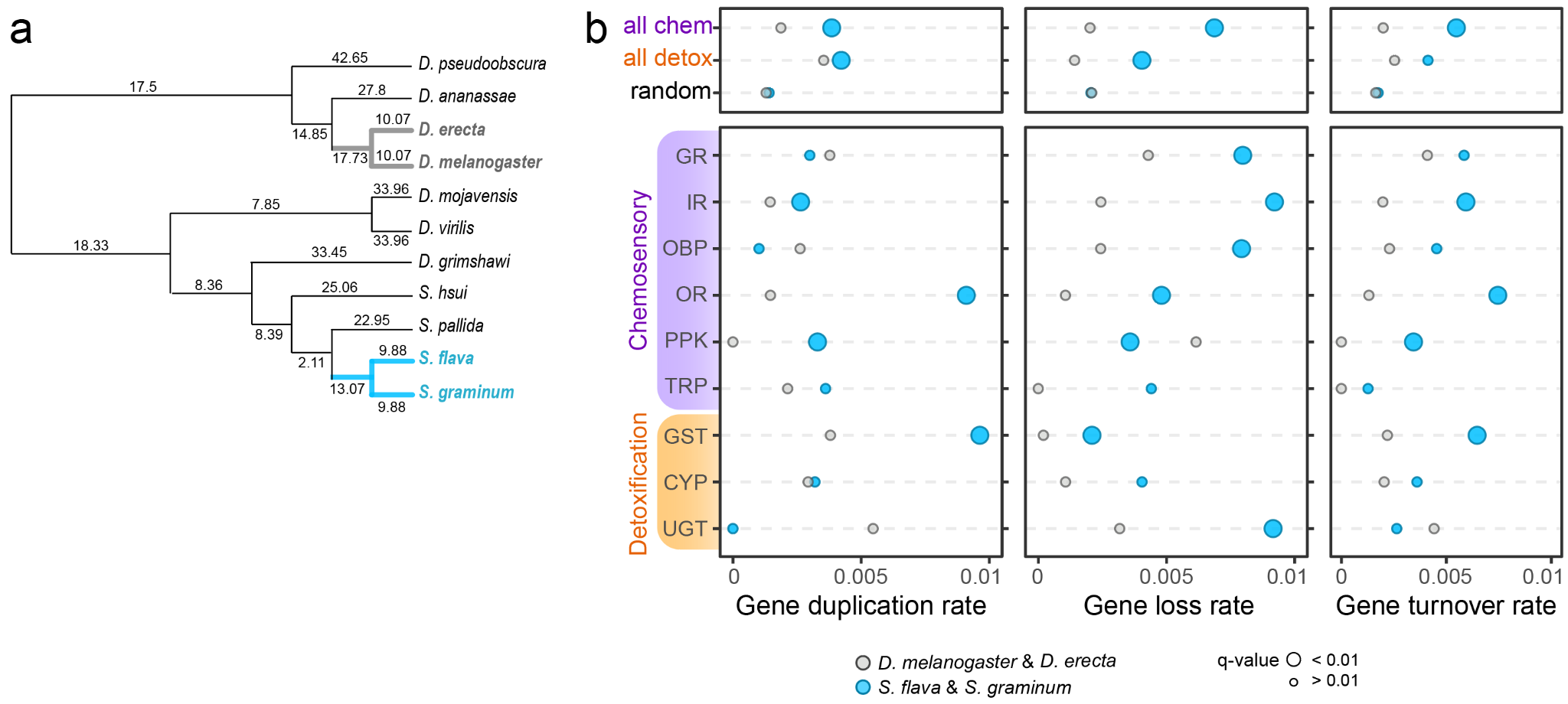


## **Figure S13. Dramatic loss of “Plus-C” odorant binding proteins at the base of herbivorous drosophilid clade**. (a) CAFE gene family expansion/contraction analysis for all OBPs – gains are shown above branches in blue, losses are shown below in red. Circles indicate feeding ecology: green = herbivorous, gray = non-herbivorous. (b) Gene counts by OBP class show that the majority of herbivore-specific losses are among the Plus-C OBPs. (c) Phylogeny of Plus-C OBPs. Thick branches indicate lineages with herbivore-specific losses. Node values indicate bootstrap support.


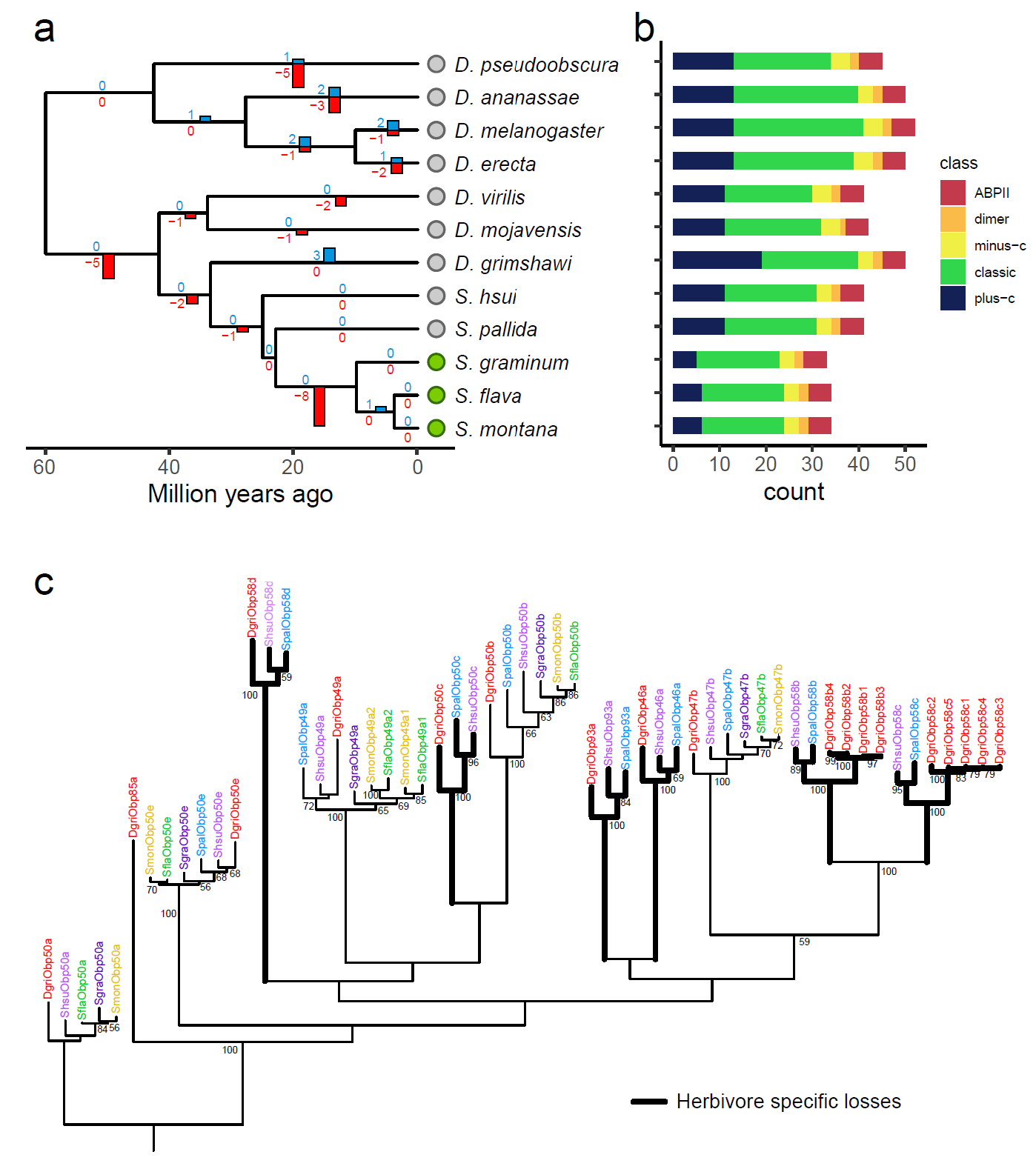


**Figure S14. Chemosensory genes duplicated, lost, or under changing selection regimes in the branch at the base of the herbivorous *Scaptomyza* lineage**. Localization map of chemosensory gene products in *D. melanogaster* adults and larvae. Only shown are those that have been lost in all herbivores (gray), duplicated (underlined), or experienced relaxed purifying selection (orange), stronger purifying selection (blue), or positive selection (red). Dorsal organ (DO); terminal organ (TO); dorsal, ventral, and posterior pharyngeal sense organ (DPS, VPS, PPS, respectively); labral sense organ (LSO); ventral cibarial sense organ (VCSO).


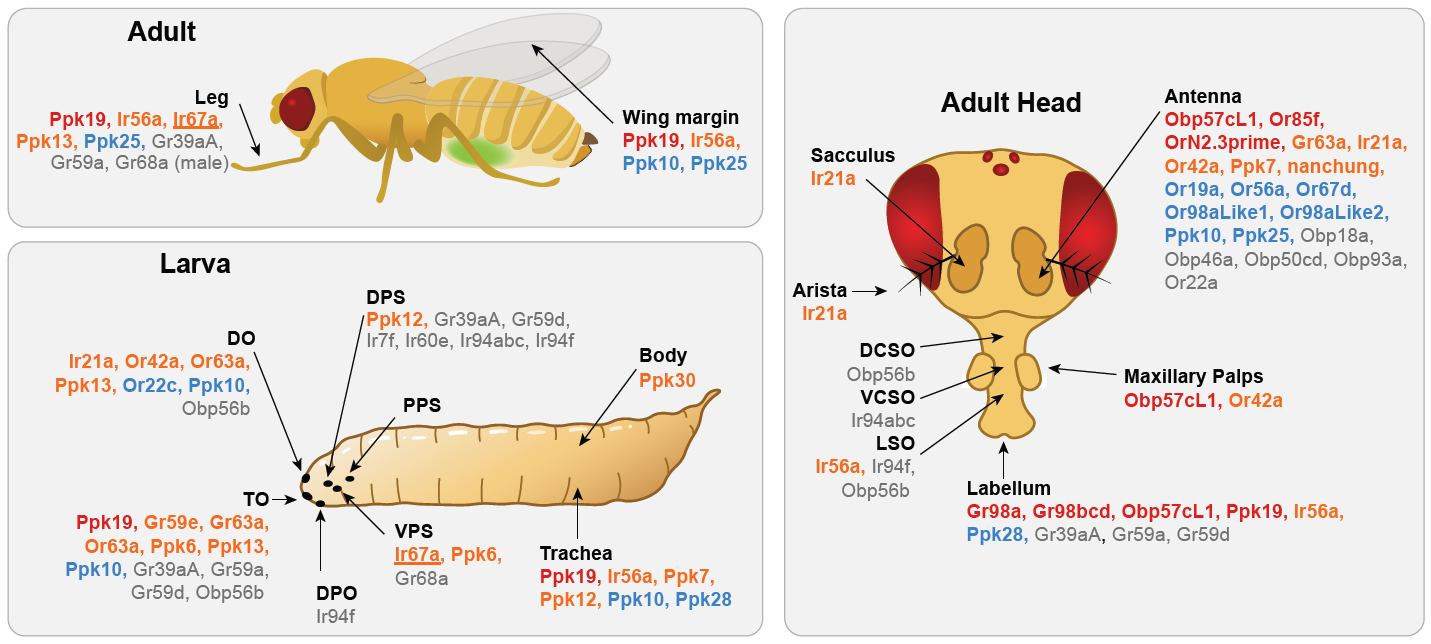

Supplement: jkad133_Supplementary_Data [file jkad133_supplementary_data.zip › File_S3_-_Supplementary_Figures_G3-2023-404324.docx]
