# Supplementary material for: Evolution of chemosensory and detoxification gene families across herbivorous Drosophilidae
Source: G3 (Bethesda). 2023 Jun 15;13(8):jkad133. doi: 10.1093/g3journal/jkad133 (PMC10411586; doi:10.1093/g3journal/jkad133)
Supplement: jkad133_Supplementary_Data [file jkad133_supplementary_data.zip › File_S1_-_Supplementary_Methods.docx]

# **SUPPLEMENTARY METHODS**

## ***S. flava* genome sequencing and assembly (sfla_v2).**

Dovetail HiC library preparation and sequencing. *S. flava* flies used for the genome assembly were collected from a partially inbred laboratory colony. The colony was founded from >150 larvae collected near Dover, NH, USA, and subsequently maintained for several years in the laboratory. 300 male flies were flash frozen in liquid nitrogen and stored at -80°C. A Dovetail HiC library was prepared in a similar manner as described previously [(Lieberman-Aiden *et al.* 2009)](https://paperpile.com/c/rx9jNZ/C5U9). Briefly, for each library, chromatin was fixed in place with formaldehyde in the nucleus and then extracted. Fixed chromatin was digested with DpnII, the 5’ overhangs filled in with biotinylated nucleotides, and free blunt ends were ligated. After ligation, crosslinks were reversed and the DNA purified from protein. Purified DNA was treated to remove biotin that was not internal to ligated fragments. DNA was then sheared to ~350 bp mean fragment size, and sequencing libraries were generated using NEBNext Ultra enzymes and Illumina-compatible adapters. Biotin-containing fragments were isolated using streptavidin beads before PCR enrichment of each library. The libraries were sequenced on an Illumina HiSeqX to produce 380 million 2x150 bp paired end reads, to approximately 30x sequence coverage.

PacBio library and sequencing. DNA samples were quantified using Qubit 2.0 Fluorometer (Life Technologies, Carlsbad, CA, USA). SMRTbell libraries (~20kb) for PacBio Sequel were constructed using SMRTbell Template Prep Kit 1.0 (PacBio, Menlo Park, CA, USA) using the manufacturer's recommended protocol. The pooled library was bound to polymerase using the Sequel Binding Kit 2.0 (PacBio) and loaded onto PacBio Sequel using the MagBead Kit V2 (PacBio). Sequencing was performed on two PacBio Sequel SMRT cells, using Instrument Control Software Version 5.0.0.6235, Primary analysis software Version 5.0.0.6236 and SMRT Link Version 5.0.0.6792.

Illumina library and sequencing. Illumina sequence data was generated from the “OHI-9 line” of *S. flava*, derived from a laboratory population initially collected in Belmont, MA, USA in 2008 that was inbred through 10 generations of single pair sibling mating on *A. thaliana* Col-0 plants. Paired-end 180 bp and 300 bp insert libraries and 3 kbp and 5 kbp mate pair libraries from OHI-9 female flies were sequenced with 100 bp read length on an Illumina Hiseq 2000 at the University of Arizona. Reads were quality filtered and Illumina TruSeq3 adapters were removed using Trimmomatic v0.35 [(Bolger *et al.* 2014)](https://paperpile.com/c/rx9jNZ/elrSF) with the following parameters: “LEADING:10 TRAILING:10 SLIDINGWINDOW:4:15 MINLEN:99”.

Falcon and HiRise initial assembly. An initial draft assembly of sfla_v2 was generated by Dovetail using the following methods, but was not used because of a high rate of errors detected and an inflated genome size, which we suspected was caused by high heterozygosity in the assembly. We report these methods, however, because this assembly was used for scaffolding our final assembly (details below). The genome assembly was performed using the FALCON 1.8.8 pipeline from Pacific Bioscience. First, 70-fold whole-genome, single-molecule, real-time sequencing (SMRT) data of *S. flava* was used as input to the traditional FALCON pipeline using a length cut-off that correspond to 50x coverage of data during the initial error-correcting stage. This resulted in 0.511 million error corrected reads with an N50 read length equal to 19.6 kb. Second, the error-corrected reads were processed by the overlap portion of the FALCON pipeline. The aligned reads were assembled in the third stage of FALCON into 3,561 primary contigs containing 443.7 Mbp with an NG50 contig length of 543.7 kbp. Finally, the assembly was polished through PacBio’s Arrow algorithm from SMRT Link 5.0.1, using the original raw-reads. The input de novo assembly, and Dovetail HiC library reads were used as input data for HiRise, a software pipeline designed specifically for using proximity ligation data to scaffold genome assemblies [(Putnam *et al.* 2016)](https://paperpile.com/c/rx9jNZ/4HWW). Dovetail HiC library sequences were aligned to the draft input assembly using bwa (http://github.com/lh3/bwa). The separations of Dovetail HiC read pairs mapped within draft scaffolds were analyzed by HiRise to produce a likelihood model for genomic distance between read pairs, and the model was used to identify and break putative misjoins, to score prospective joins, and make joins above a threshold.

Reassembly. As mentioned above, while this initial de novo assembly exhibited high scaffold contiguity, we discovered that it unfortunately contained a significant number of frameshift mutations in coding sequences that limited our ability to correctly annotate the genome. A BUSCO (v5.2.2, [(Manni *et al.* 2021)](https://paperpile.com/c/rx9jNZ/cEShM)) analysis only showed 83.4% complete BUSCOs, notably lower than typical long-read drosophilid genome assemblies (e.g., Kim et al. 2021). A manual examination of preliminary annotation records showed that approximately 15% of randomly checked genes contained one or more deletions resulting in fragmented exons. We attempted to further polish the scaffolds with Illumina data but that resulted in only 97.4% complete BUSCOs and many of the manually checked gene annotation records contained indels.

We thus decided to reassemble the genome, using the same data described here, with the long-read hybrid assembly pipeline described in Kim et. al (2021), which has been shown to produce highly complete genome assemblies for drosophilid flies with very few coding sequence indels [(Kim *et al.* 2021)](https://paperpile.com/c/rx9jNZ/TLqel). Briefly, we generated an initial draft assembly with Flye 2.9 [(Kolmogorov *et al.* 2019)](https://paperpile.com/c/rx9jNZ/VRb7c), identified and removed duplicated haplotypes (haplotigs) with purge_dups [(Guan *et al.* 2020)](https://paperpile.com/c/rx9jNZ/0dHHn), polished the draft assembly using the PacBio reads with one round of Racon [(Vaser *et al.* 2017)](https://paperpile.com/c/rx9jNZ/a2sEH), then polished further using Illumina reads with one round of Pilon [(Walker *et al.* 2014)](https://paperpile.com/c/rx9jNZ/QCDlx), only fixing base-level errors. The fully polished assembly was scanned for contaminant sequences using NCBI BLAST [(Johnson *et al.* 2008)](https://paperpile.com/c/rx9jNZ/57KxO) and BlobTools [(Laetsch and Blaxter)](https://paperpile.com/c/rx9jNZ/6GDgZ). Repetitive sequences in the assembly were identified with RepeatModeler2 [(Flynn *et al.* 2020)](https://paperpile.com/c/rx9jNZ/oBY53). This produced a fairly contiguous 323.9 Mbp assembly contained in 1,430 contigs, with an N50 of 575,920 bp and L50 of 148. BUSCO completeness was significantly improved (98.8%) and manual spot checks of another preliminary annotation showed no indel issues.

Scaffolding. To fully integrate the long-range assembly information we had previously generated with HiC, we scaffolded the less contiguous but more accurate new assembly with the error-prone Dovetail version, reasoning that base-level errors in the previous version were unlikely to impact reference-based scaffolding. Specifically, we soft-masked both genomes using the repeat library generated in the previous step, using RepeatMasker [(Smit *et al.* 2013)](https://paperpile.com/c/rx9jNZ/gxnsa). Then, we created a whole-genome alignment with Progressive Cactus [(Armstrong *et al.* 2020)](https://paperpile.com/c/rx9jNZ/albTN) and used the RagOut reference-based scaffolder [(Kolmogorov *et al.* 2014)](https://paperpile.com/c/rx9jNZ/v4ozo) to scaffold the new genome, using the old assembly as the reference. The final genome assembly of *S. flava* (sfla_v2) was scaffolded into 1,252 scaffolds covering 315.4 Mbp (N50 = 32.966 Mb) with a maximum length of 85.98 Mbp and with 460 gaps.

Comparative annotation of *Scaptomyza* genomes. Gene annotations previously created for an Illumina-only *S. flava* assembly (sfla_v1) (described below) were transferred to the assemblies of *S. graminum, S. hsui, S. pallida, S. montana*, and this newer *S. flava* assembly, using whole-genome Progressive Cactus alignments and the Comparative Annotation Toolkit (CAT, [(Fiddes *et al.* 2018)](https://paperpile.com/c/rx9jNZ/fToLk)). A *D. grimshawi* genome [(Kim *et al.* 2021)](https://paperpile.com/c/rx9jNZ/TLqel) was used as an outgroup. All genomes were repeat-masked using repeat libraries generated with RepeatModeler2 and soft-masked using RepeatMasker. A published phylogeny for these species [(Suvorov *et al.* 2022)](https://paperpile.com/c/rx9jNZ/eLphr) was used as a guide tree for the alignment. RNA-seq data generated from female *S. flava* gut, proboscis, and maxillary palp organs was downsampled to 100X coverage using BBTools [(Bushnell 2014)](https://paperpile.com/c/rx9jNZ/lYpxV) and aligned to the *S. flava* genome using STAR [(Dobin *et al.* 2013)](https://paperpile.com/c/rx9jNZ/5pSiz) to assist CAT annotation. RNA-seq data are currently not available for any of the other species. Briefly, CAT uses the transMap mode to project annotations from a reference (i.e, the original *S. flava* genome) onto the target genomes, evaluating the projections and correcting them with Augustus [(Stanke *et al.* 2008)](https://paperpile.com/c/rx9jNZ/6wcis) and utilizing RNA-seq evidence if it exists.

***S. flava* Illumina-only assembly (sfla_v1).**

Prior to the generation of our main assembly sfla_v2, an *S. flava* assembly generated from Illumina sequencing was utilized by various projects on this species (e.g. [(Peláez *et al.* 2020)](https://paperpile.com/c/rx9jNZ/4XxgB), [(Gloss *et al.* 2022)](https://paperpile.com/c/rx9jNZ/wD7Jg), Verster et al. 2019). The sequence data was also used in polishing steps in the long read assembly, for generating automated annotations that were then also copied over to the newer assembly, sfla_v2, and for estimating repeat coverage.

Assembly and annotation. Read pairs that survived quality filtering were subsampled to an estimated ~90x coverage of the *S. flava* genome and assembled using ALLPATHS-LG [(Gnerre *et al.* 2011)](https://paperpile.com/c/rx9jNZ/TA2ZU) on the XSEDE high performance computing system. Contigs were extended and ambiguous regions were resolved iteratively using GapCloser [(Luo *et al.* 2012)](https://paperpile.com/c/rx9jNZ/j3urN). Prior to annotation, repeat regions were masked using RepeatMasker [(Smit *et al.* 2010)](https://paperpile.com/c/rx9jNZ/JKYV3) with the *Drosophila* repeat library. Protein-coding genes were annotated using MAKER2 [(Holt and Yandell 2011)](https://paperpile.com/c/rx9jNZ/XTvMb), with the *S. flava* transcriptome [(Whiteman *et al.* 2012)](https://paperpile.com/c/rx9jNZ/zNuhI) and predicted gene sequences from 12 *Drosophila* species (FlyBase release 2013_06) provided to inform gene models. We recovered 17,997 genes in our liberal gene set (annotations predicted by Augustus). The proportion of core dipteran genes recovered in our assembly was determined with BUSCO v5.4.2 [(Simão *et al.* 2015)](https://paperpile.com/c/rx9jNZ/zqymi).

Repetitive element content. Repeat identification was carried out using both homology-based and ab-initio approaches. We used the Drosophila RepBase repeat database for the homology-based annotation (http://www.girinst.org/repbase; update 20150807) within RepeatMasker version open-4.0.6 [(Smit *et al.* 2010)](https://paperpile.com/c/rx9jNZ/JKYV3). The RepeatMasker option -gccalc was used to infer GC content for each contig separately to improve the repeat annotation. Ab-initio repeat finding was carried out using RepeatModeler version 1.73 (http://repeatmasker.org/RepeatModeler.html).

## **Gene model validation.**

To validate lost genes of interest (GOIs), we performed an additional TBLASTN search for each GOI in *S. flava*, as well as genes assumed to be proximal to GOIs (PGOIs) based on their location in *D. grimshawi* or *D. virilis*. These searches used the predicted orthologs in *D. grimshawi* or *D. virilis* as queries. GOIs were considered truly absent if TBLASTN searches yielded no hits. In some cases, homology between conserved protein domains resulted in weakly supported hits to the *S. flava* genome and/or transcriptome, in which case the aligned region was extracted, translated, and BLASTed against the NCBI nr database. The gene was considered lost if the output showed stronger homology to genes other than the GOI. To avoid confirmation bias, we also did this with the *S. flava* PGOIs to ensure their identity matched the expected ortholog in *D. grimshawi*. The absence of the GOIs, coupled with the presence of 95% of the PGOIs strongly support that the GOIs are truly lost and are not an artifact of missing scaffolds in the genome assembly.

Finally, to guard against errors in our curation of the published *Drosophila* genomes, we compared our gene curations to those from published studies [(Low *et al.* 2007; Almeida *et al.* 2014; Good *et al.* 2014)](https://paperpile.com/c/rx9jNZ/ULR0r+HKJlE+2admd) and those inferred in OrthoDB [(Zdobnov *et al.* 2017)](https://paperpile.com/c/rx9jNZ/6GmpU). We performed comprehensive TBLASTN searches against the relevant genome assemblies to search for the full complement of orthologous genes, re-curated gene models if necessary, and manually inspected aligned gene models. In a few cases, we discarded genes that had high similarity (>99% nucleotide identity) and perfect synteny to another scaffold in the assembly because these are likely artifactual duplicates*.*

## **Population genomics.**

DNA pooled from 45 wild-collected *S. flava* larvae was sequenced to yield 100 bp paired-end reads on an Illumina HiSeq 2000. Methods on sample collection, sequencing, and read mapping can be found in Pelaez, Gloss et al. 2022. Nucleotide diversity (π) was calculated across four-fold degenerate sites using the script Variance-sliding.pl from Popoolation v.1.2.2 [(Kofler *et al.* 2011)](https://paperpile.com/c/rx9jNZ/tDcw9) for repeat-masked autosomal scaffolds (N = 819) greater than 20 kb in length. Parameters were: a ploidy level of 90, minimum allele count of two, minimum quality score of 20, minimum coverage of four, and a maximum coverage of 100. Scaffolds were considered autosomal if more than half the predicted proteins had a best BLASTP score to an autosome in *D. melanogaster*.

**References**

[Almeida, F. C., A. Sánchez-Gracia, J. L. Campos, and J. Rozas, 2014 Family Size Evolution in Drosophila Chemosensory Gene Families: A Comparative Analysis with a Critical Appraisal of Methods. Genome Biology and Evolution 6: 1669–1682.](http://paperpile.com/b/rx9jNZ/2admd)

[Alves, G., J. Sallé, S. Chaudy, S. Dupas, and G. Manière, 2014 High-NaCl perception in Drosophila melanogaster. J. Neurosci. 34: 10884–10891.](http://paperpile.com/b/rx9jNZ/Unopg)

[Armstrong, J., G. Hickey, M. Diekhans, I. T. Fiddes, A. M. Novak *et al.*, 2020 Progressive Cactus is a multiple-genome aligner for the thousand-genome era. Nature 587: 246–251.](http://paperpile.com/b/rx9jNZ/albTN)

[Aryal, B., and Y. Lee, 2022 Histamine avoidance through three gustatory receptors in Drosophila melanogaster. Insect Biochem. Mol. Biol. 144: 103760.](http://paperpile.com/b/rx9jNZ/guctm)

[Bolger, A. M., M. Lohse, and B. Usadel, 2014 Trimmomatic: a flexible trimmer for Illumina sequence data. Bioinformatics 30: 2114–2120.](http://paperpile.com/b/rx9jNZ/elrSF)

[Bray, S., and H. Amrein, 2003 A putative Drosophila pheromone receptor expressed in male-specific taste neurons is required for efficient courtship. Neuron 39: 1019–1029.](http://paperpile.com/b/rx9jNZ/ksoYG)

[Bushnell, B., 2014 BBTools software package.](http://paperpile.com/b/rx9jNZ/lYpxV)

[Chung, H., T. Sztal, S. Pasricha, M. Sridhar, P. Batterham *et al.*, 2009 Characterization of Drosophila melanogaster cytochrome P450 genes. Proc. Natl. Acad. Sci. U. S. A. 106: 5731–5736.](http://paperpile.com/b/rx9jNZ/fJLM1)

[Clayton, J. D., R. M. Cripps, J. C. Sparrow, and B. Bullard, 1998 Interaction of troponin-H and glutathione S-transferase-2 in the indirect flight muscles of Drosophila melanogaster. J. Muscle Res. Cell Motil. 19: 117–127.](http://paperpile.com/b/rx9jNZ/utcMz)

[Davies, L., D. R. Williams, I. A. Aguiar-Santana, J. Pedersen, P. C. Turner *et al.*, 2006 Expression and down-regulation of cytochrome P450 genes of the CYP4 family by ecdysteroid agonists in Spodoptera littoralis and Drosophila melanogaster. Insect Biochem. Mol. Biol. 36: 801–807.](http://paperpile.com/b/rx9jNZ/e1Yzr)

[Dobin, A., C. A. Davis, F. Schlesinger, J. Drenkow, C. Zaleski *et al.*, 2013 STAR: ultrafast universal RNA-seq aligner. Bioinformatics 29: 15–21.](http://paperpile.com/b/rx9jNZ/5pSiz)

[Dweck, H. K. M., and J. R. Carlson, 2020 Molecular Logic and Evolution of Bitter Taste in Drosophila. Curr. Biol. 30: 17–30.e3.](http://paperpile.com/b/rx9jNZ/eCWLK)

[Dworkin, I., and C. D. Jones, 2009 Genetic changes accompanying the evolution of host specialization in Drosophila sechellia. Genetics 181: 721–736.](http://paperpile.com/b/rx9jNZ/LXkp7)

[Ebrahim, S. A. M., H. K. M. Dweck, J. Stökl, J. E. Hofferberth, F. Trona *et al.*, 2015 Drosophila Avoids Parasitoids by Sensing Their Semiochemicals via a Dedicated Olfactory Circuit. PLoS Biol. 13: e1002318.](http://paperpile.com/b/rx9jNZ/NSOoW)

[Fiddes, I. T., J. Armstrong, M. Diekhans, S. Nachtweide, Z. N. Kronenberg *et al.*, 2018 Comparative Annotation Toolkit (CAT)—simultaneous clade and personal genome annotation. Genome Res. 28: 1029–1038.](http://paperpile.com/b/rx9jNZ/fToLk)

[Flynn, J. M., R. Hubley, C. Goubert, J. Rosen, A. G. Clark *et al.*, 2020 RepeatModeler2 for automated genomic discovery of transposable element families. Proc. Natl. Acad. Sci. U. S. A. 117: 9451–9457.](http://paperpile.com/b/rx9jNZ/oBY53)

[George, L. F., S. J. Pradhan, D. Mitchell, M. Josey, J. Casey *et al.*, 2019 Ion Channel Contributions to Wing Development in Drosophila melanogaster. G3 9: 999–1008.](http://paperpile.com/b/rx9jNZ/GeHmy)

[Gloss, A. D., A. Vergnol, T. C. Morton, P. J. Laurin, F. Roux *et al.*, 2022 Genome-wide association mapping within a local Arabidopsis thaliana population more fully reveals the genetic architecture for defensive metabolite diversity. Philos. Trans. R. Soc. Lond. B Biol. Sci. 377: 20200512.](http://paperpile.com/b/rx9jNZ/wD7Jg)

[Gnerre, S., I. Maccallum, D. Przybylski, F. J. Ribeiro, J. N. Burton *et al.*, 2011 High-quality draft assemblies of mammalian genomes from massively parallel sequence data. Proc. Natl. Acad. Sci. U. S. A. 108: 1513–1518.](http://paperpile.com/b/rx9jNZ/TA2ZU)

[Good, R. T., L. Gramzow, P. Battlay, T. Sztal, P. Batterham *et al.*, 2014 The molecular evolution of cytochrome P450 genes within and between drosophila species. Genome Biol. Evol. 6: 1118–1134.](http://paperpile.com/b/rx9jNZ/HKJlE)

[Guan, D., S. A. McCarthy, J. Wood, K. Howe, Y. Wang *et al.*, 2020 Identifying and removing haplotypic duplication in primary genome assemblies. Bioinformatics 36: 2896–2898.](http://paperpile.com/b/rx9jNZ/0dHHn)

[Holt, C., and M. Yandell, 2011 MAKER2: an annotation pipeline and genome-database management tool for second-generation genome projects. BMC Bioinformatics 12: 491.](http://paperpile.com/b/rx9jNZ/XTvMb)

[Johnson, M., I. Zaretskaya, Y. Raytselis, Y. Merezhuk, S. McGinnis *et al.*, 2008 NCBI BLAST: a better web interface. Nucleic Acids Res. 36: W5–9.](http://paperpile.com/b/rx9jNZ/57KxO)

[Kim, H., M. S. Choi, K. Kang, and J. Y. Kwon, 2016 Behavioral Analysis of Bitter Taste Perception in Drosophila Larvae. Chem. Senses 41: 85–94.](http://paperpile.com/b/rx9jNZ/2qYQY)

[Kim, B. Y., J. Wang, D. E. Miller, O. Barmina, E. K. Delaney *et al.*, 2021 Highly contiguous assemblies of 101 drosophilid genomes. Elife 10.:](http://paperpile.com/b/rx9jNZ/TLqel)

[Kofler, R., P. Orozco-terWengel, N. De Maio, R. V. Pandey, V. Nolte *et al.*, 2011 PoPoolation: a toolbox for population genetic analysis of next generation sequencing data from pooled individuals. PLoS One 6: e15925.](http://paperpile.com/b/rx9jNZ/tDcw9)

[Kolmogorov, M., B. Raney, B. Paten, and S. Pham, 2014 Ragout—a reference-assisted assembly tool for bacterial genomes. Bioinformatics 30: i302–i309.](http://paperpile.com/b/rx9jNZ/v4ozo)

[Kolmogorov, M., J. Yuan, Y. Lin, and P. A. Pevzner, 2019 Assembly of long, error-prone reads using repeat graphs. Nat. Biotechnol. 37: 540–546.](http://paperpile.com/b/rx9jNZ/VRb7c)

[Kwon, J. Y., A. Dahanukar, L. A. Weiss, and J. R. Carlson, 2007 The molecular basis of CO2 reception in Drosophila. Proc. Natl. Acad. Sci. U. S. A. 104: 3574–3578.](http://paperpile.com/b/rx9jNZ/wCOiA)

[Laetsch, and Blaxter BlobTools: Interrogation of genome assemblies [version 1; peer review: 2 approved with reservations]. F1000Res. 2017; 6: 1287. Publisher Full Text.](http://paperpile.com/b/rx9jNZ/6GDgZ)

[Letunic, I., and P. Bork, 2021 Interactive Tree Of Life (iTOL) v5: an online tool for phylogenetic tree display and annotation. Nucleic Acids Res. 49: W293–W296.](http://paperpile.com/b/rx9jNZ/CyJ9)

[Lieberman-Aiden, E., N. L. van Berkum, L. Williams, M. Imakaev, T. Ragoczy *et al.*, 2009 Comprehensive mapping of long-range interactions reveals folding principles of the human genome. Science 326: 289–293.](http://paperpile.com/b/rx9jNZ/C5U9)

[Liu, Y., M. Ge, T. Zhang, L. Chen, Y. Xing *et al.*, 2020 Exploring the correlation between deltamethrin stress and Keap1-Nrf2-ARE pathway from Drosophila melanogaster RNASeq data. Genomics 112: 1300–1308.](http://paperpile.com/b/rx9jNZ/SJEVs)

[Liu, L., A. S. Leonard, D. G. Motto, M. A. Feller, M. P. Price *et al.*, 2003 Contribution of Drosophila DEG/ENaC genes to salt taste. Neuron 39: 133–146.](http://paperpile.com/b/rx9jNZ/yuYdE)

[Low, W. Y., H. L. Ng, C. J. Morton, M. W. Parker, P. Batterham *et al.*, 2007 Molecular evolution of glutathione S-transferases in the genus Drosophila. Genetics 177: 1363–1375.](http://paperpile.com/b/rx9jNZ/ULR0r)

[Luo, R., B. Liu, Y. Xie, Z. Li, W. Huang *et al.*, 2012 SOAPdenovo2: an empirically improved memory-efficient short-read de novo assembler. Gigascience 1: 18.](http://paperpile.com/b/rx9jNZ/j3urN)

[Manni, M., M. R. Berkeley, M. Seppey, F. A. Simão, and E. M. Zdobnov, 2021 BUSCO Update: Novel and Streamlined Workflows along with Broader and Deeper Phylogenetic Coverage for Scoring of Eukaryotic, Prokaryotic, and Viral Genomes. Mol. Biol. Evol. 38: 4647–4654.](http://paperpile.com/b/rx9jNZ/cEShM)

[Matsunaga, T., C. E. Reisenman, B. Goldman-Huertas, P. Brand, K. Miao *et al.*, 2022 Evolution of Olfactory Receptors Tuned to Mustard Oils in Herbivorous Drosophilidae. Mol. Biol. Evol. 39.:](http://paperpile.com/b/rx9jNZ/Yf34)

[Peláez, J. N., A. D. Gloss, J. F. Ray, J. L. M. Charboneau, K. I. Verster *et al.*, 2020 Evolution and genetic basis of the plant-penetrating ovipositor, a key adaptation in herbivorous Drosophilidae. bioRxiv.](http://paperpile.com/b/rx9jNZ/4XxgB)

[Putnam, N. H., B. L. O’Connell, J. C. Stites, B. J. Rice, M. Blanchette *et al.*, 2016 Chromosome-scale shotgun assembly using an in vitro method for long-range linkage. Genome Res. 26: 342–350.](http://paperpile.com/b/rx9jNZ/4HWW)

[Rane, R. V., D. F. Clarke, S. L. Pearce, G. Zhang, A. A. Hoffmann *et al.*, 2019 Detoxification Genes Differ Between Cactus-, Fruit-, and Flower-Feeding Drosophila. J. Hered. 110: 80–91.](http://paperpile.com/b/rx9jNZ/BP1ps)

[Saisawang, C., J. Wongsantichon, and A. J. Ketterman, 2012 A preliminary characterization of the cytosolic glutathione transferase proteome from Drosophila melanogaster. Biochem. J 442: 181–190.](http://paperpile.com/b/rx9jNZ/H05Fk)

[Shim, J., Y. Lee, Y. T. Jeong, Y. Kim, M. G. Lee *et al.*, 2015 The full repertoire of Drosophila gustatory receptors for detecting an aversive compound. Nat. Commun. 6: 8867.](http://paperpile.com/b/rx9jNZ/LlrVB)

[Simão, F. A., R. M. Waterhouse, P. Ioannidis, E. V. Kriventseva, and E. M. Zdobnov, 2015 BUSCO: assessing genome assembly and annotation completeness with single-copy orthologs. Bioinformatics 31: 3210–3212.](http://paperpile.com/b/rx9jNZ/zqymi)

[Singh, S. P., J. A. Coronella, H. Benes, B. J. Cochrane, and P. Zimniak, 2001 Catalytic function of Drosophila melanogaster glutathione S-transferase DmGSTS1-1 (GST-2) in conjugation of lipid peroxidation end products. Eur. J. Biochem. 268: 2912–2923.](http://paperpile.com/b/rx9jNZ/FcFEx)

[Smit, A. F. A., R. Hubley, and P. Green, 2010 RepeatMasker Open-3.0.](http://paperpile.com/b/rx9jNZ/JKYV3)

[Smit, A. F. A., R. Hubley, and P. Green, 2013 RepeatMasker Open-4.0. Institute for Systems Biology.](http://paperpile.com/b/rx9jNZ/gxnsa)

[Stamatakis, A., 2006 RAxML-VI-HPC: maximum likelihood-based phylogenetic analyses with thousands of taxa and mixed models. Bioinformatics 22: 2688–2690.](http://paperpile.com/b/rx9jNZ/srvs)

[Stanke, M., M. Diekhans, R. Baertsch, and D. Haussler, 2008 Using native and syntenically mapped cDNA alignments to improve de novo gene finding. Bioinformatics 24: 637–644.](http://paperpile.com/b/rx9jNZ/6wcis)

[Stensmyr, M. C., E. Giordano, A. Balloi, A.-M. Angioy, and B. S. Hansson, 2003 Novel natural ligands for Drosophila olfactory receptor neurones. J. Exp. Biol. 206: 715–724.](http://paperpile.com/b/rx9jNZ/aNksT)

[Sung, H. Y., Y. T. Jeong, J. Y. Lim, H. Kim, S. M. Oh *et al.*, 2017 Heterogeneity in the Drosophila gustatory receptor complexes that detect aversive compounds. Nat. Commun. 8: 1484.](http://paperpile.com/b/rx9jNZ/nl31R)

[Sun, W., V. M. Margam, L. Sun, G. Buczkowski, G. W. Bennett *et al.*, 2006 Genome-wide analysis of phenobarbital-inducible genes in Drosophila melanogaster. Insect Mol. Biol. 15: 455–464.](http://paperpile.com/b/rx9jNZ/2vl7V)

[Suslak, T., 2015 There and back again: A stretch receptor’s tale [PhD]: The University of Edinburgh.](http://paperpile.com/b/rx9jNZ/bKaIi)

[Suvorov, A., B. Y. Kim, J. Wang, E. E. Armstrong, D. Peede *et al.*, 2022 Widespread introgression across a phylogeny of 155 Drosophila genomes. Curr. Biol. 32: 111–123.e5.](http://paperpile.com/b/rx9jNZ/eLphr)

[Swarup, S., T. I. Williams, and R. R. H. Anholt, 2011 Functional dissection of Odorant binding protein genes in Drosophila melanogaster. Genes Brain Behav. 10: 648–657.](http://paperpile.com/b/rx9jNZ/cvF9w)

[Trienens, M., K. Kraaijeveld, and B. Wertheim, 2017 Defensive repertoire of Drosophila larvae in response to toxic fungi. Mol. Ecol. 26: 5043–5057.](http://paperpile.com/b/rx9jNZ/hHezH)

[Vaser, R., I. Sović, N. Nagarajan, and M. Šikić, 2017 Fast and accurate de novo genome assembly from long uncorrected reads. Genome Res. 27: 737–746.](http://paperpile.com/b/rx9jNZ/a2sEH)

[Vorojeikina, D., K. Broberg, T. M. Love, P. W. Davidson, E. van Wijngaarden *et al.*, 2017 Editor’s Highlight: Glutathione S-Transferase Activity Moderates Methylmercury Toxicity During Development in Drosophila. Toxicol. Sci. 157: 211–221.](http://paperpile.com/b/rx9jNZ/T24o7)

[Walker, B. J., T. Abeel, T. Shea, M. Priest, A. Abouelliel *et al.*, 2014 Pilon: an integrated tool for comprehensive microbial variant detection and genome assembly improvement. PLoS One 9: e112963.](http://paperpile.com/b/rx9jNZ/QCDlx)

[Watanabe, K., G. Toba, M. Koganezawa, and D. Yamamoto, 2011 Gr39a, a highly diversified gustatory receptor in Drosophila, has a role in sexual behavior. Behav. Genet. 41: 746–753.](http://paperpile.com/b/rx9jNZ/33fL5)

[Weiss, L. A., A. Dahanukar, J. Y. Kwon, D. Banerjee, and J. R. Carlson, 2011 The molecular and cellular basis of bitter taste in Drosophila. Neuron 69: 258–272.](http://paperpile.com/b/rx9jNZ/D3w9Q)

[Whiteman, N. K., A. D. Gloss, T. B. Sackton, S. C. Groen, P. T. Humphrey *et al.*, 2012 Genes involved in the evolution of herbivory by a leaf-mining, Drosophilid fly. Genome Biol. Evol. 4: 900–916.](http://paperpile.com/b/rx9jNZ/zNuhI)

[Willoughby, L., H. Chung, C. Lumb, C. Robin, P. Batterham *et al.*, 2006 A comparison of Drosophila melanogaster detoxification gene induction responses for six insecticides, caffeine and phenobarbital. Insect Biochem. Mol. Biol. 36: 934–942.](http://paperpile.com/b/rx9jNZ/Y9jEW)

[Xu, K., J. R. DiAngelo, M. E. Hughes, J. B. Hogenesch, and A. Sehgal, 2011 The circadian clock interacts with metabolic physiology to influence reproductive fitness. Cell Metab. 13: 639–654.](http://paperpile.com/b/rx9jNZ/IhSmY)

[Yehuda, B.-S., 2012 The Role of DEG/ENaC Subunit ppk8 in Regulating Neuronal Excitability in Drosophila melanogaster. Frontiers in Behavioral Neuroscience 6.:](http://paperpile.com/b/rx9jNZ/ClqdE)

[Zdobnov, E. M., F. Tegenfeldt, D. Kuznetsov, R. M. Waterhouse, F. A. Simão *et al.*, 2017 OrthoDB v9.1: cataloging evolutionary and functional annotations for animal, fungal, plant, archaeal, bacterial and viral orthologs. Nucleic Acids Res. 45: D744–D749.](http://paperpile.com/b/rx9jNZ/6GmpU)

# **LIST OF SUPPORTING DATASETS**

Source data, scripts, and analysis output files are accessible as Supporting Datasets 1-4 in the Dryad repository (https://doi.org/10.6078/D14D8P).

**Dataset 1.** Gene coordinates and identification numbers of chemosensory and detoxification genes curated from *Drosophila* and *Scaptomyza* genome assemblies.

## **Dataset 2.** Data and code used to analyze gene family evolutionary dynamics presented (Figure 2), including curated gene count matrices supplied as input and the raw and parsed output files from the CAFE analysis.

## **Dataset 3.** Data, output, and code for rates of molecular evolution (*dN/dS*, PAML) (Table 1).

## **Dataset 4.** Sequence alignment and phylogeny used to characterize the taxonomic diversity of herbivorous *Scaptomyza* (Figure 1b).
