# Supplementary material for: Evolution of chemosensory and detoxification gene families across herbivorous Drosophilidae
Source: G3 (Bethesda). 2023 Jun 15;13(8):jkad133. doi: 10.1093/g3journal/jkad133 (PMC10411586; doi:10.1093/g3journal/jkad133)
Supplement: jkad133_Supplementary_Data [file jkad133_supplementary_data.zip › File_S2_-_Supplementary_Tables_G3-2023-404324.docx]

# **SUPPLEMENTARY TABLES**

## **Table S1. Species included in analyses and their assembly versions.**

| **Species** | **Assembly version** | **CAFE** | **PAML** |
| --- | --- | --- | --- |
| *Scaptomyza flava* | sfla_v1 |  |  |
| *Scaptomyza flava* | sfla_v2 | **✓** | **✓** |
| *Scaptomyza montana* | iso-CA-L1 | **✓** | **✓** |
| *Scaptomyza graminum* | TMU-2019 | **✓** | **✓** |
| *Scaptomyza pallida* | iso-CA-L1 | **✓** | **✓** |
| *Scaptomyza hsui* | iso-CA-L1 | **✓** | **✓** |
| *Drosophila grimshawi* | dgri_caf1 | **✓** | **✓** |
| *Drosophila mojavensis* | dmoj_caf1 | **✓** | **✓** |
| *Drosophila virilis* | dvir_caf1 | **✓** | **✓** |
| *Drosophila melanogaster* | Release_6_plus_ISO1_MT | **✓** | **✓** |
| *Drosophila ananassae* | dana_caf1 | **✓** |  |
| *Drosophila erecta* | dere_caf1 | **✓** |  |
| *Drosophila pseudoobscura* | Pse_3.0 | **✓** |  |

## **Table S2. *Scaptomyza* genome assembly statistics.**

| **Assembly** | **Sequencing** | **Number of scaffolds** | **Longest scaffold (Mbp)** | **Assembly length (Mbp)** | **N50 (Mbp)** | **L50** | **Automated annotations** |
| --- | --- | --- | --- | --- | --- | --- | --- |
| *S. flava*  (sfla_v2) | PacBio + HiC + Illumina | 781 | 92.65 | 331.7 | 31.83 | 3 | 12,365 |
| *S. flava*  (sfla_v1) | Illumina | 6619 | 2.85 | 216.1 | 0.112 | 404 | 17,997 |
| *S. montana* | Nanopore | 735 | 22.67 | 229.1 | 2.44 | 18 | 11,924 |
| *S. graminum* | Nanopore | 352 | 21.82 | 137.8 | 17.41 | 4 | 11,747 |
| *S. pallida* | Nanopore | 273 | 10.12 | 201.7 | 4.28 | 15 | 11,701 |
| *S. hsui* | Nanopore | 313 | 20.51 | 223.5 | 5.29 | 10 | 11,680 |

## **Table S3. Chemosensory and detoxification gene family sizes across drosophilid genomes.** Gene coordinates for *Scaptomyza* species and gene ID numbers for *Drosophila* species are included in Supplemental Dataset 1.

| **Gene family** | ***Dmel*** | ***Dpse*** | ***Dere*** | ***Dana*** | ***Dvir*** | ***Dmoj*** | ***Dgri*** | ***Spal*** | ***Shsu*** | ***Sgra*** | ***Smon*** | ***Sfla*** |
| --- | --- | --- | --- | --- | --- | --- | --- | --- | --- | --- | --- | --- |
| CYP450 | 89 | 79 | 88 | 93 | 81 | 78 | 85 | 72 | 69 | 68 | 67 | 73 |
| GST | 41 | 36 | 42 | 46 | 34 | 33 | 32 | 29 | 30 | 31 | 34 | 35 |
| UGT | 35 | 29 | 30 | 37 | 29 | 25 | 28 | 26 | 25 | 22 | 22 | 22 |
| GR | 65 | 58 | 56 | 67 | 62 | 61 | 75 | 78 | 72 | 64 | 57 | 61 |
| IR | 58 | 58 | 63 | 62 | 54 | 54 | 59 | 55 | 63 | 52 | 51 | 47 |
| OBP | 52 | 45 | 50 | 50 | 41 | 42 | 50 | 41 | 41 | 33 | 34 | 34 |
| OR | 62 | 65 | 59 | 66 | 56 | 62 | 63 | 68 | 65 | 65 | 60 | 65 |
| PPK | 31 | 30 | 31 | 31 | 29 | 32 | 29 | 31 | 31 | 31 | 31 | 30 |
| TRP | 13 | 16 | 13 | 13 | 13 | 13 | 13 | 13 | 13 | 12 | 11 | 13 |
| Detoxification (all) | 165 | 144 | 160 | 176 | 144 | 136 | 145 | 127 | 124 | 121 | 123 | 130 |
| Chemosensation (all) | 281 | 272 | 272 | 289 | 255 | 264 | 289 | 286 | 285 | 257 | 244 | 250 |
| Random Gene Set | 186 | 183 | 184 | 206 | 212 | 201 | 238 | 196 | 195 | 189 | 193 | 198 |

## **Table S4. Summarized CAFE model output for branch-specific estimates of rates of gene turnover, gene gain and loss across *Drosophila* and *Scaptomyza*.** Gene turnover rates were taken from the two-rate model. Models were run in triplicate, and only the model with the highest log likelihood is shown. Foreground branches: *Dpse* = *D. pseudoobscura,* *Dana* = *D. ananassae*, *Dere* = *D. erecta*, *Dmel* = *D. melanogaster*, *Dmoj* = *D. mojavensis*, *Dvir* = *D. virilis*, *Dgri* = *D. grimshawi*, *Shsu* = *S. hsui*, *Spal* = *S. pallida*, *Sgra* = *S. graminum*, *Smon* = *S. montana*, *Sfla* = *S. flava*, AncH = ancestral branch at the base of all three herbivorous *Scaptomyza*, CladeH = all branches in the herbivorous *Scaptomyza* clade*.* CHE = all chemosensory gene families, DET = all detoxification gene families, RAN = random set of gene families. Other gene family abbreviations as in the main text.

|  |  |  | **Gene turnover rates** | |  |  |  |  |  |
| --- | --- | --- | --- | --- | --- | --- | --- | --- | --- |
| **family** | **branch** | **run** | **Background (lambda0)** | **Foreground (lambda1)** | **lnL (2 rate model)** | **lnL (1 rate model)** | **LRT** | **P value** | **FDR q** |
| CHE | *Dpse* | r3 | 0.002478 | 0.000972 | -1600.45 | -1609.39 | 17.88256 | 2.35E-05 | 0.000282 |
| CHE | *Dana* | r3 | 0.002353 | 0.001792 | -1608.66 | -1609.39 | 1.467542 | 0.225734 | 0.144761 |
| CHE | *Dere* | r3 | 0.002292 | 0.00284 | -1609.16 | -1609.39 | 0.474728 | 0.49082 | 0.222641 |
| CHE | *Dmel* | r1 | 0.002273 | 0.003367 | -1608.48 | -1609.39 | 1.83063 | 0.176053 | 0.125751 |
| CHE | *Dmoj* | r2 | 0.002421 | 0.001126 | -1604.55 | -1609.39 | 9.686002 | 0.001857 | 0.005064 |
| CHE | *Dvir* | r3 | 0.002442 | 0.00093 | -1602.1 | -1609.39 | 14.58796 | 0.000134 | 0.000803 |
| CHE | *Dgri* | r2 | 0.002173 | 0.003767 | -1603.98 | -1609.39 | 10.82308 | 0.001002 | 0.003336 |
| CHE | *Shsu* | r1 | 0.00239 | 0.001161 | -1606.12 | -1609.39 | 6.54671 | 0.010508 | 0.017513 |
| CHE | *Spal* | r3 | 0.002302 | 0.00241 | -1609.37 | -1609.39 | 0.0416 | 0.838384 | 0.318374 |
| CHE | *Sgra* | r1 | 0.002236 | 0.005629 | -1604.03 | -1609.39 | 10.72623 | 0.001056 | 0.003336 |
| CHE | *Smon* | r2 | 0.00225 | 0.009616 | -1601.93 | -1609.39 | 14.93801 | 0.000111 | 0.000763 |
| CHE | *Sfla* | r1 | 0.002246 | 0.008902 | -1603.35 | -1609.39 | 12.08469 | 0.000508 | 0.002076 |
| CHE | AncH | r3 | 0.002227 | 0.004753 | -1604.7 | -1609.39 | 9.3928 | 0.002178 | 0.005446 |
| CHE | CladeH | r2 | 0.001947 | 0.005917 | -1578.97 | -1609.39 | 60.85218 | 6.15E-15 | 3.69E-13 |
| CYP | *Dpse* | r3 | 0.002666 | 0.00068 | -490.567 | -495.022 | 8.910192 | 0.002836 | 0.006544 |
| CYP | *Dana* | r1 | 0.002378 | 0.003565 | -494.272 | -495.022 | 1.500368 | 0.220615 | 0.143879 |
| CYP | *Dere* | r1 | 0.002453 | 0.002966 | -494.956 | -495.022 | 0.13093 | 0.71747 | 0.283212 |
| CYP | *Dmel* | r3 | 0.002516 | 0.001152 | -494.447 | -495.022 | 1.15034 | 0.283478 | 0.160566 |
| CYP | *Dmoj* | r1 | 0.002406 | 0.00316 | -494.658 | -495.022 | 0.727708 | 0.393627 | 0.196814 |
| CYP | *Dvir* | r3 | 0.002628 | 0.00097 | -492.464 | -495.022 | 5.114698 | 0.023724 | 0.031632 |
| CYP | *Dgri* | r2 | 0.002414 | 0.003067 | -494.749 | -495.022 | 0.545016 | 0.460361 | 0.218468 |
| CYP | *Shsu* | r2 | 0.002442 | 0.002898 | -494.919 | -495.022 | 0.20483 | 0.65085 | 0.262087 |
| CYP | *Spal* | r3 | 0.002599 | 0.000406 | -491.58 | -495.022 | 6.883502 | 0.008699 | 0.016312 |
| CYP | *Sgra* | r1 | 0.002481 | 0.002069 | -494.992 | -495.022 | 0.060234 | 0.806126 | 0.308074 |
| CYP | *Smon* | r2 | 0.002429 | 0.007994 | -493.801 | -495.022 | 2.440962 | 0.118204 | 0.098503 |
| CYP | *Sfla* | r1 | 0.00241 | 0.009308 | -493.064 | -495.022 | 3.914968 | 0.047858 | 0.053175 |
| CYP | AncH | r2 | 0.002438 | 0.003529 | -494.76 | -495.022 | 0.522666 | 0.469707 | 0.218468 |
| CYP | CladeH | r2 | 0.002324 | 0.003995 | -493.258 | -495.022 | 3.52772 | 0.060351 | 0.062432 |
| DET | *Dpse* | r1 | 0.002678 | 0.000875 | -884.941 | -891.126 | 12.36903 | 0.000437 | 0.002076 |
| DET | *Dana* | r1 | 0.002348 | 0.004122 | -888.35 | -891.126 | 5.5518 | 0.018462 | 0.025175 |
| DET | *Dere* | r3 | 0.002444 | 0.003797 | -890.451 | -891.126 | 1.349266 | 0.245406 | 0.153379 |
| DET | *Dmel* | r2 | 0.00247 | 0.003034 | -891.001 | -891.126 | 0.249896 | 0.617148 | 0.253623 |
| DET | *Dmoj* | r1 | 0.002484 | 0.002546 | -891.121 | -891.126 | 0.009398 | 0.922771 | 0.33711 |
| DET | *Dvir* | r2 | 0.00259 | 0.00152 | -889.437 | -891.126 | 3.376808 | 0.06612 | 0.06612 |
| DET | *Dgri* | r3 | 0.002456 | 0.002848 | -890.943 | -891.126 | 0.366094 | 0.545141 | 0.231975 |
| DET | *Shsu* | r2 | 0.002521 | 0.002028 | -890.883 | -891.126 | 0.485032 | 0.486152 | 0.222641 |
| DET | *Spal* | r2 | 0.002605 | 0.000523 | -886.119 | -891.126 | 10.01271 | 0.001555 | 0.004664 |
| DET | *Sgra* | r1 | 0.002495 | 0.002253 | -891.108 | -891.126 | 0.034448 | 0.852757 | 0.320482 |
| DET | *Smon* | r3 | 0.002457 | 0.006605 | -889.837 | -891.126 | 2.576302 | 0.108475 | 0.094326 |
| DET | *Sfla* | r1 | 0.002442 | 0.007808 | -888.847 | -891.126 | 4.558102 | 0.032763 | 0.040118 |
| DET | AncH | r2 | 0.002425 | 0.004617 | -889.468 | -891.126 | 3.314468 | 0.068673 | 0.067547 |
| DET | CladeH | r1 | 0.00231 | 0.004378 | -886.542 | -891.126 | 9.167798 | 0.002463 | 0.005911 |
| GR | *Dpse* | r2 | 0.00365 | 0.001074 | -422.036 | -425.447 | 6.822304 | 0.009003 | 0.016368 |
| GR | *Dana* | r1 | 0.00352 | 0.002069 | -424.821 | -425.447 | 1.252098 | 0.263152 | 0.160566 |
| GR | *Dere* | r2 | 0.00323 | 0.008628 | -423.337 | -425.447 | 4.219042 | 0.039973 | 0.047027 |
| GR | *Dmel* | r2 | 0.003302 | 0.005856 | -424.883 | -425.447 | 1.127724 | 0.288261 | 0.161642 |
| GR | *Dmoj* | r3 | 0.003637 | 0.00077 | -422.093 | -425.447 | 6.708198 | 0.009597 | 0.016936 |
| GR | *Dvir* | r1 | 0.00363 | 0.00083 | -421.87 | -425.447 | 7.154402 | 0.007478 | 0.014956 |
| GR | *Dgri* | r3 | 0.003168 | 0.005916 | -423.415 | -425.447 | 4.063412 | 0.043822 | 0.04961 |
| GR | *Shsu* | r3 | 0.003615 | 0.000499 | -421.858 | -425.447 | 7.17794 | 0.007381 | 0.014956 |
| GR | *Spal* | r2 | 0.003431 | 0.003 | -425.398 | -425.447 | 0.097086 | 0.755355 | 0.292395 |
| GR | *Sgra* | r2 | 0.003295 | 0.007017 | -424.447 | -425.447 | 1.99944 | 0.157357 | 0.117109 |
| GR | *Smon* | r3 | 0.003246 | 0.020437 | -421.056 | -425.447 | 8.780728 | 0.003044 | 0.006765 |
| GR | *Sfla* | r1 | 0.003296 | 0.012771 | -423.591 | -425.447 | 3.71067 | 0.054066 | 0.058981 |
| GR | AncH | r1 | 0.003338 | 0.005029 | -425.13 | -425.447 | 0.634228 | 0.425809 | 0.206036 |
| GR | CladeH | r2 | 0.002967 | 0.007189 | -419.996 | -425.447 | 10.90081 | 0.000961 | 0.003336 |
| GST | *Dpse* | r2 | 0.002387 | 0.000862 | -204.641 | -205.874 | 2.466218 | 0.116318 | 0.098342 |
| GST | *Dana* | r2 | 0.00221 | 0.002244 | -205.874 | -205.874 | 0.000628 | 0.980007 | 0.353337 |
| GST | *Dere* | r2 | 0.002178 | 0.003217 | -205.778 | -205.874 | 0.192124 | 0.661155 | 0.264462 |
| GST | *Dmel* | r1 | 0.002133 | 0.004424 | -205.416 | -205.874 | 0.915696 | 0.338608 | 0.183031 |
| GST | *Dmoj* | r3 | 0.002336 | 0.000964 | -204.997 | -205.874 | 1.7546 | 0.185299 | 0.127793 |
| GST | *Dvir* | r1 | 0.002193 | 0.00241 | -205.857 | -205.874 | 0.033526 | 0.854719 | 0.320482 |
| GST | *Dgri* | r3 | 0.002222 | 0.002114 | -205.87 | -205.874 | 0.008382 | 0.927053 | 0.33711 |
| GST | *Shsu* | r1 | 0.002337 | 1.03E-09 | -204.178 | -205.874 | 3.39199 | 0.065514 | 0.06612 |
| GST | *Spal* | r1 | 0.002322 | 6.31E-09 | -204.577 | -205.874 | 2.593832 | 0.107281 | 0.094326 |
| GST | *Sgra* | r2 | 0.002146 | 0.005712 | -205.255 | -205.874 | 1.237456 | 0.265962 | 0.160566 |
| GST | *Smon* | r2 | 0.002152 | 0.00995 | -204.976 | -205.874 | 1.79562 | 0.180243 | 0.125751 |
| GST | *Sfla* | r1 | 0.002137 | 0.010696 | -204.52 | -205.874 | 2.708376 | 0.099823 | 0.091022 |
| GST | AncH | r2 | 0.00212 | 0.005307 | -205.064 | -205.874 | 1.619792 | 0.203121 | 0.136935 |
| GST | CladeH | r1 | 0.001781 | 0.006787 | -199.785 | -205.874 | 12.17858 | 0.000483 | 0.002076 |
| IR | *Dpse* | r2 | 0.00266 | 0.000429 | -353.771 | -358.705 | 9.867476 | 0.001682 | 0.004806 |
| IR | *Dana* | r1 | 0.002474 | 0.001666 | -358.383 | -358.705 | 0.643252 | 0.422536 | 0.206036 |
| IR | *Dere* | r1 | 0.002425 | 0.001987 | -358.673 | -358.705 | 0.064434 | 0.79962 | 0.307546 |
| IR | *Dmel* | r3 | 0.002344 | 0.004422 | -358.107 | -358.705 | 1.195368 | 0.274249 | 0.160566 |
| IR | *Dmoj* | r2 | 0.00249 | 0.00156 | -358.191 | -358.705 | 1.028058 | 0.310615 | 0.171407 |
| IR | *Dvir* | r2 | 0.002552 | 0.000932 | -356.89 | -358.705 | 3.629712 | 0.056757 | 0.059744 |
| IR | *Dgri* | r3 | 0.002307 | 0.003498 | -358.058 | -358.705 | 1.29303 | 0.25549 | 0.158035 |
| IR | *Shsu* | r3 | 0.002355 | 0.003116 | -358.49 | -358.705 | 0.429824 | 0.512075 | 0.222641 |
| IR | *Spal* | r1 | 0.002253 | 0.005128 | -356.568 | -358.705 | 4.274262 | 0.038694 | 0.046433 |
| IR | *Sgra* | r2 | 0.002282 | 0.008019 | -355.744 | -358.705 | 5.922692 | 0.014947 | 0.021874 |
| IR | *Smon* | r1 | 0.002372 | 0.006858 | -357.982 | -358.705 | 1.445788 | 0.229205 | 0.144761 |
| IR | *Sfla* | r1 | 0.002362 | 0.009157 | -357.472 | -358.705 | 2.465488 | 0.116372 | 0.098342 |
| IR | AncH | r1 | 0.002312 | 0.005378 | -357.353 | -358.705 | 2.703564 | 0.100124 | 0.091022 |
| IR | CladeH | r3 | 0.001989 | 0.00646 | -351.612 | -358.705 | 14.18631 | 0.000166 | 0.000903 |
| OBP | *Dpse* | r2 | 0.001751 | 0.001398 | -232.248 | -232.36 | 0.224346 | 0.635748 | 0.257736 |
| OBP | *Dana* | r3 | 0.00161 | 0.00287 | -231.594 | -232.36 | 1.53267 | 0.215712 | 0.143808 |
| OBP | *Dere* | r3 | 0.001643 | 0.003724 | -231.609 | -232.36 | 1.503024 | 0.220207 | 0.143879 |
| OBP | *Dmel* | r3 | 0.001668 | 0.002906 | -232.058 | -232.36 | 0.603462 | 0.43726 | 0.209885 |
| OBP | *Dmoj* | r1 | 0.001837 | 0.000381 | -230.319 | -232.36 | 4.082154 | 0.043338 | 0.04961 |
| OBP | *Dvir* | r1 | 0.001797 | 0.000781 | -231.522 | -232.36 | 1.676526 | 0.195387 | 0.133218 |
| OBP | *Dgri* | r3 | 0.001586 | 0.002966 | -231.306 | -232.36 | 2.107716 | 0.146558 | 0.114661 |
| OBP | *Shsu* | r1 | 0.001836 | 6.84E-11 | -228.832 | -232.36 | 7.056802 | 0.007896 | 0.015284 |
| OBP | *Spal* | r1 | 0.00182 | 1.09E-11 | -229.249 | -232.36 | 6.222672 | 0.012612 | 0.019914 |
| OBP | *Sgra* | r2 | 0.001742 | 9.38E-10 | -231.462 | -232.36 | 1.796278 | 0.180163 | 0.125751 |
| OBP | *Smon* | r2 | 0.001723 | 6.45E-10 | -232.014 | -232.36 | 0.6927 | 0.405248 | 0.199302 |
| OBP | *Sfla* | r1 | 0.001722 | 8.74E-09 | -232.014 | -232.36 | 0.69271 | 0.405244 | 0.199302 |
| OBP | AncH | r2 | 0.001477 | 0.009788 | -224.919 | -232.36 | 14.88138 | 0.000114 | 0.000763 |
| OBP | CladeH | r1 | 0.001515 | 0.004071 | -229.524 | -232.36 | 5.671962 | 0.017238 | 0.024053 |
| OR | *Dpse* | r3 | 0.003389 | 0.000824 | -428.347 | -433.12 | 9.544764 | 0.002005 | 0.005231 |
| OR | *Dana* | r2 | 0.003154 | 0.00233 | -432.895 | -433.12 | 0.449084 | 0.50277 | 0.222641 |
| OR | *Dere* | r1 | 0.003154 | 0.000828 | -432.069 | -433.12 | 2.101566 | 0.147148 | 0.114661 |
| OR | *Dmel* | r3 | 0.003096 | 0.0029 | -433.114 | -433.12 | 0.01128 | 0.915418 | 0.33711 |
| OR | *Dmoj* | r3 | 0.003163 | 0.002376 | -432.876 | -433.12 | 0.48685 | 0.485336 | 0.222641 |
| OR | *Dvir* | r3 | 0.003283 | 0.001058 | -430.834 | -433.12 | 4.571372 | 0.032511 | 0.040118 |
| OR | *Dgri* | r2 | 0.002859 | 0.005858 | -430.617 | -433.12 | 5.006164 | 0.025257 | 0.032243 |
| OR | *Shsu* | r3 | 0.003152 | 0.002257 | -432.853 | -433.12 | 0.532964 | 0.465363 | 0.218468 |
| OR | *Spal* | r1 | 0.00314 | 0.002329 | -432.933 | -433.12 | 0.372782 | 0.541492 | 0.231975 |
| OR | *Sgra* | r3 | 0.002975 | 0.008222 | -431.283 | -433.12 | 3.67349 | 0.055284 | 0.059233 |
| OR | *Smon* | r1 | 0.003023 | 0.010984 | -431.561 | -433.12 | 3.117238 | 0.077468 | 0.073894 |
| OR | *Sfla* | r3 | 0.002983 | 0.014143 | -430.279 | -433.12 | 5.681066 | 0.017149 | 0.024053 |
| OR | AncH | r1 | 0.003082 | 0.003271 | -433.115 | -433.12 | 0.00924 | 0.923421 | 0.33711 |
| OR | CladeH | r1 | 0.002619 | 0.007735 | -424.739 | -433.12 | 16.76148 | 4.24E-05 | 0.000374 |
| PPK | *Dpse* | r2 | 0.000677 | 0.000397 | -78.7597 | -78.9114 | 0.30336 | 0.581784 | 0.240738 |
| PPK | *Dana* | r1 | 0.000699 | 3.13E-10 | -77.7974 | -78.9114 | 2.2279 | 0.135538 | 0.109896 |
| PPK | *Dere* | r3 | 0.000665 | 3.30E-10 | -78.5067 | -78.9114 | 0.809322 | 0.368321 | 0.19374 |
| PPK | *Dmel* | r2 | 0.000664 | 5.68E-10 | -78.5067 | -78.9114 | 0.809336 | 0.368317 | 0.19374 |
| PPK | *Dmoj* | r3 | 0.000663 | 0.000483 | -78.8625 | -78.9114 | 0.097726 | 0.754576 | 0.292395 |
| PPK | *Dvir* | r1 | 0.000607 | 0.001052 | -78.6886 | -78.9114 | 0.44547 | 0.504494 | 0.222641 |
| PPK | *Dgri* | r3 | 0.000607 | 0.001045 | -78.6945 | -78.9114 | 0.433694 | 0.510182 | 0.222641 |
| PPK | *Shsu* | r1 | 0.000695 | 3.77E-12 | -77.8823 | -78.9114 | 2.058058 | 0.151403 | 0.11499 |
| PPK | *Spal* | r1 | 0.000589 | 0.001515 | -78.313 | -78.9114 | 1.196796 | 0.273963 | 0.160566 |
| PPK | *Sgra* | r3 | 0.000612 | 0.002137 | -78.399 | -78.9114 | 1.024772 | 0.31139 | 0.171407 |
| PPK | *Smon* | r1 | 0.000651 | 1.59E-09 | -78.7938 | -78.9114 | 0.235064 | 0.627794 | 0.256242 |
| PPK | *Sfla* | r2 | 0.000604 | 0.005714 | -77.5962 | -78.9114 | 2.63024 | 0.104846 | 0.093892 |
| PPK | AncH | r3 | 0.000619 | 0.00145 | -78.6478 | -78.9114 | 0.527174 | 0.467797 | 0.218468 |
| PPK | CladeH | r3 | 0.000403 | 0.0031 | -73.2531 | -78.9114 | 11.31658 | 0.000768 | 0.002881 |
| RAN | *Dpse* | r1 | 0.002812 | 0.003372 | -1220.05 | -1220.45 | 0.788164 | 0.374656 | 0.19374 |
| RAN | *Dana* | r2 | 0.00279 | 0.003817 | -1219.5 | -1220.45 | 1.88542 | 0.169719 | 0.122689 |
| RAN | *Dere* | r2 | 0.002943 | 0.000615 | -1217.42 | -1220.45 | 6.058722 | 0.013838 | 0.020757 |
| RAN | *Dmel* | r1 | 0.002868 | 0.002867 | -1220.45 | -1220.45 | 0 | 1 | 0.357143 |
| RAN | *Dmoj* | r1 | 0.003006 | 0.001509 | -1217.22 | -1220.45 | 6.44811 | 0.011107 | 0.018011 |
| RAN | *Dvir* | r1 | 0.002968 | 0.0019 | -1218.89 | -1220.45 | 3.11471 | 0.077588 | 0.073894 |
| RAN | *Dgri* | r3 | 0.002591 | 0.007234 | -1209.44 | -1220.45 | 22.0014 | 2.72E-06 | 4.09E-05 |
| RAN | *Shsu* | r2 | 0.00307 | 1.52E-11 | -1200.26 | -1220.45 | 40.36223 | 2.11E-10 | 6.33E-09 |
| RAN | *Spal* | r1 | 0.003055 | 0.000304 | -1208.51 | -1220.45 | 23.8622 | 1.03E-06 | 2.07E-05 |
| RAN | *Sgra* | r2 | 0.002887 | 0.002263 | -1220.26 | -1220.45 | 0.378024 | 0.538663 | 0.231975 |
| RAN | *Smon* | r1 | 0.002878 | 0.001904 | -1220.28 | -1220.45 | 0.33883 | 0.560505 | 0.233544 |
| RAN | *Sfla* | r1 | 0.002826 | 0.006251 | -1218.92 | -1220.45 | 3.042752 | 0.081098 | 0.07603 |
| RAN | AncH | r2 | 0.002984 | 5.21E-05 | -1212.09 | -1220.45 | 16.70506 | 4.37E-05 | 0.000374 |
| RAN | CladeH | r1 | 0.00302 | 0.001742 | -1217.37 | -1220.45 | 6.15574 | 0.013099 | 0.020152 |
| TRP | *Dpse* | r1 | 0.000367 | 0.002743 | -32.2513 | -34.7644 | 5.026268 | 0.024966 | 0.032243 |
| TRP | *Dana* | r3 | 0.000698 | 5.03E-10 | -34.2983 | -34.7644 | 0.932136 | 0.334309 | 0.18235 |
| TRP | *Dere* | r2 | 0.000665 | 1.28E-09 | -34.5946 | -34.7644 | 0.339504 | 0.560116 | 0.233544 |
| TRP | *Dmel* | r3 | 0.000665 | 8.59E-10 | -34.5946 | -34.7644 | 0.339496 | 0.56012 | 0.233544 |
| TRP | *Dmoj* | r2 | 0.000711 | 8.31E-11 | -34.1897 | -34.7644 | 1.14944 | 0.283666 | 0.160566 |
| TRP | *Dvir* | r2 | 0.000712 | 2.88E-10 | -34.1897 | -34.7644 | 1.149438 | 0.283667 | 0.160566 |
| TRP | *Dgri* | r2 | 0.000711 | 3.69E-10 | -34.1896 | -34.7644 | 1.149638 | 0.283625 | 0.160566 |
| TRP | *Shsu* | r3 | 0.000694 | 9.49E-08 | -34.3329 | -34.7644 | 0.86308 | 0.352878 | 0.189042 |
| TRP | *Spal* | r3 | 0.000689 | 3.71E-10 | -34.3859 | -34.7644 | 0.75706 | 0.38425 | 0.19374 |
| TRP | *Sgra* | r1 | 0.00055 | 0.00463 | -33.6036 | -34.7644 | 2.321668 | 0.127583 | 0.104863 |
| TRP | *Smon* | r1 | 0.000432 | 0.030303 | -28.7414 | -34.7644 | 12.04592 | 0.000519 | 0.002076 |
| TRP | *Sfla* | r2 | 0.000651 | 7.12E-09 | -34.7139 | -34.7644 | 0.101076 | 0.750542 | 0.292395 |
| TRP | AncH | r2 | 0.000669 | 1.96E-10 | -34.543 | -34.7644 | 0.442844 | 0.505753 | 0.222641 |
| TRP | CladeH | r1 | 0.000353 | 0.003567 | -31.4631 | -34.7644 | 6.602602 | 0.010183 | 0.017457 |
| UGT | *Dpse* | r1 | 0.003064 | 0.001508 | -188.68 | -189.41 | 1.460858 | 0.226794 | 0.144761 |
| UGT | *Dana* | r1 | 0.00245 | 0.008234 | -185.585 | -189.41 | 7.650946 | 0.005674 | 0.012159 |
| UGT | *Dere* | r1 | 0.002755 | 0.007512 | -188.379 | -189.41 | 2.062396 | 0.150973 | 0.11499 |
| UGT | *Dmel* | r1 | 0.002737 | 0.007108 | -188.414 | -189.41 | 1.99233 | 0.158097 | 0.117109 |
| UGT | *Dmoj* | r3 | 0.00289 | 0.002922 | -189.41 | -189.41 | 0.00043 | 0.983456 | 0.353337 |
| UGT | *Dvir* | r1 | 0.002986 | 0.001987 | -189.18 | -189.41 | 0.461432 | 0.496955 | 0.222641 |
| UGT | *Dgri* | r1 | 0.002869 | 0.003139 | -189.395 | -189.41 | 0.031126 | 0.85996 | 0.320482 |
| UGT | *Shsu* | r2 | 0.002988 | 0.001627 | -189.013 | -189.41 | 0.79364 | 0.373002 | 0.19374 |
| UGT | *Spal* | r3 | 0.002992 | 0.001156 | -188.81 | -189.41 | 1.200398 | 0.273242 | 0.160566 |
| UGT | *Sgra* | r3 | 0.002961 | 1.80E-09 | -188.314 | -189.41 | 2.19204 | 0.138725 | 0.11098 |
| UGT | *Smon* | r1 | 0.002917 | 1.69E-09 | -189.029 | -189.41 | 0.761558 | 0.382841 | 0.19374 |
| UGT | *Sfla* | r2 | 0.002917 | 3.52E-10 | -189.029 | -189.41 | 0.761558 | 0.382841 | 0.19374 |
| UGT | AncH | r2 | 0.002772 | 0.007154 | -188.424 | -189.41 | 1.972274 | 0.160207 | 0.117224 |
| UGT | CladeH | r1 | 0.002942 | 0.002345 | -189.333 | -189.41 | 0.154956 | 0.693844 | 0.275699 |

## **Table S5. Summarized CAFE model output for branch-specific estimates of rates of gene gain and loss across *Drosophila* and *Scaptomyza*.** Gene turnover rates were taken from the two-rate model. Models were run in triplicate, and only the model with the highest log likelihood is shown. Foreground branches: *Dpse* = *D. pseudoobscura,* *Dana* = *D. ananassae*, *Dere* = *D. erecta*, *Dmel* = *D. melanogaster*, *Dmoj* = *D. mojavensis*, *Dvir* = *D. virilis*, *Dgri* = *D. grimshawi*, *Shsu* = *S. hsui*, *Spal* = *S. pallida*, *Sgra* = *S. graminum*, *Smon* = *S. montana*, *Sfla* = *S. flava*, AncH = ancestral branch at the base of all three herbivorous *Scaptomyza*, CladeH = all branches in the herbivorous *Scaptomyza* clade*.* CHE = all chemosensory gene families, DET = all detoxification gene families, RAN = random set of gene families. Other gene family abbreviations as in the main text.

|  |  |  | **Gene duplication rates** | | **Gene loss rates** | |  |  |  |  |  |
| --- | --- | --- | --- | --- | --- | --- | --- | --- | --- | --- | --- |
| **family** | **branch** | **run** | **Background (lambda0)** | **Foreground (lambda1)** | **Background (mu0)** | **Foreground (mu1)** | **lnL (2 rate model)** | **lnL (1 rate model)** | **LRT** | **P value** | **FDR q** |
| CHE | *Dpse* | r1 | 0.002874 | 0.000984 | 0.002066 | 0.000972 | -1596.07 | -1596.07 | 0 | 1 | 0.42517 |
| CHE | *Dana* | r1 | 0.002668 | 0.00289 | 0.002009 | 0.000968 | -1603.19 | -1596.07 | -14.24 | 1 | 0.42517 |
| CHE | *Dere* | r1 | 0.002748 | 0.00136 | 0.001852 | 0.004241 | -1602.03 | -1596.07 | -11.92 | 1 | 0.42517 |
| CHE | *Dmel* | r1 | 0.00265 | 0.004088 | 0.00189 | 0.002783 | -1604.25 | -1596.07 | -16.36 | 1 | 0.42517 |
| CHE | *Dmoj* | r1 | 0.002865 | 0.001266 | 0.001994 | 0.001127 | -1600.48 | -1596.07 | -8.82 | 1 | 0.42517 |
| CHE | *Dvir* | r1 | 0.002929 | 0.000214 | 0.001994 | 0.001433 | -1593.15 | -1596.07 | 5.84 | 0.015666 | 0.019293 |
| CHE | *Dgri* | r1 | 0.002439 | 0.004972 | 0.001905 | 0.002314 | -1598.49 | -1596.07 | -4.84 | 1 | 0.42517 |
| CHE | *Shsu* | r1 | 0.002714 | 0.002357 | 0.002047 | 0.000355 | -1599.35 | -1596.07 | -6.56 | 1 | 0.42517 |
| CHE | *Spal* | r1 | 0.002626 | 0.003865 | 0.00196 | 0.001203 | -1603.08 | -1596.07 | -14.02 | 1 | 0.42517 |
| CHE | *Sgra* | r1 | 0.002616 | 0.006757 | 0.001853 | 0.004585 | -1599.64 | -1596.07 | -7.14 | 1 | 0.42517 |
| CHE | *Smon* | r1 | 0.002694 | 0.005552 | 0.001822 | 0.013604 | -1595.7 | -1596.07 | 0.74 | 0.389661 | 0.219026 |
| CHE | *Sfla* | r1 | 0.002633 | 0.009626 | 0.001877 | 0.007606 | -1599.29 | -1596.07 | -6.44 | 1 | 0.42517 |
| CHE | AncH | r1 | 0.002787 | 0.000652 | 0.001681 | 0.007868 | -1587.63 | -1596.07 | 16.88 | 3.98E-05 | 0.000284 |
| CHE | CladeH | r1 | 0.002516 | 0.004029 | 0.001403 | 0.007576 | -1564.66 | -1605.3 | 81.28 | 1.96E-19 | 1.40E-17 |
| CYP | *Dpse* | r1 | 0.003109 | 0.000738 | 0.002223 | 0.000802 | -489.252 | -493.465 | 8.426 | 0.003699 | 0.008523 |
| CYP | *Dana* | r1 | 0.002634 | 0.005448 | 0.002083 | 0.00165 | -491.313 | -493.465 | 4.304 | 0.038023 | 0.036212 |
| CYP | *Dere* | r1 | 0.00283 | 0.003535 | 0.002053 | 0.002274 | -493.4 | -493.465 | 0.13 | 0.718432 | 0.369184 |
| CYP | *Dmel* | r1 | 0.002847 | 0.002675 | 0.002122 | 1.63E-07 | -491.937 | -493.465 | 3.056 | 0.080439 | 0.062453 |
| CYP | *Dmoj* | r1 | 0.002827 | 0.003202 | 0.001952 | 0.003126 | -492.882 | -493.465 | 1.166 | 0.280225 | 0.173061 |
| CYP | *Dvir* | r1 | 0.002964 | 0.001602 | 0.002244 | 0.000438 | -490.588 | -493.465 | 5.754 | 0.016451 | 0.019865 |
| CYP | *Dgri* | r1 | 0.002639 | 0.005041 | 0.002149 | 0.000977 | -490.713 | -493.465 | 5.504 | 0.018973 | 0.020849 |
| CYP | *Shsu* | r1 | 0.002917 | 0.00175 | 0.001934 | 0.00403 | -491.766 | -493.465 | 3.398 | 0.065276 | 0.052388 |
| CYP | *Spal* | r2 | 0.002911 | 5.07E-11 | 0.002251 | 0.001019 | -489.448 | -493.465 | 8.034 | 0.004591 | 0.008911 |
| CYP | *Sgra* | r1 | 0.002881 | 0.001108 | 0.002053 | 0.003221 | -493.006 | -493.465 | 0.918 | 0.338001 | 0.199528 |
| CYP | *Smon* | r3 | 0.002855 | 3.40E-10 | 0.001969 | 0.016797 | -489.644 | -493.465 | 7.642 | 0.005702 | 0.010023 |
| CYP | *Sfla* | r3 | 0.002738 | 0.02432 | 0.002057 | 5.55E-09 | -489.453 | -493.465 | 8.024 | 0.004616 | 0.008911 |
| CYP | AncH | r2 | 0.002925 | 3.63E-09 | 0.001914 | 0.005813 | -490.428 | -493.465 | 6.074 | 0.013719 | 0.017816 |
| CYP | CladeH | r1 | 0.00281 | 0.003125 | 0.001802 | 0.00489 | -490.3 | -493.465 | 6.33 | 0.011871 | 0.016364 |
| DET | *Dpse* | r1 | 0.003216 | 0.000805 | 0.002165 | 0.000862 | -881.361 | -888.417 | 14.112 | 0.000172 | 0.00082 |
| DET | *Dana* | r1 | 0.002622 | 0.006844 | 0.002018 | 0.001545 | -880.845 | -888.417 | 15.144 | 9.96E-05 | 0.000547 |
| DET | *Dere* | r1 | 0.002901 | 0.004168 | 0.001957 | 0.003882 | -887.607 | -888.417 | 1.62 | 0.203092 | 0.135575 |
| DET | *Dmel* | r1 | 0.002913 | 0.004906 | 0.002043 | 2.20E-10 | -886.366 | -888.417 | 4.102 | 0.042833 | 0.037771 |
| DET | *Dmoj* | r1 | 0.002935 | 0.002231 | 0.00201 | 0.002739 | -887.604 | -888.417 | 1.626 | 0.202257 | 0.135575 |
| DET | *Dvir* | r1 | 0.003013 | 0.00225 | 0.002147 | 0.00077 | -886.213 | -888.417 | 4.408 | 0.035771 | 0.034528 |
| DET | *Dgri* | r1 | 0.002753 | 0.004086 | 0.002126 | 0.001585 | -887.04 | -888.417 | 2.754 | 0.097011 | 0.072941 |
| DET | *Shsu* | r1 | 0.00307 | 0.000966 | 0.001951 | 0.003014 | -885.259 | -888.417 | 6.316 | 0.011965 | 0.016364 |
| DET | *Spal* | r1 | 0.003025 | 0.000426 | 0.002155 | 0.000607 | -883.036 | -888.417 | 10.762 | 0.001036 | 0.003364 |
| DET | *Sgra* | r1 | 0.002887 | 0.001866 | 0.002076 | 0.002747 | -888.169 | -888.417 | 0.496 | 0.481263 | 0.258466 |
| DET | *Smon* | r3 | 0.002914 | 1.64E-10 | 0.002025 | 0.012327 | -884.604 | -888.417 | 7.626 | 0.005753 | 0.010023 |
| DET | *Sfla* | r1 | 0.002793 | 0.013552 | 0.00207 | 0.00171 | -884.939 | -888.417 | 6.956 | 0.008354 | 0.013745 |
| DET | AncH | r1 | 0.002999 | 0.001559 | 0.001833 | 0.006854 | -883.092 | -888.417 | 10.65 | 0.001101 | 0.003418 |
| DET | CladeH | r1 | 0.002837 | 0.003999 | 0.001765 | 0.004806 | -882.657 | -888.417 | 11.52 | 0.000689 | 0.002588 |
| GR | *Dpse* | r1 | 0.004913 | 0.000913 | 0.002423 | 0.0013 | -417.72 | -421.386 | 7.332 | 0.006774 | 0.01152 |
| GR | *Dana* | r2 | 0.004626 | 0.004675 | 0.002416 | 1.13E-10 | -418.765 | -421.386 | 5.242 | 0.022048 | 0.023861 |
| GR | *Dere* | r1 | 0.004608 | 0.002023 | 0.001984 | 0.01336 | -415.203 | -421.386 | 12.366 | 0.000437 | 0.001952 |
| GR | *Dmel* | r1 | 0.004491 | 0.010971 | 0.00221 | 0.00126 | -419.78 | -421.386 | 3.212 | 0.0731 | 0.057378 |
| GR | *Dmoj* | r1 | 0.005011 | 0.001069 | 0.002358 | 0.000684 | -417.34 | -421.386 | 8.092 | 0.004446 | 0.008911 |
| GR | *Dvir* | r1 | 0.004853 | 0.001152 | 0.002469 | 0.001092 | -418.611 | -421.386 | 5.55 | 0.018481 | 0.020626 |
| GR | *Dgri* | r1 | 0.004263 | 0.008228 | 0.00222 | 0.002366 | -419.072 | -421.386 | 4.628 | 0.031454 | 0.031205 |
| GR | *Shsu* | r3 | 0.004692 | 0.002521 | 0.002481 | 1.34E-10 | -418.019 | -421.386 | 6.734 | 0.009459 | 0.014376 |
| GR | *Spal* | r1 | 0.00445 | 0.007555 | 0.002369 | 1.81E-10 | -417.291 | -421.386 | 8.19 | 0.004212 | 0.008911 |
| GR | *Sgra* | r1 | 0.004574 | 0.008854 | 0.00208 | 0.006587 | -419.765 | -421.386 | 3.242 | 0.071773 | 0.056963 |
| GR | *Smon* | r1 | 0.004588 | 0.007088 | 0.002006 | 0.030252 | -414.305 | -421.386 | 14.162 | 0.000168 | 0.00082 |
| GR | *Sfla* | r1 | 0.004533 | 0.01516 | 0.002165 | 0.011094 | -419.468 | -421.386 | 3.836 | 0.050163 | 0.042154 |
| GR | AncH | r2 | 0.004755 | 3.54E-11 | 0.001968 | 0.008549 | -415.936 | -421.386 | 10.9 | 0.000962 | 0.003271 |
| GR | CladeH | r1 | 0.004427 | 0.003239 | 0.001469 | 0.009988 | -407.157 | -421.386 | 28.458 | 9.58E-08 | 1.71E-06 |
| GST | *Dpse* | r1 | 0.003302 | 2.51E-10 | 0.0016 | 0.000796 | -201.569 | -204.714 | 6.29 | 0.012142 | 0.016364 |
| GST | *Dana* | r1 | 0.00268 | 0.006183 | 0.001561 | 1.98E-10 | -201.864 | -204.714 | 5.7 | 0.016965 | 0.019865 |
| GST | *Dere* | r1 | 0.002872 | 0.006681 | 0.00143 | 0.001078 | -204.142 | -204.714 | 1.144 | 0.284809 | 0.173876 |
| GST | *Dmel* | r1 | 0.002546 | 0.004277 | 0.001689 | 0.004645 | -204.515 | -204.714 | 0.398 | 0.528124 | 0.281516 |
| GST | *Dmoj* | r1 | 0.002787 | 0.000901 | 0.001857 | 0.001027 | -204.122 | -204.714 | 1.184 | 0.276543 | 0.173061 |
| GST | *Dvir* | r1 | 0.00299 | 0.002768 | 0.001492 | 0.001148 | -204.674 | -204.714 | 0.08 | 0.777297 | 0.39658 |
| GST | *Dgri* | r1 | 0.00305 | 0.0021 | 0.001473 | 0.001266 | -204.565 | -204.714 | 0.298 | 0.585139 | 0.307321 |
| GST | *Shsu* | r1 | 0.003117 | 4.77E-10 | 0.001517 | 0.000352 | -202.602 | -204.714 | 4.224 | 0.039856 | 0.037459 |
| GST | *Spal* | r2 | 0.003086 | 1.66E-11 | 0.001427 | 0.000331 | -202.654 | -204.714 | 4.12 | 0.042379 | 0.037771 |
| GST | *Sgra* | r1 | 0.00288 | 0.006216 | 0.001435 | 0.00493 | -204.202 | -204.714 | 1.024 | 0.311572 | 0.187018 |
| GST | *Smon* | r1 | 0.002958 | 0.009684 | 0.001327 | 0.011536 | -203.316 | -204.714 | 2.796 | 0.0945 | 0.071808 |
| GST | *Sfla* | r1 | 0.002871 | 0.012382 | 0.001409 | 0.00909 | -203.431 | -204.714 | 2.566 | 0.109183 | 0.081238 |
| GST | AncH | r1 | 0.002854 | 0.00576 | 0.001425 | 0.003556 | -204.151 | -204.714 | 1.126 | 0.28863 | 0.174716 |
| GST | CladeH | r2 | 0.001989 | 0.009158 | 0.001556 | 0.003969 | -198.723 | -204.714 | 11.982 | 0.000537 | 0.002132 |
| IR | *Dpse* | r1 | 0.002763 | 0.00034 | 0.002559 | 0.000501 | -353.681 | -358.644 | 9.926 | 0.00163 | 0.004311 |
| IR | *Dana* | r1 | 0.00252 | 0.002107 | 0.002413 | 0.001362 | -358.258 | -358.644 | 0.772 | 0.379599 | 0.215929 |
| IR | *Dere* | r1 | 0.002501 | 0.003393 | 0.002353 | 0.000521 | -358.225 | -358.644 | 0.838 | 0.359969 | 0.207356 |
| IR | *Dmel* | r3 | 0.002522 | 4.99E-10 | 0.002165 | 0.007919 | -355.434 | -358.644 | 6.42 | 0.011284 | 0.01612 |
| IR | *Dmoj* | r1 | 0.002425 | 0.000627 | 0.00254 | 0.001851 | -357.924 | -358.644 | 1.44 | 0.230139 | 0.152209 |
| IR | *Dvir* | r1 | 0.002689 | 0.000552 | 0.002417 | 0.001262 | -356.557 | -358.644 | 4.174 | 0.041049 | 0.037771 |
| IR | *Dgri* | r1 | 0.002301 | 0.004452 | 0.002308 | 0.002339 | -357.493 | -358.644 | 2.302 | 0.129208 | 0.09449 |
| IR | *Shsu* | r1 | 0.002295 | 0.00546 | 0.002388 | 0.000807 | -355.832 | -358.644 | 5.624 | 0.017716 | 0.020132 |
| IR | *Spal* | r1 | 0.00238 | 0.004653 | 0.002123 | 0.005591 | -356.39 | -358.644 | 4.508 | 0.033737 | 0.03301 |
| IR | *Sgra* | r1 | 0.002376 | 0.00798 | 0.002185 | 0.008013 | -355.678 | -358.644 | 5.932 | 0.014868 | 0.018632 |
| IR | *Smon* | r1 | 0.002418 | 0.013422 | 0.002322 | 2.05E-11 | -356.66 | -358.644 | 3.968 | 0.046373 | 0.039908 |
| IR | *Sfla* | r2 | 0.002224 | 4.75E-06 | 0.002394 | 0.023344 | -355.52 | -358.644 | 6.248 | 0.012433 | 0.016446 |
| IR | AncH | r1 | 0.002485 | 0.00165 | 0.002121 | 0.009452 | -355.26 | -358.644 | 6.768 | 0.009281 | 0.014376 |
| IR | CladeH | r2 | 0.002057 | 0.00309 | 0.001892 | 0.009968 | -348.796 | -358.644 | 19.696 | 9.08E-06 | 9.26E-05 |
| OBP | *Dpse* | r1 | 0.001415 | 0.000551 | 0.00202 | 0.002606 | -230.713 | -231.392 | 1.358 | 0.243884 | 0.158062 |
| OBP | *Dana* | r1 | 0.001303 | 0.003082 | 0.00192 | 0.002637 | -230.718 | -231.392 | 1.348 | 0.245628 | 0.158062 |
| OBP | *Dere* | r1 | 0.001274 | 0.002071 | 0.001992 | 0.005761 | -230.408 | -231.392 | 1.968 | 0.16066 | 0.112507 |
| OBP | *Dmel* | r1 | 0.001249 | 0.003636 | 0.002072 | 0.002205 | -230.811 | -231.392 | 1.162 | 0.281051 | 0.173061 |
| OBP | *Dmoj* | r1 | 0.001251 | 7.31E-11 | 0.002203 | 0.002759 | -230.329 | -231.392 | 2.126 | 0.144818 | 0.103442 |
| OBP | *Dvir* | r3 | 0.001433 | 5.81E-11 | 0.002134 | 0.001109 | -229.638 | -231.392 | 3.508 | 0.061073 | 0.05033 |
| OBP | *Dgri* | r1 | 0.000863 | 0.005371 | 0.002295 | 3.35E-08 | -222.089 | -231.392 | 18.606 | 1.61E-05 | 0.000143 |
| OBP | *Shsu* | r3 | 0.001379 | 4.34E-05 | 0.002227 | 6.40E-11 | -227.926 | -231.392 | 6.932 | 0.008467 | 0.013745 |
| OBP | *Spal* | r1 | 0.001441 | 8.70E-11 | 0.002112 | 7.98E-05 | -228.414 | -231.392 | 5.956 | 0.014667 | 0.018632 |
| OBP | *Sgra* | r3 | 0.001588 | 0.002101 | 0.001985 | 1.97E-10 | -231.495 | -231.392 | -0.206 | 1 | 0.42517 |
| OBP | *Smon* | r2 | 0.001328 | 4.86E-05 | 0.002113 | 1.52E-05 | -231.053 | -231.392 | 0.678 | 0.410276 | 0.225427 |
| OBP | *Sfla* | r2 | 0.001323 | 0.003131 | 0.002107 | 2.80E-07 | -231.361 | -231.392 | 0.062 | 0.803362 | 0.406972 |
| OBP | AncH | r1 | 0.001467 | 8.01E-10 | 0.001485 | 0.016657 | -220.141 | -231.392 | 22.502 | 2.10E-06 | 2.50E-05 |
| OBP | CladeH | r1 | 0.001472 | 0.000906 | 0.001562 | 0.007163 | -226.333 | -231.392 | 10.118 | 0.001468 | 0.004195 |
| OR | *Dpse* | r1 | 0.004585 | 0.001615 | 0.00207 | 0.000419 | -423.588 | -427.648 | 8.12 | 0.004378 | 0.008911 |
| OR | *Dana* | r1 | 0.004285 | 0.004164 | 0.001951 | 0.000733 | -427.009 | -427.648 | 1.278 | 0.258271 | 0.164714 |
| OR | *Dere* | r3 | 0.004548 | 4.46E-10 | 0.001769 | 0.001693 | -425.282 | -427.648 | 4.732 | 0.029606 | 0.029785 |
| OR | *Dmel* | r1 | 0.004267 | 0.005079 | 0.00186 | 0.001402 | -427.58 | -427.648 | 0.136 | 0.71229 | 0.36868 |
| OR | *Dmoj* | r2 | 0.004316 | 0.004014 | 0.001857 | 0.001769 | -427.63 | -427.648 | 0.036 | 0.849515 | 0.424333 |
| OR | *Dvir* | r2 | 0.004611 | 1.28E-09 | 0.001945 | 0.001363 | -422.113 | -427.648 | 11.07 | 0.000877 | 0.003133 |
| OR | *Dgri* | r1 | 0.004046 | 0.006967 | 0.001601 | 0.00494 | -424.337 | -427.648 | 6.622 | 0.010073 | 0.014989 |
| OR | *Shsu* | r1 | 0.004311 | 0.003893 | 0.001932 | 0.000811 | -427.156 | -427.648 | 0.984 | 0.321213 | 0.191198 |
| OR | *Spal* | r1 | 0.004202 | 0.005567 | 0.00196 | 3.28E-08 | -425.232 | -427.648 | 4.832 | 0.027936 | 0.028506 |
| OR | *Sgra* | r2 | 0.004127 | 0.012972 | 0.001795 | 2.80E-10 | -424.784 | -427.648 | 5.728 | 0.016696 | 0.019865 |
| OR | *Smon* | r1 | 0.004375 | 0.006304 | 0.001677 | 0.016227 | -425.149 | -427.648 | 4.998 | 0.025377 | 0.026656 |
| OR | *Sfla* | r1 | 0.00411 | 0.033384 | 0.001816 | 4.60E-09 | -422.82 | -427.648 | 9.656 | 0.001887 | 0.004649 |
| OR | AncH | r1 | 0.004606 | 0.000138 | 0.001618 | 0.003677 | -425.944 | -427.648 | 3.408 | 0.064881 | 0.052388 |
| OR | CladeH | r1 | 0.003734 | 0.00927 | 0.001532 | 0.005383 | -420.036 | -427.648 | 15.224 | 9.55E-05 | 0.000547 |
| PPK | *Dpse* | r1 | 0.000484 | 0.000132 | 0.000847 | 0.000782 | -77.8901 | -78.3199 | 0.8596 | 0.353851 | 0.205488 |
| PPK | *Dana* | r2 | 0.000447 | 0.00298 | 0.001012 | 6.65E-10 | -79.794 | -78.3199 | -2.9482 | 1 | 0.42517 |
| PPK | *Dere* | r1 | 0.00048 | 0.002508 | 0.000799 | 9.39E-07 | -78.7137 | -78.3199 | -0.7876 | 1 | 0.42517 |
| PPK | *Dmel* | r3 | 0.000475 | 1.28E-09 | 0.000857 | 0.002395 | -78.6572 | -78.3199 | -0.6746 | 1 | 0.42517 |
| PPK | *Dmoj* | r1 | 0.000407 | 0.002656 | 0.000918 | 1.63E-10 | -77.936 | -78.3199 | 0.7678 | 0.380899 | 0.215929 |
| PPK | *Dvir* | r2 | 0.00047 | 2.78E-10 | 0.000752 | 0.002009 | -77.1715 | -78.3199 | 2.2968 | 0.129641 | 0.09449 |
| PPK | *Dgri* | r3 | 0.000426 | 4.18E-11 | 0.000768 | 0.001942 | -77.2567 | -78.3199 | 2.1264 | 0.14478 | 0.103442 |
| PPK | *Shsu* | r1 | 0.000475 | 3.11E-11 | 0.001158 | 0.007822 | -83.6806 | -78.3199 | -10.7214 | 1 | 0.42517 |
| PPK | *Spal* | r1 | 0.000392 | 0.001514 | 0.000786 | 0.001516 | -77.6213 | -78.3199 | 1.3972 | 0.237193 | 0.155434 |
| PPK | *Sgra* | r2 | 0.000378 | 0.003643 | 0.000843 | 2.25E-10 | -76.8932 | -78.3199 | 2.8534 | 0.091181 | 0.070031 |
| PPK | *Smon* | r3 | 0.000461 | 3.51E-09 | 0.000837 | 0.001685 | -78.3563 | -78.3199 | -0.0728 | 1 | 0.42517 |
| PPK | *Sfla* | r1 | 0.000465 | 1.04E-09 | 0.000738 | 0.009774 | -76.569 | -78.3199 | 3.5018 | 0.061302 | 0.05033 |
| PPK | AncH | r1 | 0.000477 | 1.99E-10 | 0.00076 | 0.002536 | -77.7372 | -78.3199 | 1.1654 | 0.280348 | 0.173061 |
| PPK | CladeH | r1 | 0.000202 | 0.002972 | 0.000602 | 0.003237 | -72.208 | -78.3199 | 12.2238 | 0.000472 | 0.001983 |
| RAN | *Dpse* | r1 | 0.003544 | 0.003111 | 0.002114 | 0.003456 | -1215.13 | -1217.19 | 4.12 | 0.042379 | 0.037771 |
| RAN | *Dana* | r1 | 0.003326 | 0.005339 | 0.00233 | 0.00126 | -1213.76 | -1217.19 | 6.86 | 0.008815 | 0.013992 |
| RAN | *Dere* | r1 | 0.003591 | 0.000347 | 0.002319 | 0.000732 | -1213.28 | -1217.19 | 7.82 | 0.005167 | 0.009713 |
| RAN | *Dmel* | r1 | 0.003486 | 0.003434 | 0.002248 | 0.002767 | -1217.11 | -1217.19 | 0.16 | 0.689157 | 0.35931 |
| RAN | *Dmoj* | r1 | 0.003598 | 0.001485 | 0.002469 | 0.00118 | -1213.37 | -1217.19 | 7.64 | 0.005709 | 0.010023 |
| RAN | *Dvir* | r1 | 0.003419 | 0.002851 | 0.002547 | 0.000769 | -1214.38 | -1217.19 | 5.62 | 0.017757 | 0.020132 |
| RAN | *Dgri* | r1 | 0.002958 | 0.00947 | 0.002225 | 0.002625 | -1198.52 | -1217.19 | 37.34 | 9.92E-10 | 3.54E-08 |
| RAN | *Shsu* | r2 | 0.002957 | 3.75E-07 | 0.002635 | 6.47E-11 | -1199.93 | -1217.19 | 34.52 | 4.22E-09 | 1.00E-07 |
| RAN | *Spal* | r1 | 0.003668 | 0.000496 | 0.002443 | 8.48E-05 | -1205.43 | -1217.19 | 23.52 | 1.24E-06 | 1.77E-05 |
| RAN | *Sgra* | r1 | 0.003543 | 5.17E-08 | 0.002229 | 0.00396 | -1212.28 | -1217.19 | 9.82 | 0.001726 | 0.004404 |
| RAN | *Smon* | r1 | 0.003508 | 0.001572 | 0.002267 | 0.002299 | -1216.84 | -1217.19 | 0.7 | 0.402784 | 0.223025 |
| RAN | *Sfla* | r1 | 0.003415 | 0.010289 | 0.002258 | 0.001883 | -1214.74 | -1217.19 | 4.9 | 0.026857 | 0.027802 |
| RAN | AncH | r2 | 0.003576 | 0.000305 | 0.002358 | 6.68E-11 | -1208.72 | -1217.19 | 16.94 | 3.86E-05 | 0.000284 |
| RAN | CladeH | r1 | 0.003681 | 0.001555 | 0.00233 | 0.001918 | -1213.1 | -1217.19 | 8.18 | 0.004235 | 0.008911 |
| TRP | *Dpse* | r2 | 0.00016 | 0.010327 | 0.000739 | 0.012642 | -35.1344 | -34.7644 | -0.74 | 1 | 0.42517 |
| TRP | *Dana* | r3 | 0.00063 | 0.006731 | 0.000705 | 5.81E-08 | -36.6751 | -34.7644 | -3.8214 | 1 | 0.42517 |
| TRP | *Dere* | r1 | 0.00064 | 0.002005 | 0.000676 | 1.13E-05 | -34.8587 | -34.7644 | -0.1886 | 1 | 0.42517 |
| TRP | *Dmel* | r3 | 0.000667 | 6.01E-05 | 0.000728 | 9.73E-10 | -34.6143 | -34.7644 | 0.3002 | 0.583757 | 0.307321 |
| TRP | *Dmoj* | r2 | 0.000715 | 3.33E-09 | 0.000687 | 0.004211 | -35.9979 | -34.7644 | -2.467 | 1 | 0.42517 |
| TRP | *Dvir* | r3 | 0.000705 | 0.000336 | 0.000715 | 4.45E-09 | -34.3337 | -34.7644 | 0.8614 | 0.353347 | 0.205488 |
| TRP | *Dgri* | r1 | 0.000806 | 0.001654 | 0.000627 | 4.32E-12 | -34.9504 | -34.7644 | -0.372 | 1 | 0.42517 |
| TRP | *Shsu* | r1 | 0.000682 | 0.00043 | 0.000769 | 0.004947 | -36.0792 | -34.7644 | -2.6296 | 1 | 0.42517 |
| TRP | *Spal* | r2 | 0.000689 | 4.52E-05 | 0.000692 | 3.45E-09 | -34.3988 | -34.7644 | 0.7312 | 0.392494 | 0.219026 |
| TRP | *Sgra* | r3 | 0.000662 | 8.91E-10 | 0.00045 | 0.008801 | -32.8352 | -34.7644 | 3.8584 | 0.049498 | 0.04209 |
| TRP | *Smon* | r3 | 0.000643 | 3.56E-06 | 0.000217 | 0.042931 | -26.99 | -34.7644 | 15.5488 | 8.04E-05 | 0.000522 |
| TRP | *Sfla* | r3 | 0.00065 | 0.002696 | 0.000653 | 1.01E-08 | -34.8189 | -34.7644 | -0.109 | 1 | 0.42517 |
| TRP | AncH | r1 | 0.000629 | 0.003588 | 0.001109 | 5.93E-11 | -35.6017 | -34.7644 | -1.6746 | 1 | 0.42517 |
| TRP | CladeH | r2 | 0.000664 | 0.003953 | 5.23E-11 | 0.013653 | -29.7643 | -34.7644 | 10.0002 | 0.001565 | 0.0043 |
| UGT | *Dpse* | r1 | 0.003449 | 0.001732 | 0.002672 | 0.001088 | -188.223 | -189.09 | 1.734 | 0.187901 | 0.127824 |
| UGT | *Dana* | r1 | 0.002349 | 0.011559 | 0.002526 | 0.003671 | -183.831 | -189.09 | 10.518 | 0.001182 | 0.003518 |
| UGT | *Dere* | r1 | 0.003196 | 0.003119 | 0.002284 | 0.011921 | -187.051 | -189.09 | 4.078 | 0.043445 | 0.037844 |
| UGT | *Dmel* | r2 | 0.002975 | 0.012055 | 0.002499 | 1.16E-09 | -186.492 | -189.09 | 5.196 | 0.022639 | 0.024135 |
| UGT | *Dmoj* | r1 | 0.003422 | 0.001276 | 0.002364 | 0.003843 | -188.15 | -189.09 | 1.88 | 0.170334 | 0.116988 |
| UGT | *Dvir* | r1 | 0.003224 | 0.003375 | 0.002637 | 0.001279 | -188.766 | -189.09 | 0.648 | 0.420829 | 0.227721 |
| UGT | *Dgri* | r1 | 0.003207 | 0.00363 | 0.002505 | 0.002535 | -189.07 | -189.09 | 0.04 | 0.841481 | 0.42328 |
| UGT | *Shsu* | r1 | 0.003474 | 1.62E-10 | 0.002448 | 0.003085 | -187.035 | -189.09 | 4.11 | 0.04263 | 0.037771 |
| UGT | *Spal* | r3 | 0.003342 | 0.001787 | 0.002622 | 1.76E-09 | -188.127 | -189.09 | 1.926 | 0.165197 | 0.114561 |
| UGT | *Sgra* | r3 | 0.003322 | 0.000314 | 0.002568 | 7.44E-10 | -188.056 | -189.09 | 2.068 | 0.150419 | 0.106378 |
| UGT | *Smon* | r2 | 0.003253 | 2.73E-10 | 0.002537 | 0.013236 | -189.584 | -189.09 | -0.988 | 1 | 0.42517 |
| UGT | *Sfla* | r1 | 0.003308 | 1.82E-11 | 0.002588 | 0.000627 | -188.759 | -189.09 | 0.662 | 0.415855 | 0.226747 |
| UGT | AncH | r1 | 0.003329 | 1.19E-09 | 0.002161 | 0.01371 | -184.839 | -189.09 | 8.502 | 0.003548 | 0.008447 |
| UGT | CladeH | r1 | 0.00352 | 1.04E-10 | 0.002299 | 0.005023 | -185.864 | -189.09 | 6.452 | 0.011083 | 0.01612 |

## **Table S6. Orthologous genes identified as significantly expanding or contracting across the drosophilid tree.** Family-wide *P*-value indicates whether significant shifts in turnover rate were detected within a given orthology group. Viterbi *P*-values indicate whether turnover rate was significantly different at individual branches within the phylogeny. Branch IDs (0-22) correspond to the labels shown in Fig. S11. Viterbi *P-*values <0.05 are highlighted in bold black text/pale green cells for all branches, except for the ancestral herbivore branch in which these values are highlighted in white bold text/dark green cells. For genes from the random gene set, the genes are listed by cluster IDs and gene names in parentheses.

|  |  | **Viterbi *P*-values for individual branches** | | | | | | | | | | | | | | | | | | | | | |
| --- | --- | --- | --- | --- | --- | --- | --- | --- | --- | --- | --- | --- | --- | --- | --- | --- | --- | --- | --- | --- | --- | --- | --- |
| **Gene / Cluster ID** | **Family-wide *P*-value** | **0** | **3** | **2** | **4** | **1** | **6** | **5** | **19** | **8** | **17** | **10** | **12** | **11** | **14** | **13** | **16** | **15** | **18** | **9** | **21** | **20** | **22** |
| *G36abc* | 0.0329 | **0.019** | 0.588 | **0.019** | 0.071 | 0.051 | 0.606 | 0.547 | **0.018** | 0.5 | 0.5 | 0.5 | 0.5 | 0.5 | 0.5 | 0.5 | 0.5 | 0.5 | 0.5 | 0.5 | 0.5 | 0.5 | 0.5 |
| *Gr28b* | 0.0003 | 0.572 | 0.547 | 0.528 | **0** | 0.539 | 0.606 | **0.036** | **0.001** | 0.802 | 0.618 | 0.587 | 0.587 | 0.234 | 0.732 | 0.785 | 0.744 | 0.534 | 0.762 | 0.133 | 0.59 | 0.776 | 0.776 |
| *Gr39aA* | 0 | 0.572 | 0.547 | **0.009** | 0.528 | **0.028** | 0.259 | 0.057 | **0.018** | 0.111 | **0.01** | **0.008** | 0.602 | 0.265 | 0.156 | 0.807 | 0.096 | 0.539 | 0.225 | 0.601 | 0.59 | 0.776 | 0.413 |
| *Gr59cd* | 0.028 | 0.63 | 0.588 | 0.554 | 0.554 | 0.574 | 0.682 | 0.588 | 0.123 | 0.22 | 0.133 | 0.571 | 0.571 | 0.629 | 0.232 | 0.186 | **0.024** | 0.534 | 0.386 | **0.004** | 0.557 | 0.703 | 0.291 |
| *Gr85a* | 0 | 0.096 | 0.547 | 0.528 | 0.528 | 0.539 | 0.606 | **0.036** | **0.009** | **0** | 0.601 | 0.111 | **0.029** | 0.654 | **0.003** | 0.759 | 0.31 | 0.528 | 0.161 | 0.109 | 0.574 | 0.744 | 0.357 |
| ***Gr92a93bcd*** | 0.0001 | **0.001** | 0.624 | **0** | 0.103 | 0.605 | 0.737 | 0.117 | 0.592 | 0.652 | 0.544 | 0.555 | 0.069 | 0.602 | 0.642 | **0.046** | 0.609 | 0.512 | 0.622 | 0.544 | 0.539 | **0.028** | 0.083 |
| *Gr98a* | 0.04965 | 0.572 | 0.547 | 0.528 | 0.528 | 0.539 | 0.053 | 0.547 | 0.065 | 0.652 | **0.002** | 0.571 | 0.091 | 0.629 | 0.676 | 0.726 | 0.687 | 0.523 | 0.705 | 0.544 | 0.539 | 0.652 | 0.212 |
| *Ir47abc94abc* | 0.00545 | **0.023** | 0.611 | **0.039** | 0.57 | 0.594 | 0.334 | 0.611 | 0.616 | 0.608 | 0.53 | 0.519 | 0.519 | 0.537 | 0.554 | 0.172 | **0.043** | 0.508 | **0.049** | **0.001** | 0.551 | 0.683 | 0.124 |
| *IR52* | 0.0185 | 0.659 | 0.611 | 0.106 | 0.57 | 0.111 | 0.274 | 0.587 | 0.591 | 0.649 | 0.544 | 0.554 | 0.068 | 0.601 | 0.18 | 0.685 | 0.064 | 0.512 | 0.62 | 0.544 | 0.539 | 0.649 | **0.007** |
| *IR56a* | 0.0001 | 0.549 | 0.532 | 0.519 | 0.519 | 0.527 | 0.574 | 0.532 | 0.534 | 0.559 | **0** | 0.519 | 0.519 | **0.006** | **0.002** | 0.685 | **0.01** | 0.512 | 0.62 | 0.515 | 0.514 | 0.559 | 0.559 |
| *IR60bcdfo* | 0.04565 | 0.078 | 0.587 | 0.082 | **0.029** | 0.574 | 0.677 | 0.092 | **0.035** | 0.559 | 0.515 | 0.519 | 0.519 | 0.537 | 0.554 | 0.576 | 0.062 | 0.504 | 0.546 | 0.515 | 0.514 | 0.559 | 0.559 |
| ***IR67a*** | 0.0693 | 0.549 | 0.532 | 0.519 | **0.01** | 0.527 | 0.574 | 0.532 | 0.534 | 0.559 | 0.515 | 0.537 | 0.537 | 0.571 | **0.03** | **0.017** | 0.541 | 0.504 | 0.546 | 0.515 | 0.514 | 0.559 | 0.559 |
| *IR76a* | 0.0083 | 0.549 | 0.532 | 0.519 | 0.519 | 0.527 | 0.574 | 0.532 | 0.534 | 0.559 | **0.023** | **0.01** | **0.046** | 0.571 | 0.601 | 0.637 | 0.117 | 0.508 | **0.049** | 0.515 | 0.514 | 0.559 | 0.559 |
| *IR7d* | 0.01955 | 0.549 | 0.532 | 0.519 | 0.519 | 0.527 | 0.574 | 0.532 | 0.534 | **0.002** | 0.515 | 0.519 | 0.519 | 0.537 | 0.554 | 0.576 | 0.541 | 0.504 | **0** | 0.515 | 0.514 | 0.559 | 0.559 |
| *IR7e* | 0.0017 | 0.549 | 0.532 | 0.519 | 0.519 | 0.527 | 0.574 | 0.532 | 0.534 | **0** | 0.515 | 0.519 | 0.519 | 0.537 | 0.554 | 0.576 | 0.062 | 0.504 | 0.069 | 0.515 | **0.007** | 0.5 | 0.5 |
| *IR94d* | 0.0478 | 0.549 | 0.532 | 0.519 | 0.519 | 0.527 | 0.574 | 0.532 | 0.534 | 0.089 | 0.515 | 0.519 | **0.023** | 0.537 | 0.066 | 0.576 | 0.541 | 0.504 | 0.546 | 0.515 | 0.514 | 0.089 | 0.559 |
| ***Obp18a*** | 0.0144 | 0.543 | 0.528 | **0.025** | 0.517 | 0.523 | 0.096 | 0.528 | 0.529 | 0.552 | 0.513 | 0.5 | 0.5 | 0.5 | 0.5 | **0.032** | 0.536 | 0.503 | 0.54 | 0.513 | 0.512 | 0.552 | 0.077 |
| *Obp22a* | 0.0204 | 0.064 | 0.528 | **0.008** | 0.517 | 0.523 | 0.096 | 0.528 | **0.044** | 0.5 | 0.5 | 0.5 | 0.5 | 0.5 | 0.5 | 0.5 | 0.5 | 0.5 | 0.5 | 0.5 | 0.5 | 0.5 | 0.5 |
| *Obp51a56fi* | 0.0086 | **0.044** | 0.553 | 0.533 | **0.017** | **0.012** | 0.096 | 0.528 | **0.044** | 0.5 | 0.5 | 0.5 | 0.5 | 0.5 | 0.5 | 0.5 | 0.5 | 0.5 | 0.5 | 0.5 | 0.5 | 0.5 | 0.5 |
| *Obp57ab* | 0.0886 | 0.543 | **0.014** | 0.533 | 0.533 | 0.523 | 0.096 | 0.528 | **0.044** | 0.5 | 0.5 | 0.5 | 0.5 | 0.5 | 0.5 | 0.5 | 0.5 | 0.5 | 0.5 | 0.5 | 0.5 | 0.5 | 0.5 |
| ***Obp58b*** | 0.0192 | 0.543 | 0.528 | 0.517 | 0.517 | 0.523 | 0.565 | 0.528 | 0.529 | **0** | 0.513 | 0.5 | 0.5 | 0.5 | 0.5 | **0.032** | 0.536 | 0.503 | 0.54 | 0.513 | 0.512 | 0.552 | 0.552 |
| ***Obp58c*** | 0.00555 | 0.543 | 0.528 | 0.517 | 0.517 | 0.523 | 0.565 | 0.528 | 0.529 | **0** | 0.513 | 0.5 | 0.5 | 0.5 | 0.5 | **0.032** | 0.536 | 0.503 | 0.54 | 0.513 | 0.512 | 0.552 | 0.552 |
| *Obp99b* | 0.04745 | **0.001** | 0.528 | **0.025** | 0.517 | 0.523 | 0.565 | 0.528 | 0.529 | 0.552 | 0.513 | 0.505 | 0.505 | 0.51 | 0.515 | 0.522 | 0.536 | 0.503 | 0.54 | 0.513 | 0.512 | 0.552 | 0.552 |
| ***Or22ab*** | 0.01125 | **0.003** | 0.579 | **0.018** | 0.549 | 0.567 | 0.666 | 0.056 | 0.544 | 0.107 | 0.52 | 0.5 | 0.5 | 0.5 | 0.5 | **0.043** | 0.072 | 0.505 | 0.56 | 0.52 | 0.518 | 0.577 | 0.577 |
| *Or42b* | 0.02145 | 0.565 | 0.542 | 0.525 | 0.525 | 0.535 | 0.595 | 0.542 | 0.544 | **0** | **0.007** | 0.5 | 0.5 | 0.5 | 0.5 | 0.5 | 0.5 | 0.5 | 0.5 | 0.52 | 0.518 | 0.577 | 0.577 |
| *Or59a* | 0.0316 | 0.565 | 0.542 | 0.525 | 0.525 | 0.535 | 0.595 | 0.542 | 0.06 | 0.687 | 0.558 | 0.521 | 0.521 | **0.003** | 0.651 | 0.696 | 0.192 | 0.515 | 0.652 | 0.053 | 0.535 | 0.074 | 0.195 |
| *Or59bORN2* | 0.00065 | 0.565 | 0.542 | 0.525 | 0.525 | 0.535 | 0.595 | **0.034** | 0.113 | 0.687 | **0.003** | **0.045** | 0.594 | 0.663 | 0.322 | 0.765 | **0.045** | 0.525 | **0.018** | 0.558 | 0.551 | **0.012** | 0.687 |
| ***Or65abc*** | 0.01435 | 0.661 | 0.612 | 0.571 | 0.571 | 0.595 | 0.06 | 0.107 | **0.036** | **0.035** | 0.52 | 0.5 | 0.5 | 0.5 | 0.5 | **0.043** | 0.554 | 0.505 | **0.025** | 0.52 | 0.518 | 0.577 | **0.035** |
| *Or98a85a* | 0.012 | 0.661 | 0.612 | 0.571 | **0.028** | 0.595 | 0.719 | 0.612 | 0.618 | 0.114 | 0.077 | 0.61 | 0.156 | **0.017** | 0.686 | 0.734 | 0.671 | 0.52 | **0.039** | 0.558 | 0.551 | **0.04** | 0.687 |
| *Or98b47a* | 0.08915 | 0.618 | 0.579 | 0.549 | 0.549 | 0.567 | 0.666 | 0.056 | 0.544 | **0.035** | 0.52 | 0.541 | 0.541 | 0.058 | 0.56 | 0.583 | 0.072 | 0.505 | 0.56 | 0.52 | 0.518 | **0.035** | 0.577 |
| *ppk10* | 0.0844 | 0.511 | 0.507 | 0.504 | 0.504 | 0.506 | 0.517 | 0.507 | 0.507 | 0.513 | 0.503 | 0.509 | 0.509 | 0.518 | 0.527 | 0.538 | **0.002** | 0.501 | 0.51 | 0.503 | 0.503 | 0.513 | 0.513 |
| *ppk29* | 0.0563 | 0.511 | 0.507 | 0.504 | 0.504 | 0.506 | **0.021** | 0.507 | 0.507 | 0.513 | 0.503 | 0.509 | 0.509 | 0.518 | 0.527 | 0.538 | 0.509 | 0.501 | 0.51 | 0.503 | 0.503 | 0.513 | **0.016** |
| *ppk8* | 0.00815 | 0.511 | 0.507 | 0.504 | 0.504 | 0.506 | 0.517 | 0.507 | 0.507 | **0.016** | 0.503 | 0.5 | 0.5 | 0.5 | 0.5 | 0.056 | 0.509 | 0.501 | 0.51 | 0.503 | 0.503 | 0.513 | **0.016** |
| *trpγ* | 0.01 | 0.517 | 0.511 | 0.506 | 0.506 | 0.509 | 0.526 | 0.511 | 0.511 | 0.521 | 0.505 | **0.003** | 0.502 | 0.504 | **0.009** | 0.508 | 0.514 | 0.501 | 0.516 | 0.505 | 0.504 | 0.521 | 0.521 |
| *wtrw* | 0.0746 | 0.517 | 0.511 | 0.506 | 0.506 | 0.509 | 0.526 | 0.511 | 0.511 | 0.521 | 0.505 | **0.003** | 0.502 | 0.504 | 0.506 | 0.508 | 0.514 | 0.501 | 0.516 | 0.505 | 0.504 | 0.521 | 0.521 |
| *Pkd2* | 0.071375 | 0.517 | 0.511 | 0.506 | 0.506 | 0.509 | **0** | 0.511 | 0.511 | 0.521 | 0.505 | 0.502 | 0.502 | 0.504 | 0.506 | 0.508 | 0.514 | 0.501 | 0.516 | 0.505 | 0.504 | 0.521 | 0.521 |
| Cluster7 (*Hsp70*) | 0.00015 | 0.758 | 0.697 | 0.635 | 0.083 | 0.095 | **0.008** | 0.674 | 0.681 | 0.204 | **0.003** | 0.525 | 0.525 | 0.548 | **0.001** | 0.595 | 0.645 | 0.517 | 0.66 | 0.597 | **0.049** | 0.784 | **0.018** |
| Cluster8 (*AOX*) | 0.0354 | 0.705 | 0.649 | 0.597 | 0.597 | 0.628 | 0.086 | 0.649 | 0.237 | **0** | 0.562 | 0.525 | 0.525 | 0.548 | 0.569 | 0.595 | 0.645 | 0.517 | 0.66 | 0.562 | 0.555 | 0.296 | 0.694 |
| Cluster16 (*FASN*) | 0.01185 | 0.669 | 0.619 | 0.576 | 0.576 | 0.601 | **0.023** | 0.619 | 0.624 | **0** | 0.094 | 0.517 | 0.517 | 0.533 | 0.548 | 0.567 | 0.605 | 0.511 | 0.617 | 0.562 | 0.555 | 0.694 | 0.694 |
| Cluster30 (CG15270) | 0.0169 | 0.569 | 0.545 | 0.527 | 0.527 | 0.538 | 0.602 | 0.545 | 0.548 | 0.583 | **0** | 0.533 | **0.017** | 0.562 | 0.589 | 0.621 | 0.679 | 0.522 | 0.695 | 0.522 | 0.519 | 0.583 | 0.583 |
| Cluster32 (CG1943) | 0 | **0** | 0.545 | 0.527 | 0.527 | 0.538 | 0.602 | 0.545 | 0.548 | 0.583 | 0.522 | 0.508 | 0.508 | 0.517 | 0.525 | 0.535 | 0.558 | 0.506 | 0.565 | 0.522 | 0.519 | 0.583 | 0.583 |
| Cluster34 (*retn*) | 0.07575 | 0.569 | 0.545 | 0.527 | 0.527 | 0.538 | 0.602 | 0.545 | 0.548 | 0.583 | **0** | 0.533 | 0.533 | 0.562 | 0.589 | 0.621 | 0.679 | 0.522 | 0.695 | 0.522 | 0.519 | 0.583 | 0.583 |
| Cluster36 (*ATP8A*) | 0.07575 | 0.569 | 0.545 | 0.527 | 0.527 | 0.538 | 0.602 | 0.545 | 0.548 | 0.583 | **0** | 0.533 | 0.533 | 0.562 | 0.589 | 0.621 | 0.679 | 0.522 | 0.695 | 0.522 | 0.519 | 0.583 | 0.583 |
| Cluster58 (*Shrm*) | 0.07435 | 0.569 | 0.545 | 0.527 | 0.527 | 0.538 | 0.602 | 0.545 | 0.548 | 0.583 | **0** | 0.525 | **0.013** | 0.548 | 0.569 | 0.595 | 0.645 | 0.517 | 0.66 | 0.522 | 0.519 | 0.583 | 0.583 |
| Cluster87 (CG12896) | 0.06 | 0.072 | 0.585 | 0.553 | **0.028** | 0.571 | 0.261 | 0.585 | 0.589 | 0.086 | 0.543 | 0.508 | 0.508 | **0.049** | 0.548 | 0.567 | 0.605 | 0.511 | 0.617 | 0.543 | 0.057 | 0.583 | 0.583 |
| Cluster104 (*CecC*) | 0.0063 | 0.187 | 0.585 | 0.553 | **0.028** | 0.571 | 0.261 | 0.585 | 0.589 | **0.017** | 0.064 | 0.508 | 0.508 | 0.517 | 0.525 | 0.535 | 0.558 | 0.506 | 0.565 | 0.543 | 0.538 | 0.219 | **0.017** |
| Cluster105 (*Or42a*) | 0.06555 | 0.569 | 0.545 | 0.527 | 0.527 | 0.107 | 0.674 | 0.585 | 0.589 | 0.219 | 0.543 | 0.517 | **0.009** | 0.533 | 0.072 | 0.567 | 0.059 | 0.511 | 0.617 | 0.543 | 0.538 | 0.646 | 0.646 |
| Cluster122 (*Cyp4p1*) | 0.0771 | 0.625 | 0.585 | 0.079 | 0.553 | 0.571 | 0.107 | 0.585 | 0.589 | **0.017** | 0.064 | 0.508 | 0.508 | 0.517 | 0.525 | 0.535 | 0.558 | 0.506 | 0.565 | 0.543 | 0.538 | 0.219 | 0.646 |
| Cluster140 (*qless*) | 0.0201 | 0.569 | 0.545 | 0.527 | 0.527 | 0.538 | 0.602 | 0.545 | 0.548 | 0.583 | **0** | **0.025** | 0.517 | 0.072 | 0.569 | 0.595 | 0.645 | 0.517 | 0.66 | 0.522 | 0.519 | 0.583 | 0.583 |
| Cluster161 (FBgn0052473) | 0.03065 | 0.569 | 0.545 | 0.527 | **0** | 0.538 | 0.602 | 0.127 | 0.589 | **0.017** | 0.064 | 0.508 | 0.508 | 0.517 | 0.525 | 0.535 | 0.558 | 0.506 | 0.565 | 0.543 | 0.538 | 0.646 | 0.646 |
| Cluster171 (*BTBD9*) | 0.06885 | 0.569 | 0.545 | 0.527 | 0.527 | 0.538 | **0** | 0.545 | 0.548 | 0.583 | 0.522 | 0.508 | 0.508 | 0.517 | 0.525 | 0.535 | 0.558 | 0.506 | 0.565 | 0.522 | 0.519 | 0.583 | 0.583 |

## **Table S7. Counts of genes that have been duplicated or lost in all herbivorous *Scaptomyza*.** Counts are also indicated by cell shading. Herbivores are indicated by green font.

|  | **Gene** | ***Dmel*** | ***Dere*** | ***Dana*** | ***Dpse*** | ***Dvir*** | ***Dmoj*** | ***Dgri*** | ***Spal*** | ***Shsu*** | ***Sgra*** | ***Smon*** | ***Sfla*** |  |
| --- | --- | --- | --- | --- | --- | --- | --- | --- | --- | --- | --- | --- | --- | --- |
| **Gr** | ***Gr39aA*** | **1** | **0** | **1** | **3** | **6** | **5** | **4** | **9** | **9** | **7** | **4** | **6** | loss |
|  | ***Gr39aE*** | **1** | **1** | **1** | **2** | **0** | **1** | **1** | **1** | **1** | **0** | **0** | **0** | loss |
|  | ***Gr59ab*** | **2** | **3** | **4** | **4** | **2** | **1** | **4** | **4** | **3** | **2** | **2** | **2** | loss |
|  | ***Gr59cd*** | **2** | **2** | **2** | **2** | **4** | **3** | **7** | **9** | **7** | **3** | **4** | **4** | loss |
|  | ***Gr68a*** | **1** | **1** | **1** | **1** | **1** | **1** | **1** | **1** | **1** | **0** | **0** | **0** | loss |
| **Gst** | ***GstS1*** | **1** | **1** | **1** | **1** | **1** | **1** | **1** | **1** | **1** | **2** | **2** | **2** | **gain** |
| **Ir** | ***Ir47abc94abc*** | **4** | **3** | **6** | **3** | **5** | **4** | **2** | **1** | **3** | **1** | **1** | **1** | loss |
|  | ***Ir51be*** | **1** | **2** | **2** | **2** | **1** | **1** | **1** | **1** | **1** | **0** | **0** | **0** | loss |
|  | ***Ir56e*** | **0** | **0** | **0** | **0** | **1** | **1** | **1** | **2** | **2** | **1** | **1** | **1** | loss |
|  | ***Ir60e*** | **1** | **1** | **1** | **1** | **0** | **1** | **1** | **1** | **1** | **0** | **0** | **0** | loss |
|  | ***Ir67a*** | **0** | **1** | **1** | **1** | **1** | **1** | **1** | **1** | **1** | **2** | **2** | **3** | **gain** |
|  | ***Ir7f*** | **1** | **1** | **1** | **1** | **1** | **1** | **1** | **1** | **1** | **0** | **0** | **0** | loss |
|  | ***Ir94f*** | **1** | **1** | **1** | **0** | **1** | **1** | **1** | **1** | **1** | **0** | **0** | **0** | loss |
| **Obp** | ***Obp18a*** | **1** | **0** | **1** | **0** | **0** | **1** | **1** | **1** | **1** | **0** | **0** | **0** | loss |
|  | ***Obp46a*** | **1** | **1** | **1** | **1** | **1** | **1** | **1** | **1** | **1** | **0** | **0** | **0** | loss |
|  | ***Obp50cd*** | **2** | **2** | **2** | **2** | **1** | **1** | **1** | **1** | **1** | **0** | **0** | **0** | loss |
|  | ***Obp56b*** | **1** | **1** | **1** | **1** | **1** | **1** | **1** | **1** | **1** | **0** | **0** | **0** | loss |
|  | ***Obp58b*** | **1** | **1** | **1** | **1** | **1** | **1** | **4** | **1** | **1** | **0** | **0** | **0** | loss |
|  | ***Obp58c*** | **1** | **1** | **1** | **1** | **1** | **1** | **5** | **1** | **1** | **0** | **0** | **0** | loss |
|  | ***Obp58d*** | **1** | **1** | **1** | **1** | **1** | **1** | **1** | **1** | **1** | **0** | **0** | **0** | loss |
|  | ***Obp93a*** | **1** | **1** | **1** | **1** | **1** | **1** | **1** | **1** | **1** | **0** | **0** | **0** | loss |
| **Or** | ***Or22a*** | **2** | **1** | **5** | **2** | **1** | **1** | **2** | **2** | **1** | **0** | **0** | **0** | loss |
| **P450** | ***Cyp4ad1*** | **1** | **1** | **1** | **1** | **1** | **1** | **1** | **1** | **1** | **0** | **0** | **0** | loss |
|  | ***Cyp4d1*** | **1** | **1** | **2** | **1** | **1** | **2** | **1** | **1** | **1** | **0** | **0** | **0** | loss |
| **Ppk** | ***ppk8*** | **1** | **1** | **1** | **1** | **0** | **1** | **0** | **1** | **1** | **0** | **0** | **0** | loss |
| **Ugt** | ***Ugt302e1*** | **1** | **1** | **0** | **1** | **1** | **1** | **2** | **1** | **1** | **0** | **0** | **0** | loss |

##

## **Table S8. PAML analyses under branch and branch-site models.** Shown are models in which there was a significant difference in selective constraint between the ancestral herbivore branch and background branches. Parentheses surrounding genes indicate that the branch at the base of these genes was evaluated (i.e. genes with multiple paralogs wherein only some paralogs experienced significant shifts in selection). Values in parenthesis to the right of dN/dS values indicate proportion of sites corresponding to the given dN/dS rate.

| **Gene** | **Model** | ***κ*** | **tree length** | **dN/dS** | | | | | | | | **lnL** | **LRT** | ***q-*value *(FDR 5%)*** |
| --- | --- | --- | --- | --- | --- | --- | --- | --- | --- | --- | --- | --- | --- | --- |
| Csp2 | M0 (one ratio) | 1.4 | 5.3 | ω= | 0.07 |  |  |  |  |  |  | -1897.9 | 7.14 | 0.05 |
|  | Branch (two-ratios) | 1.4 | 5.5 | ω0= | 0.06 | ω1= | 0.24 |  |  |  |  | -1894.3 |  |  |
| Gr59e | M0 (one ratio) | 2.1 | 6.7 | ω= | 0.26 |  |  |  |  |  |  | -6683.9 | 18.26 | <0.001 |
|  | Branch (two-ratios) | 2.1 | 7 | ω0= | 0.23 | ω1= | 999 |  |  |  |  | -6674.7 |  |  |
| Gr63a | Branch-site Model A | 1.8 | 3.6 | ω0= | 0.03 (81%) | ω1= | 1 (7%) | ω2a= | 1 (11%) | ω2b= | 1 (1%) | -6110.5 | 16.52 | <0.01 |
|  | Branch-site Model A (ω = 1) | 1.8 | 3.6 | ω0= | 0.03 (0%) | ω1= | 1 (0%) | ω2a= | 1 (92%) | ω2b= | 1 (8%) | -6118.7 |  |  |
| Gr98a | M0 (one ratio) | 1.9 | 16.3 | ω= | 0.29 |  |  |  |  |  |  | -19802.4 | 7.74 | 0.04 |
| (SmonGr98a1, SflaGr98a1, SgraGr98a2) | Branch (two-ratios) | 1.9 | 16.3 | ω0= | 0.29 | ω1= | 0.87 |  |  |  |  | -19798.5 |  |  |
|  | Branch-site Model A | 2 | 17 | ω0= | 0.22 (71%) | ω1= | 1 (26%) | ω2a= | 32.44 (2%) | ω2b= | 32.44 (1%) | -19593 | 11.4 | 0.03 |
|  | Branch-site Model A (ω = 1) | 2 | 16.9 | ω0= | 0.22 (65%) | ω1= | 1 (24%) | ω2a= | 1 (8%) | ω2b= | 1 (3%) | -19598.7 |  |  |
| Gr98bcd | Branch-site Model A | 2.1 | 23.6 | ω0= | 0.16 (73%) | ω1= | 1 (27%) | ω2a | 179.55 (0.004%) | ω2b= | 179.55 (0.001%) | -12238.2 | 10.2 | 0.05 |
|  | Branch-site Model A (ω = 1) | 2.1 | 23.5 | ω0= | 0.16 (73%) | ω1= | 1 (27%) | ω2a | 1 (0%) | ω2b= | 1 (0%) | -12243.3 |  |  |
| GstE9 | M0 (one ratio) | 1.6 | 4.1 | ω= | 0.14 |  |  |  |  |  |  | -3640.7 | 9.35 | 0.02 |
|  | Branch (two-ratios) | 1.6 | 4.1 | ω0= | 0.15 | ω1= | 0.01 |  |  |  |  | -3636 |  |  |
| GstO2 | M0 (one ratio) | 1.8 | 5.5 | ω= | 0.11 |  |  |  |  |  |  | -4112.2 | 10.3 | 0.01 |
|  | Branch (two-ratios) | 1.8 | 5.4 | ω0= | 0.12 | ω1= | 0 |  |  |  |  | -4107.1 |  |  |
| GstS1 | M0 (one ratio) | 2 | 1.9 | ω= | 0.11 |  |  |  |  |  |  | -2833 | 50.43 | <0.0001 |
| (SflaGstS1b, SmonGstS1b, SgraGstS1b) | Branch (two-ratios) | 2 | 1.9 | ω0= | 0.09 | ω1= | 3.27 |  |  |  |  | -2807.8 |  |  |
|  | Branch-site Model A | 2.1 | 1.9 | ω0= | 0.08 (84%) | ω1= | 1 (2%) | ω2a= | 38.47 (13%) | ω2b= | 38.47 (0.004%) | -2800.91 | 14.04 | 0.01 |
|  | Branch-site Model A (ω = 1) | 2 | 1.9 | ω0= | 0.07 (0%) | ω1= | 1 (0%) | ω2a= | 1 (97%) | ω2b= | 1 (3%) | -2807.93 |  |  |
| Ir21a | M0 (one ratio) | 1.5 | 4.7 | ω= | 0.11 |  |  |  |  |  |  | -13380.2 | 9.2 | 0.02 |
|  | Branch (two-ratios) | 1.5 | 4.8 | ω0= | 0.11 | ω1= | 0.26 |  |  |  |  | -13375.6 |  |  |
| Ir48d | M0 (one ratio) | 1.9 | 3.5 | ω= | 0.19 |  |  |  |  |  |  | -8419.1 | 7.96 | 0.04 |
|  | Branch (two-ratios) | 1.9 | 3.5 | ω0= | 0.18 | ω1= | 0.38 |  |  |  |  | -8415.2 |  |  |
| Ir56a | M0 (one ratio) | 1.8 | 15 | ω= | 0.34 |  |  |  |  |  |  | -23071.9 | 18.8 | <0.001 |
| (SflaIr56a, SmonIr56a) | Branch (two-ratios) | 1.8 | 15.1 | ω0= | 0.33 | ω1= | 0.98 |  |  |  |  | -23062.5 |  |  |
| (SgraIr56a1, SgraIr56a2, SgraIr56a4, SgraIr56a5) | Branch (two-ratios) | 1.8 | 15 | ω0= | 0.33 | ω1= | 3.66 |  |  |  |  | -23067.9 | 7.98 | 0.03 |
|  | Branch (two-ratios, fixed ω1 = 1) | 1.8 | 15 | ω0= | 0.34 | ω1= | 1 |  |  |  |  | -23068.7 | 1.56 | 0.31 |
| Ir60a | Branch-site Model A | 1.8 | 7.1 | ω0= | 0.06 (83%) | ω1= | 1 (16%) | ω2a= | 999 (0.005%) | ω2b= | 999 (0.001%) | -10580.9 | 10 | 0.05 |
|  | Branch-site Model A (ω = 1) | 1.8 | 5.4 | ω0= | 0.06 (82%) | ω1= | 1 (16%) | ω2a= | 1 (2%) | ω2b= | 1 (0.003%) | -10585.9 |  |  |
| Ir67a | M0 (one ratio) | 1.8 | 4 | ω= | 0.31 |  |  |  |  |  |  | -10318.5 | 10.6 | 0.01 |
|  | Branch (two-ratios) | 1.8 | 4 | ω0= | 0.3 | ω1= | 0.6 |  |  |  |  | -10313.2 |  |  |
| Obp57cL1 | Branch-site Model A | 2.1 | 9.3 | ω0= | 0.21 (59%) | ω1= | 1 (36%) | ω2a= | 148.51 (3%) | ω2b= | 148.51 (2%) | -2539.7 | 15.68 | <0.01 |
|  | Branch-site Model A (ω = 1) | 2 | 7.3 | ω0= | 0.21 (55%) | ω1= | 1 (30%) | ω2a= | 1 (10%) | ω2b= | 1 (5%) | -2547.5 |  |  |
| Or19a | M0 (one ratio) | 1.7 | 7.6 | ω= | 0.27 |  |  |  |  |  |  | -7966.9 | 13.7 | <0.01 |
|  | Branch (two-ratios) | 1.7 | 7.5 | ω0= | 0.3 | ω1= | 0.09 |  |  |  |  | -7960.1 |  |  |
| Or22c | M0 (one ratio) | 1.6 | 3.7 | ω= | 0.17 |  |  |  |  |  |  | -5823.7 | 7.24 | 0.05 |
|  | Branch (two-ratios) | 1.6 | 3.7 | ω0= | 0.18 | ω1= | 0.05 |  |  |  |  | -5820.1 |  |  |
| Or42a | M0 (one ratio) | 1.7 | 7.5 | ω= | 0.14 |  |  |  |  |  |  | -10294.1 | 11.46 | <0.01 |
| (SflaOr42a1, SmonOr42a1, SgraOr42a1) | Branch (two-ratios) | 1.7 | 7.6 | ω0= | 0.13 | ω1= | 0.45 |  |  |  |  | -10288.4 |  |  |
| Or56a | M0 (one ratio) | 1.5 | 4.3 | ω= | 0.1 |  |  |  |  |  |  | -6120.1 | 8.98 | 0.02 |
|  | Branch (two-ratios) | 1.5 | 4.3 | ω0= | 0.11 | ω1= | 0.03 |  |  |  |  | -6115.6 |  |  |
| Or63a | M0 (one ratio) | 1.6 | 4.7 | ω= | 0.22 |  |  |  |  |  |  | -7544.9 | 29.5 | <0.0001 |
|  | Branch (two-ratios) | 1.6 | 4.8 | ω0= | 0.2 | ω1= | 0.76 |  |  |  |  | -7530.2 |  |  |
| Or67d | M0 (one ratio) | 1.9 | 4.7 | ω= | 0.16 |  |  |  |  |  |  | -4995.1 | 15.18 | <0.01 |
|  | Branch (two-ratios) | 1.9 | 4.5 | ω0= | 0.18 | ω1= | 0.03 |  |  |  |  | -4987.5 |  |  |
| Or85aLike | M0 (one ratio) | 1.5 | 3.9 | ω= | 0.14 |  |  |  |  |  |  | -5773.6 | 6.98 | 0.05 |
|  | Branch (two-ratios) | 1.5 | 4 | ω0= | 0.13 | ω1= | 0.29 |  |  |  |  | -5770.1 |  |  |
| Or85f | Branch-site Model A | 2.1 | 5.5 | ω0= | 0.13 (88%) | ω1= | 1 (11%) | ω2a= | 46.61 (1%) | ω2b= | 46.61 (0.001%) | -5725.5 | 12.54 | 0.03 |
|  | Branch-site Model A (ω = 1) | 2.1 | 5.4 | ω0= | 0.13 (85%) | ω1= | 1 (10%) | ω2a= | 1 (5%) | ω2b= | 1 (1%) | -5731.8 |  |  |
| Or98aLike1 | M0 (one ratio) | 1.6 | 2.9 | ω= | 0.18 |  |  |  |  |  |  | -5564.4 | 7.58 | 0.04 |
|  | Branch (two-ratios) | 1.6 | 2.9 | ω0= | 0.19 | ω1= | 0.05 |  |  |  |  | -5560.6 |  |  |
| Or98aLike2 | M0 (one ratio) | 1.9 | 4.3 | ω= | 0.18 |  |  |  |  |  |  | -5970.6 | 35.92 | <0.0001 |
|  | Branch (two-ratios) | 1.9 | 4.2 | ω0= | 0.2 | ω1= | 0 |  |  |  |  | -5952.7 |  |  |
| OrN2.3prime | Branch-site Model A | 1.9 | 9.2 | ω0= | 0.16 (73%) | ω1= | 1 (20%) | ω2a= | 15.3 (5%) | ω2b= | 15.3 (1%) | -11146.2 | 15.6 | <0.01 |
|  | Branch-site Model A (ω = 1) | 1.9 | 9.2 | ω0= | 0.16 (60%) | ω1= | 1 (17%) | ω2a= | 1 (18%) | ω2b= | 1 (5%) | -11154 |  |  |
| Cyp28a5 | M0 (one ratio) | 1.6 | 4.6 | ω= | 0.12 |  |  |  |  |  |  | -8110 | 9.95 | 0.02 |
|  | Branch (two-ratios) | 1.6 | 4.5 | ω0= | 0.13 | ω1= | 0.05 |  |  |  |  | -8105.1 |  |  |
| Cyp310a1 | M0 (one ratio) | 1.9 | 4.4 | ω= | 0.21 |  |  |  |  |  |  | -8063.8 | 18.43 | <0.001 |
|  | Branch (two-ratios) | 1.9 | 4.4 | ω0= | 0.19 | ω1= | 0.57 |  |  |  |  | -8054.6 |  |  |
| Cyp4d14 | Branch-site Model A | 1.8 | 7.2 | ω0= | 0.06 (84%) | ω1= | 1 (15%) | ω2a= | 809.22 (1%) | ω2b= | 809.22 (0.001%) | -7537.6 | 11.44 | 0.03 |
|  | Branch-site Model A (ω = 1) | 1.8 | 5.3 | ω0= | 0.06 (81%) | ω1= | 1 (15%) | ω2a= | 1 (4%) | ω2b= | 1 (1%) | -7543.3 |  |  |
| Cyp6a16 | M0 (one ratio) | 1.7 | 3.5 | ω= | 0.2 |  |  |  |  |  |  | -8081 | 7.57 | 0.04 |
|  | Branch (two-ratios) | 1.7 | 3.5 | ω0= | 0.21 | ω1= | 0.1 |  |  |  |  | -8077.2 |  |  |
| Cyp6a22 | M0 (one ratio) | 1.6 | 3.4 | ω= | 0.09 |  |  |  |  |  |  | -5558.1 | 21.17 | <0.001 |
|  | Branch (two-ratios) | 1.6 | 3.5 | ω0= | 0.07 | ω1= | 0.26 |  |  |  |  | -5547.5 |  |  |
| Cyp6u1 | M0 (one ratio) | 1.5 | 5.2 | ω= | 0.15 |  |  |  |  |  |  | -8378.5 | 20.06 | <0.001 |
|  | Branch (two-ratios) | 1.6 | 5.3 | ω0= | 0.14 | ω1= | 0.61 |  |  |  |  | -8368.5 |  |  |
| Ppk10 | M0 (one ratio) | 1.5 | 5.4 | ω= | 0.1 |  |  |  |  |  |  | -7191.5 | 12.12 | <0.01 |
|  | Branch (two-ratios) | 1.5 | 5.3 | ω0= | 0.1 | ω1= | 0.01 |  |  |  |  | -7185.4 |  |  |
| Ppk12 | M0 (one ratio) | 1.5 | 5.6 | ω= | 0.19 |  |  |  |  |  |  | -10244.3 | 10.07 | 0.02 |
|  | Branch (two-ratios) | 1.5 | 5.6 | ω0= | 0.18 | ω1= | 0.38 |  |  |  |  | -10239.3 |  |  |
| Ppk13 | M0 (one ratio) | 1.5 | 3.2 | ω= | 0.06 |  |  |  |  |  |  | -5756.3 | 16.12 | <0.01 |
|  | Branch (two-ratios) | 1.5 | 3.3 | ω0= | 0.05 | ω1= | 0.19 |  |  |  |  | -5748.3 |  |  |
| Ppk19 | Branch-site Model A | 1.7 | 6.1 | ω0= | 0.11 (75%) | ω1= | 1 (23%) | ω2a= | 15.75 (2%) | ω2b= | 15.75 (1%) | -8909.7 | 13.98 | 0.01 |
|  | Branch-site Model A (ω = 1) | 1.7 | 6 | ω0= | 0.11 (72%) | ω1= | 1 (23%) | ω2a= | 1 (4%) | ω2b= | 1 (1%) | -8916.7 |  |  |
| Ppk22 | M0 (one ratio) | 1.7 | 4.5 | ω= | 0.16 |  |  |  |  |  |  | -9151.1 | 7.7 | 0.04 |
|  | Branch (two-ratios) | 1.7 | 4.5 | ω0= | 0.16 | ω1= | 0.37 |  |  |  |  | -9147.3 |  |  |
| Ppk25 | M0 (one ratio) | 1.5 | 6.1 | ω= | 0.2 |  |  |  |  |  |  | -8564.4 | 16.82 | <0.01 |
|  | Branch (two-ratios) | 1.5 | 6 | ω0= | 0.21 | ω1= | 0.04 |  |  |  |  | -8556 |  |  |
| Ppk28 | M0 (one ratio) | 1.5 | 3.4 | ω= | 0.1 |  |  |  |  |  |  | -7869.2 | 11.72 | <0.01 |
|  | Branch (two-ratios) | 1.5 | 3.3 | ω0= | 0.1 | ω1= | 0.03 |  |  |  |  | -7863.4 |  |  |
| Ppk30 | M0 (one ratio) | 1.5 | 6.1 | ω= | 0.22 |  |  |  |  |  |  | -8365 | 12.2 | <0.01 |
|  | Branch (two-ratios) | 1.5 | 6.3 | ω0= | 0.2 | ω1= | 0.44 |  |  |  |  | -8358.9 |  |  |
| Ppk31 | M0 (one ratio) | 1.1 | 4 | ω= | 0.13 |  |  |  |  |  |  | -6663 | 17.23 | <0.01 |
|  | Branch (two-ratios) | 1.1 | 4 | ω0= | 0.12 | ω1= | 0.39 |  |  |  |  | -6654.4 |  |  |
| Ppk5 | M0 (one ratio) | 1.5 | 5.4 | ω= | 0.12 |  |  |  |  |  |  | -8397.9 | 15.16 | <0.01 |
|  | Branch (two-ratios) | 1.5 | 5.5 | ω0= | 0.11 | ω1= | 0.36 |  |  |  |  | -8390.3 |  |  |
| Ppk6 | M0 (one ratio) | 1.9 | 3.2 | ω= | 0.1 |  |  |  |  |  |  | -6550 | 10.35 | 0.01 |
|  | Branch (two-ratios) | 1.9 | 3.2 | ω0= | 0.09 | ω1= | 0.25 |  |  |  |  | -6544.9 |  |  |
| Ppk7 | M0 (one ratio) | 1.5 | 3.5 | ω= | 0.16 |  |  |  |  |  |  | -8885.9 | 7.75 | 0.04 |
|  | Branch (two-ratios) | 1.5 | 3.5 | ω0= | 0.15 | ω1= | 0.27 |  |  |  |  | -8882 |  |  |
| nan | M0 (one ratio) | 1.6 | 2.7 | ω= | 0.03 |  |  |  |  |  |  | -8768.9 | 10.66 | 0.01 |
|  | Branch (two-ratios) | 1.6 | 2.7 | ω0= | 0.03 | ω1= | 0.09 |  |  |  |  | -8763.6 |  |  |
|  | Branch-site Model A | 1.8 | 3.6 | ω0= | 0.01 (94%) | ω1= | 1 (6%) | ω2a= | 999 (0.004%) | ω2b= | 999 (0.0002%) | -8618 | 12.2 | 0.03 |
|  | Branch-site Model A (ω = 1) | 1.8 | 2.9 | ω0= | 0.01 (92%) | ω1= | 1 (6%) | ω2a= | 1 (3%) | ω2b= | 1 (0.002%) | -8624.1 |  |  |
| nompC | Branch-site Model A | 1.9 | 2.1 | ω0= | 0.01 (97%) | ω1= | 1 (3%) | ω2a= | 999 (0.001%) | ω2b= | 999 (0.00003%) | -16143.7 | 11.8 | 0.03 |
|  | Branch-site Model A (ω = 1) | 1.9 | 1.9 | ω0= | 0.01 (96%) | ω1= | 1 (3%) | ω2a= | 1 (0.004%) | ω2b= | 1 (0.0001%) | -16149.6 |  |  |
| Ugt301D1 | M0 (one ratio) | 1.8 | 4.2 | ω= | 0.08 |  |  |  |  |  |  | -7343.2 | 7.22 | 0.05 |
|  | Branch (two-ratios) | 1.8 | 4.1 | ω0= | 0.09 | ω1= | 0.03 |  |  |  |  | -7339.6 |  |  |
| Ugt302C1 | M0 (one ratio) | 1.8 | 3.9 | ω= | 0.1 |  |  |  |  |  |  | -7695.6 | 7.42 | 0.05 |
|  | Branch (two-ratios) | 1.8 | 3.9 | ω0= | 0.11 | ω1= | 0.04 |  |  |  |  | -7691.9 |  |  |

## **Table S9. Repetitive element content (as a proportion of genome size) in *S. flava* falls within the range observed across other *Drosophila* genomes.**

| **Species** | **LINE** | **SINE** | **LTR** | **DNA** | **Unclassified** | **SmRNA** | **Others** | **Total (%)** |
| --- | --- | --- | --- | --- | --- | --- | --- | --- |
| *Drosophila melanogaster* | 8,532 | 39 | 14,673 | 5,964 | 9,873 | 257 | 93,764 | 23.71 |
| *Drosophila simulans* | 8,649 | 25 | 11,204 | 5,413 | 9,369 | 27 | 84,572 | 12.04 |
| *Drosophila pseudoobscura* | 11,054 | 82 | 12,481 | 9,418 | 28,827 | 157 | 191,276 | 21.23 |
| *Drosophila virilis* | 15,786 | 0 | 20,749 | 9,133 | 35,155 | 986 | 231,347 | 31.84 |
| *Drosophila mojavensis* | 16,605 | 856 | 18,226 | 27,355 | 48,632 | 125 | 326,217 | 28.64 |
| *Drosophila yakuba* | 13,533 | 0 | 24,306 | 12,327 | 29,688 | 474 | 108,910 | 27.31 |
| *Drosophila erecta* | 17,007 | 0 | 16,968 | 5,845 | 28,264 | 976 | 91,589 | 23.78 |
| *Drosophila anannasse* | 20,405 | 35 | 44,019 | 36,293 | 67,229 | 768 | 124,103 | 44.14 |
| *Drosophila sechellia* | 21,266 | 64 | 19,213 | 6,222 | 21,766 | 1,063 | 97,717 | 28.7 |
| *Drosophila persimilis* | 16,818 | 88 | 24,630 | 16,653 | 48,215 | 271 | 188,702 | 33.06 |
| *Drosophila grimshawi* | 15,538 | 0 | 36,347 | 8,867 | 36,262 | 105 | 281,598 | 30.56 |
| *Scaptomyza flava* (sfla_v1) | 29,063 | 2,208 | 19,457 | 26,651 | 123,393 | 2,241 | 213,342 | 33.82 |

## **Table S10. Gene functions of chemosensory and detoxification genes that were duplicated, lost, or experienced a change in selection regime in the ancestral lineage of herbivorous *Scaptomyza*.** *Indicates that the ancestral herbivore lineage experienced a gene loss or gain, positive selection (dN/dS>1), relaxed purifying selection (foreground branch showed significantly higher dN/dS), or stronger purifying selection (foreground branch showed significantly lower dN/dS) relative to the background branches.

| **Gene** | **Herbivore-**  **specific change*** | **Gene Function in *D. melanogaster*** | **References** |
| --- | --- | --- | --- |
| **Bitter Detection** | |  |  |
| *Gr39aA* | Loss | Involved in the detection of many bitter compounds. | [(Sung *et al.* 2017; Dweck and Carlson 2020)](https://paperpile.com/c/rx9jNZ/eCWLK+nl31R) |
| *Gr59a* | Loss | Putative bitter reception: expressed in labellar S-a and S-b type bitter gustatory neurons. | [(Weiss *et al.* 2011)](https://paperpile.com/c/rx9jNZ/D3w9Q) |
| *Gr59d* | Loss | Bitter detection in larvae. Expressed in labellar S-a and I-a type bitter gustatory neurons. | [(Weiss *et al.* 2011; Kim *et al.* 2016)](https://paperpile.com/c/rx9jNZ/2qYQY+D3w9Q) |
| *Gr98a* | Positive selection | Detects the toxic amino acid histamine, found in high amounts in fermented foods. | [(Aryal and Lee 2022)](https://paperpile.com/c/rx9jNZ/guctm) |
| *Gr98bcd* | Positive selection | Gr98b is required for detection of the toxic plant-derived amino acid L-canavanine. | [(Shim *et al.* 2015)](https://paperpile.com/c/rx9jNZ/LlrVB) |
| *Ir56a* | Relaxed purifying | Coexpressed with bitter neurons. | (Koh et al. 2014) |
| **Yeast/fruit volatile detection** | | |  |
| *Obp18a* | Loss | Involved in detecting diverse yeast/fruit volatiles (propanol, benzaldehyde, citral, 2-heptanone, isoamylacetate, methyl salicylate, phenyl-ethanol, d-carvone). Down-regulated in *D. sechellia.* | [(Dworkin and Jones 2009; Swarup *et al.* 2011)](https://paperpile.com/c/rx9jNZ/cvF9w+LXkp7) |
| *Obp58b* | Loss | Involved in detecting diverse yeast/fruit volatiles (1-hexanol, 2-ethylpyrazine, d-carvone, isoamylacetate, methyl salicylate). | [(Swarup *et al.* 2011)](https://paperpile.com/c/rx9jNZ/cvF9w) |
| *Obp58c* | Loss | Involved in detecting diverse yeast/fruit volatiles (1-hexanol, geraniol, 2-heptanone). | [(Swarup *et al.* 2011)](https://paperpile.com/c/rx9jNZ/cvF9w) |
| *Obp93a* | Loss | Involved in detecting diverse yeast/fruit volatiles (1-hexanol, geraniol, ethylpyrazine, benzaldehyde). | [(Swarup *et al.* 2011)](https://paperpile.com/c/rx9jNZ/cvF9w) |
| *Or19a* | Stronger purifying | Valencene (citrus volatile) detection. Exclusively mediates preference for citrus substrates for egg-laying. | (Dweck et al. 2013) |
| *Or22a* | Loss | Detection of esters and alcohols produced during fermentation by yeasts. | [(Stensmyr *et al.* 2003)](https://paperpile.com/c/rx9jNZ/aNksT) |
| *Or22c* | Stronger purifying | Detection of fruit volatiles in larvae. | (Dweck et al. 2018) |
| *Or85aLike* | Relaxed purifying | *Or85a* detects fatty alcohols produced during fermentation by yeasts. | (Ramasamy et al. 2016) |
| **Other sensory detection** | | |  |
| *Gr39aA* | Loss | Mutant males show reduced courtship. | [(Watanabe *et al.* 2011)](https://paperpile.com/c/rx9jNZ/33fL5) |
| *Gr68a* | Loss | Detection of female sex pheromone. | [(Bray and Amrein 2003)](https://paperpile.com/c/rx9jNZ/ksoYG) |
| *Gr63a* | Relaxed purifying | Carbon dioxide detection. | [(Kwon *et al.* 2007)](https://paperpile.com/c/rx9jNZ/wCOiA) |
| *Ir21a* | Relaxed purifying | Cooling detection and avoidance. | (Ni et al. 2016) |
| *Ppk19* | Positive selection | Detection of high salt concentrations in both larvae and adults. | [(Liu *et al.* 2003; Alves *et al.* 2014)](https://paperpile.com/c/rx9jNZ/yuYdE+Unopg) |
| *Ppk25* | Stronger purifying | Involved in the detection of female pheromone 7,11-heptacosadiene. | (Liu et al. 2018) |
| *Or42a* | Relaxed purifying | Required for the detection of diverse odors in larvae. | (Fishilevich et al. 2005) |
| *Or56a* | Stronger purifying | Detection of geosmin, emitted by harmful microbes (the only known odorant of *Or56a*). | (Chin et al. 2018) |
| *Or67d* | Stronger purifying | Detection of cVA pheromone. | (Kurtovic et al. 2007) |
| *Or85f* | Positive selection | Detects parasitoid wasp (*Leptopilina*) volatiles. Best ligand is the ketone acetophenone. | [(Ebrahim *et al.* 2015)](https://paperpile.com/c/rx9jNZ/NSOoW) |
| *Or98aLike1* | Stronger purifying | *Or98a* detects a broad range of yeast/fruit volatiles, the pyrethrum component (E)-β-farnesene, and mediates copulation. | (Hallem & Carlson 2006; Wang et al. 2021; Sakurai et al. 2013) |
| *Or98aLike2* | Stronger purifying | *Or98a* detects a broad range of yeast/fruit volatiles, the pyrethrum component (E)-β-farnesene, and mediates copulation. | (Hallem & Carlson 2006; Wang et al. 2021; Sakurai et al. 2013) |
| *Ppk28* | Stronger purifying | Sensing pure water or low osmolarity in taste neurons. | (Cameron et al. 2010) |
| *Ppk30* | Relaxed purifying | Mechanosensing and acid sensing. | (Jang et al. 2019) |
| *nan* | Relaxed purifying | Involved in mechanosensation, hearing, and humidity sensing | (Liu et al. 2007) |
| **Detoxification** | | |  |
| *GstE9* | Stronger purifying | Detoxification of 4-hydroxynonenal, a lipid peroxidation product |  |
| *GstS1* | Duplication & positive selection | Detoxification of lipid peroxidation product, 4-HN, oxidation product of adrenalin, adrenochrome, and methylmercury. Expressed in CNS and during development, but highest in flight muscle. | [(Clayton *et al.* 1998; Singh *et al.* 2001; Saisawang *et al.* 2012; Vorojeikina *et al.* 2017)](https://paperpile.com/c/rx9jNZ/FcFEx+T24o7+H05Fk+utcMz) |
| *Cyp4ad1* | Loss | Up-regulated in response to insecticide pyrethroid deltamethrin. Down-regulated by ecdysteroid agonists. Expressed in larval gonads. | [(Davies *et al.* 2006; Liu *et al.* 2020)](https://paperpile.com/c/rx9jNZ/SJEVs+e1Yzr) |
| *Cyp4d1* | Loss | Up-regulated in response to insecticide pyrethroid deltamethrin. Down-regulated by ecdysteroid agonists. Expressed in midgut and fat body. The only P450 that exhibits alternate splicing. | [(Davies *et al.* 2006; Chung *et al.* 2009; Liu *et al.* 2020)](https://paperpile.com/c/rx9jNZ/fJLM1+SJEVs+e1Yzr) |
| *Cyp4d14* | Positive selection | Up-regulated in response to phenobarbital, caffeine, mycotoxins. Expressed in larval midgut. Also lost in *D. sechellia.* | [(Sun *et al.* 2006; Willoughby *et al.* 2006; Chung *et al.* 2009; Trienens *et al.* 2017; Rane *et al.* 2019)](https://paperpile.com/c/rx9jNZ/hHezH+fJLM1+Y9jEW+2vl7V+BP1ps) |
| *Cyp28a5* | Stronger purifying | Up-regulated in response to methanol and the insecticide butene-fipronil. | (Wang et al. 2012; Arain et al. 2018) |
| **Other functions** | | |  |
| *Ir67a* | Duplication | Wing vein development. | [(George *et al.* 2019)](https://paperpile.com/c/rx9jNZ/GeHmy) |
| *Ppk8* | Loss | Involved in regulating neuronal excitability through interactions with the gene seizure. Expressed in embryonic trachea and adult fat body. | [(Xu *et al.* 2011; Yehuda 2012; Suslak 2015)](https://paperpile.com/c/rx9jNZ/ClqdE+IhSmY+bKaIi) |
| *EbpIII*  *(CSP2)* | Relaxed purifying | Involved in pheromone production. | (Montagne & Wicker-Thomas 2021) |
| *Cyp6u1* | Relaxed purifying | Ecdysteroid metabolism. | (Christesen et al. 2017) |

| **Unknown Function in *Drosophila*** | | | | | | | | | | | | | |
| --- | --- | --- | --- | --- | --- | --- | --- | --- | --- | --- | --- | --- | --- |
| *Gr39aE* | Loss |  |  |  |  | *Obp58d* | Loss |  | *Ppk12* | Relaxed purifying |  | *Cyp310a1* | Relaxed purifying |
| *GstO2* | Relaxed purifying |  | *Ir94abc* | Loss |  | *Or63a* | Relaxed purifying |  | *Ppk13* | Relaxed purifying |  | *Ugt301d1* | Stronger purifying |
| *Ir7f* | Loss |  | *Ir94f* | Loss |  | *OrN2.3prime* | Positive selection |  | *Ppk22* | Relaxed purifying |  | *Ugt302c1* | Stronger purifying |
| *Ir48d* | Relaxed purifying |  | *Obp46a* | Loss |  | *Ppk5* | Relaxed purifying |  | *Ppk31* | Relaxed purifying |  | *Ugt302e1* | Loss |
| *Ir51e* | Loss |  | *Obp50cd* | Loss |  | *Ppk6* | Relaxed purifying |  | *Cyp6a16* | Stronger purifying |  |  |  |
| *Ir56e* | Loss |  | *Obp56b* | Loss |  | *Ppk7* | Relaxed purifying |  | *Cyp6a22* | Relaxed purifying |  |  |  |
| *Ir60e* | Loss |  | *Obp57cL1* | Positive selection |  | *Ppk10* | Stronger purifying |  | *Cyp12g1* | Loss |  |  |  |
